# Hippo pathway genes developed varied exon numbers and coevolved functional domains in metazoans for species specific growth control

**DOI:** 10.1186/1471-2148-13-76

**Published:** 2013-04-01

**Authors:** Henan Zhu, Ziwei Zhou, Daxi Wang, Wenyin Liu, Hao Zhu

**Affiliations:** 1 Bioinformatics Section, School of Basic Medical Sciences, Southern Medical University, Guangzhou, 510515, China

## Abstract

**Background:**

The Hippo pathway controls growth by mediating cell proliferation and apoptosis. Dysregulation of Hippo signaling causes abnormal proliferation in both healthy and cancerous cells. The Hippo pathway receives inputs from multiple developmental pathways and interacts with many tissue-specific transcription factors, but how genes in the pathway have evolved remains inadequately revealed.

**Results:**

To explore the origin and evolution of Hippo pathway, we have extensively examined 16 Hippo pathway genes, including upstream regulators and downstream targets, in 24 organisms covering major metazoan phyla. From simple to complex organisms, these genes are varied in the length and number of exons but encode conserved domains with similar higher-order organization. The core of the pathway is more conserved than its upstream regulators and downstream targets. Several components, despite existing in the most basal metazoan sponges, cannot be convincingly identified in other species. Potential recombination breakpoints were identified in some genes. Coevolutionary analysis reveals that most functional domains in Hippo genes have coevolved with interacting functional domains in other genes.

**Conclusions:**

The two essential upstream regulators cadherins *fat* and *dachsous* may have originated in the unicellular organism *Monosiga brevicollis* and evolved more significantly than the core of the pathway. Genes having varied numbers of exons in different species, recombination events, and the gain and loss of some genes indicate alternative splicing and species-specific evolution. Coevolution signals explain some species-specific loss of functional domains. These results significantly unveil the structure and evolution of the Hippo pathway in distant phyla and provide valuable clues for further examination of Hippo signaling.

## Background

Distinct in size and shape, multicellular organisms exhibit a diversity of body plans. In biology, a long-standing question is how the growth and patterning of such body plans, including the organs and tissues within, are controlled by genes during development [1,2]. This question applies to species ranging from the simplest *Amphimedon queenslandica* and *Trichoplax adhaerens*, which lack both organs and internal structures [3,4], to human being. The control of growth and patterning is conducted by a small set of evolutionarily conserved pathways [5], the predominant role of the Hippo pathway in size control in multicellular organisms was only established in 2003 [6-8] and is further recognized recently [9-11].

**Figure 1:**
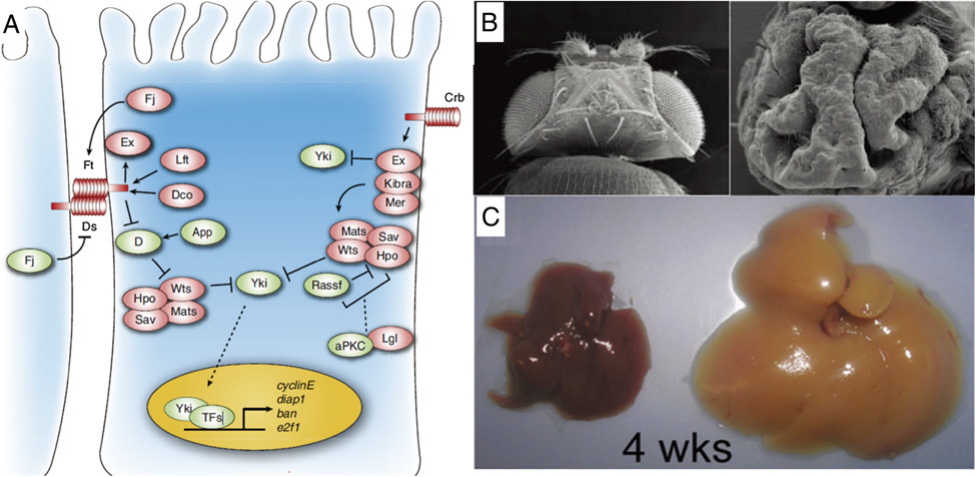
**Structure and function of the Hippo pathway.** (**A**) Hippo pathway in *D. melanogaster* (from [10] with permission). (**B**) A wildtype (left) and an overgrown (right) Drosophila eye caused by a *hippo* mutation (from [7] with permission). (**C**) Mouse livers from a control (left) and an individual with dysregulated Hippo signaling (right) (from [53] with permission).

Growth in Bilateria is specifically regulated by six signaling pathways: RTK signaling via Ras, insulin signaling via the phosphatidylinositol-3-OH kinase pathway, Rheb/Tor, cytokine-JAK/STAT, Warts/Hippo, and the Myc oncogene. All of these pathways, except for the Rheb/Tor pathway, contain genes that are metazoan innovations [4]. Since 2003, researchers have focused more on tissue and organ size control by the newly identified Hippo pathway, which regulates both proliferation and apoptosis (reviewed recently by [9-11]). The first identified Hippo components include the Warts and Hippo kinases, the Salvador and Mats adaptor proteins, and the transcriptional co-activator Yorkie. More recently, a surprising number of upstream regulators have been identified, including the cadherins Fat and Dachsous, which mediate planar cell polarity (PCP), and the proteins lethal giant larvae (Lgl), atypical protein kinase C (aPKC), and Crumbs, which mediate apicobasal cell polarity. The atypical myosin Dachs [12,13] and the FERM-domain proteins Expanded and Merlin relay the Fat/Dachsous signal and the Crumbs signal to the core components Warts/Hippo/Salvador/Mats (Figure 1). Notably, the Hippo pathway has only one transcriptional co-activator (Yorkie in Drosophila and YAP in vertebrates), which interacts with multiple tissue-specific transcription factors, including Scalloped (TEAD in vertebrates) in the wing and Homeothorax in the eye in Drosophila [14,15]. The core Hippo components therefore respond to and coordinate multiple developmental signals [16-18] (recently reviewed by [19]). These features demand intensive investigation into the origin and evolution of Hippo pathway genes.

The Hippo pathway was initially assumed to be a metazoan novelty, because the sole effector Yorkie was not detected in the most basal metazoan *A. queenslandica*[20]. However, as several holozoan genomes were recently sequenced and published, *yorkie* was identified in two non-metazoan lineages: the unicellular amoeboid *Capsaspora owczarzaki* and the choanoflagellate *Monosiga brevicollis*[21]. Remarkably, despite the enormous evolutionary divergence, the transcriptional complex formed by *C. owczarzaki* Scalloped and Yorkie can promote overgrowth of the Drosophila eye [21]. Since previous studies only analyzed a few Hippo genes in a limited number of metazoan phyla [20,21], how genes acting at different positions in the Hippo pathway have evolved across distant phyla and obtained their specific structures remains unclear.

**Figure 2:**
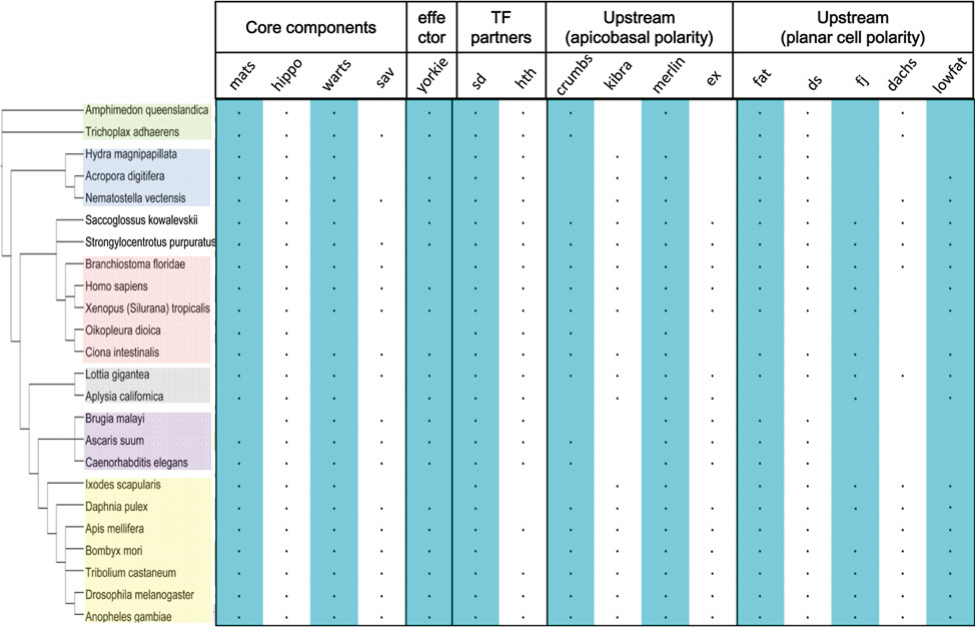
**Schematic representation of the metazoan phylogeny and the distribution of Hippo pathway components.** On the left side, green, blue, pink, grey, purple, and yellow mark Porifera and Placozoa, Cnidaria, Chordata, Gastropoda, Chromadorea, and Arthropoda. On the right side, a dot indicates the presence of orthologs.

Interest in how components in the Hippo pathway interact with each other to control growth in diverse species with distinct body plans, in this study we analyzed the evolution of 16 Hippo pathway genes in 24 metazoans, including a Porifera, a Placozoa, three Cnidaria, and two Gastropoda (Figure 2). Multiple new findings are obtained. During the evolution of Cnidaria from Porifera and Placozoa, while more genes (such as *kibra* and *merlin*) joined the pathway, some others seem to have been lost (such as *expanded*, *dachs*, and *crumbs*) (see also [12]). Compared with genes encoding the key upstream regulators Fat and Dachsous and downstream transcriptional partner Scalloped, the functionally less essential genes, such as *lowfat*, *four-jointed*, and *homeothorax*, show more species-specific (or phyla-, class-, clade-specific) evolution. Core Hippo components are more conserved than their upstream regulators and downstream partners, and in Bilateria they show conserved numbers and organization of domains but varied numbers of exons, indicating that these genes produce alternative splicing while subjecting to purifying selection. Some genes may contain recombination breakpoints. The cadherins Fat and Dachsous*,* which are important for cell-cell adhesion, may have originated in the unicellular organism *Monosiga brevicollis*. We speculate that the Hippo pathway originated primarily to regulate cell adhesion and to control body size in Porifera and Placozoa, and with the addition of more functional domains, upstream regulators, and downstream partners, it acquired control over tissue and organ size during Bilaterian evolution. Coevolutionary analysis indicates that many functional domains in Hippo genes have coevolved with interacting functional domains in other genes. These findings provide valuable clues for further investigations into Hippo signaling mechanisms.

## Results

### Highly conserved core components and species-specific regulators

To examine Hippo pathway evolution across distant metazoan phyla, we searched and compared orthologs of 16 genes in 24 organisms (Figure 2). Despite that Yorkie, the sole effector of Hippo signaling, was previously identified in the filastereans *C. owczarzaki* and the choanoflagellates *M. brevicollis*[21], we did not expect that all, or most, genes can be found in the most basal phyla due to the simplicity of their body plans. Surprisingly, most of the genes were identified in the simplest metazoan *A queenslandica* and *T adhaerens*. On the other hand, Yorkie was not found in *H. magnipapillata*, *O. dioica*, and *I. scapularis* in our search. Yorkie’s main transcriptional partner Scalloped was found in all 24 organisms. The absence of *yorkie* in a given species seems to be accompanied by the absence of multiple other components. For example, in *O. dioica*, orthologs of *salvador*, *kibra*, *expanded*, *fat*, *dachs*, *lowfat*, and *four-jointed* were also not identified. While the core components Mats, Hippo, and Warts are present in almost all species examined, certain upstream regulators and signaling mediators are absent in a considerable number of organisms. The co-existence and co-absence of Hippo components may suggest not only species- and clade-specific evolution but also primary and advanced functional modules. An important feature common to all Hippo pathway genes is the varied numbers of exons and highly conserved functional domains. This is especially apparent for *fat*, a key upstream regulator of Hippo signaling, and should allow genes to produce multiple proteins via alternative splicing to function in different tissues and organs for growth control.

### Domains and exons of the Fat/Dachsous/Dachs family of upstream regulators

The genes *fat* and *dachsous* encode protocadherins required for controlling growth [17] and PCP in Drosophila (reviewed recently by [22,23]). Experimental studies of Drosophila wing and eye growth revealed that Dachsous and Fat may act as a pair of ligand and receptor [24]. The interaction between Fat and Dachsous generates a tissue-level directional cue for planar cell polarization [25,26], and this interaction is modulated by Four-jointed [27-29] and regulates the downstream protein Dachs. Polarized distribution of Dachs in the cell then mediates oriented cell division in Drosophila [13]. The function of Fat and Dachsous in controlling PCP is conserved in mammals [30], suggesting that *dach* should have mammalian orthologs to mediate polarized cell division.

**Figure 3:**
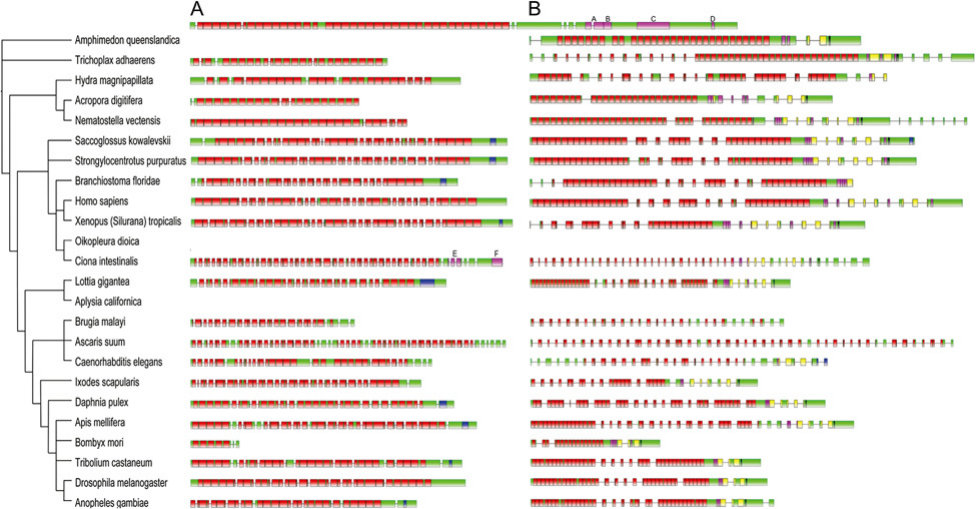
**Genomic structure and functional domains of *dachsous *(A) and *fat *(B) in metazoans.** Exons are indicated as green boxes and functional domains are shown in other colors. In (**A**, **B**), extracellular cadherin repeats (EC repeats) are shown in red, classic cadherin domains (cadherin_C) are shown in blue, and the transmembrane region is shown in black. Letters in (**A**) indicate: A—VSP domain, B—IPT domain, C—REJ domain, D—MYND domain, E—DUF566 domain, F—PROTOCADHERIN domain (A to D are only in *A. queenslandica*). Colors in (**B**) indicate: pink—LamG domain, yellow—EGF domain.

We first examined the distribution of *fat, dachsous, four-jointed,* and *dachs* in species. As a pair of ligand and receptor, Fat and Dachsous co-exist in all examined metazoans except *A. californica* and *O. dioica*, whereas their modifier Four-jointed is absent in multiple organisms, including a Porifera and a Placozoa, three Cnidaria, and three Chromadorea (Figure 3; Additional file 1: Figure S1). This may indicate that Four-jointed joined the growth control and/or PCP mechanisms at a later stage and functions only in Bilateria. As reported, *dachs* encodes an unconventional myosin in Drosophila [12]. Despite being identified in the basal metazoa *A. queenslandica* and *T. adhaerens*, it is absent not only in two Cnidaria (*H. magnipapillata* and *A. digitifera*) and two Nematodes (*A. suum* and *C. elegans*) but also in most Chordates we examined (*H. sapiens*, *X. tropicalis*, *O. dioica*, and *C. intestinalis*). *Dachs* was previously not found in humans [12], but its absence in multiple phyla is somewhat unexpected, raising the questions of how polarized cell division is controlled without *dachs* in epithelial cells and whether *dachs* is dispensible for growth control. In mammals, the myosins closest to Drosophila Dachs are within the myosin V, VII, and X families, but Drosophila Dachs does not seem orthologous to any of these mammalian homologs [12].

We next examined the domains and exons of *fat, dachsous, four-jointed,* and *dachs*. *Fat* has a highly variable number of exons and the characteristic LamG and EGF domains. As cadherins Fat and Dachsous share several notable characteristics: their N-termini each contains many cadherin domains that facilitate heterophilic binding, they have a highly conserved transmembrane region, and their extracellular cadherin repeats (from 20–34 aa in length) appear to be more conserved than the intracellular cadherin domains (Figure 3). As a cadherin-domain kinase, Four-jointed contains a phosphorylation site (contained in the FAM20_C domain) important for the stimulation of Fat-Dachsous binding [29], but this site was not identified in the Porifera, Placozoa, Cnidaria, and Nematoda orthologs. Similar to Fat, Four-jointed has a variable number of exons, from a single exon in Arthropoda and Tetrapoda to many in other organisms (Additional file 1: Figure S1). All known myosins are comprised of an N-terminal head domain, a neck regulatory domain, and a specific C-terminal tail domain [31]. The N-terminal head domain contains a well-conserved ATP binding domain, an actin-binding domain, and an active thiol region; the neck domain has a single calmodulin-type IQ-like motif; and the tail domain is highly divergent (Additional file 1: Figure S2) [12]. Notably, in *dachs* these domains are encoded by significantly varied number of exons (Additional file 1: Figure S2). Compared with the 7 exons present in *D. melanogaster* and *T. castaneum*, *dachs* has more than 20 exons in certain other species, including *A. queenslandica* and *T. adhaerens*, with the number and order of functional domains highly conserved (note that the *dachs* orthologs in *A. queenslandica*, *N. vectensis*, *B. floridae*, and *L. gigantea* lack the long N-terminal extension and the coiled-coil domain). The common feature of conserved domains encoded by highly varied numbers of exons should allow *fat, dachsous, four-jointed*, and *dachs* to produce multiple transcripts for flexible tissue- and species-specific growth control.

The finally examined was the origin of *fat* and *dachsous*. BLASTP searches with the sequences of Drosophila *fat* and *dachsous* against the *A. queenslandica* genome both yielded the EC-containing protein XP_003386184.1 as the highest hit. To determine whether XP_003386184.1 is orthologous to *fat* or to *dachsous*, we built an phylogenetic tree for all *fat* and *dachsous* orthologs together with XP_003386184.1 and found XP_003386184.1 was not convincingly clustered with either group. We then performed a BLASTP search with the sequence of *T. adhaerens dachsous* against the *A. queenslandica* genome and obtained results suggesting that XP_003386184.1 in *A. queenslandica* is more likely orthologous to *dachsous* than to *fat* in *T. adhaerens*. To unveil the origin of *A. queenslandica*’s XP_003386184.1, we used XP_003386184.1 as a query to search (BLASTP) the genome of the choanoflagellate *M. brevicollis* and determined that XP_003386184.1 is similar to XP_001747521.1 and XP_001749260.1, two EC-rich proteins in *M. brevicollis*. Sequence comparisons revealed two findings - the many EC-repeats make *A. queenslandica*’s *fat* and *dachsous* orthologs highly similar to each other, and many EC-containing cadherins in *A. queenslandica* can be identified in *M. brevicollis*. Specifically, the 4900 ~ 6300 aa in the *A. queenslandica*’s *dachsous* is present in the two *M. brevicollis* proteins XP_001747521.1 and XP_001749260.1, but not in *dachsous* orthologs in any other organisms, and can be found in other genes in some metazoans (for example, the A, B, and C domains from 5114 to 6215 aa in *A. queenslandica dachsous* are detected as the main domains in the 2603 aa XP_002612362.1 in *B. floridae*). These findings suggest that *dachsous* may be originated in *M. brevicollis* and possibly underwent a reshuffle from *A. queenslandica* to eumetazoans. Our examination of the *M. brevicollis* genome revealed that it contains up to 23 distinct cadherin genes; among them, the 10056 aa MBCDH21 is the only cadherin with a combination of ECs, LamG, EGF, and transmembrane domains [32]. We found these domains are located at the N-terminus of MBCDH21 in *M. brevicollis*, whereas they fall near the C-terminus in metazoan *fat* orthologs. This discrepancy leaves the issue of whether *fat* originated from MBCDH21 unresolved.

### Domains and exons of Yorkie and its downstream partners

**Figure 4:**
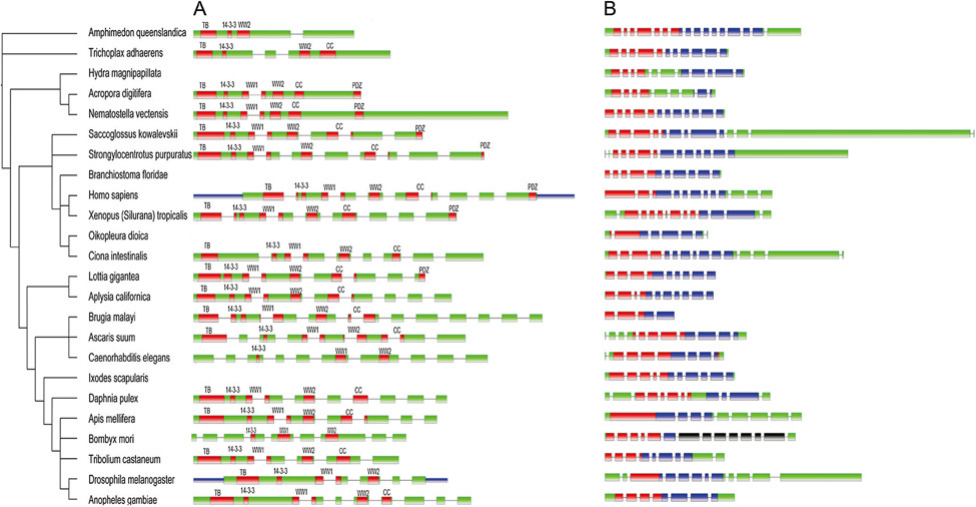
**Genomic structure and functional domains of *yorkie *(A) and *scalloped *(B) in metazoans.** Exons are indicated as green boxes, and functional domains are shown in different colors. Colors in (**B**) indicate: red-TEA domain; black-GLY (Glyco_hydro_30 superfamily) domain, and blue-YBD (yorkie-bound domain).

As the sole effector of Hippo signaling, Yorkie was initially identified in Drosophila and has two homologs in vertebrates, YAP and TAZ [33]. Yorkie contains a TEAD-binding domain (TB domain) at the N-terminus and a transcriptional activation domain at the C-terminus, but lacks a DNA-binding domain (Figure 4). The TB domain enables Yorkie to recognize and bind to Scalloped (TEAD in vertebrates) to activate downstream genes, including *vestigial*, *dE2F1*, and *scalloped* in the Drosophila wing [14]. When the Hippo pathway is activated, Yorkie is phosphorylated by Warts, which promotes the association between Yorkie and 14-3-3 and cytoplasmic retention. Otherwise, Yorkie interacts with Scalloped to form functional heterodimeric transcription factors [34].

A previous analysis reported that *yorkie* appeared after the emergence of *A. queenslandica* and seems to be absent in the Nematodes *C. elegans* and *C. briggsae*[20]. However, in a recent study, *yorkie* orthologs were identified not only in the sponge *A. queenslandica* but also in the unicellular amoeboid *C. owczarzaki*[21]. As Yorkie is the sole downstream effector of Hippo signaling, we reasoned that it should be present in all metazoan phyla. Using human Yap and Drosophila Yorkie as queries, our BLAST search failed to detect *yorkie* orthologs in *L. gigantea*, *A. digitifera*, and *A. californica*, yet GeneWise and GenScan predicted its presence not only in the three organisms but also in *A. queenslandica* and *C. elegans* (Additional file 1: Table S1). In *B. floridae*, due to incomplete genome sequencing, the putative Yorkie ortholog we obtained is very short, 86 aa in length and unlikely representing its true sequence. Unexpectedly, BLAST search, GeneWise, and GenScan did not convincingly find Yorkie orthologs in *H. magnipapillata*, *I. scapularis*, and *O. dioica* (Figure 4).

We next compared the 20 Yorkie orthologs with the human YAP and Drosophila Yorkie sequences. As previously reported [20], we found Yorkie orthologs were more readily comparable to human Yap than to the Drosophila Yorkie in many cases (Additional file 1: Table S2 and Additional file 1: Table S3). For example, the *N. vectensis* Yorkie is more similar to human YAP (similarity = 47%) than to Drosophila Yorkie (similarity = 26.3%), supporting the conjecture that Yorkie sequences in arthropods are much more divergent and harbor many more changes as compared with YAP sequences in Chordates [20]. As reported [20,21], we found that Yorkie contains one WW domain in *A. queenslandica* and *T. adhaerens* and two WW domains in all other phyla. Consisting of a TEF/TEAD-binding motif (TB domain) and a 14-3-3 binding motif, Yorkie’s N-terminus is highly conserved in metazoans, with the exception of absence in *B. mori* (Figure 4). Notably, probably in consequence, Scalloped’s BYD domain in *B. mori* is also very short. The C-terminus of Yorkie, which contains the coiled-coil domain and PDZ-binding motif, is less conserved, as the coiled-coil domain is absent in *A. queenslandica*, *D. melanogaster*, and *C. elegans*, and the PDZ-binding motif is absent in even more organisms (Figure 4A).

Orthologs of *scalloped* were identified in all 24 organisms and significantly varied in the number and length of exons (Figure 4B). We used Jpred, a secondary structure prediction server, with the domains of the human TEAD to analyze Scalloped/TEAD domains in metazoans. All Scalloped orthologs contain a DNA-binding domain (TEA domain, or transcriptional enhancer activator domain) at the N-terminus and a Yorkie-binding domain (YBD domain) at the C-terminus. An exception is the Scalloped ortholog in *B. mori*, which, probably due to the absence of *yorkie* in *B. mori*, contains a very short YBD domain following a GLY domain. Most TEA domains are between 300–450 aa in length, with the shortest comprising 172 aa in *A. digitifera* and the longest 458 aa in *A. queenslandica*. The YBD domains are between 100–200 aa, with the shortest containing 50 aa in *B. mori* and, very remarkably, the longest 252 aa in *A. queenslandica*. A BLAST search indicated that orthologs of *scalloped* are varied in length and divergent in sequence, but Jpred analysis revealed that the secondary structure of their YBD domains is highly conserved (Additional file 1: Figure S4).

Yorkie requires tissue-specific transcription factors to activate different downstream genes. In Drosophila wing Yorkie binds to Scalloped to regulate target genes, but in Drosophila eye Yorkie regulates target genes in conjunction with Homeothorax and Teashirt [15]. We searched for the *homeothorax* ortholog in the 24 organisms and found it exists in all organisms except *I. scapularis*, *D. pulex*, and *B. mori* (Additional file 1: Figure S3), and all these orthologs contain a *homeobox* domain for DNA binding. Similar to *scalloped*, *homeothorax* also varies in the number and length of exons in different organisms (Additional file 1: Figure S3), indicating that this may be a common feature of Yorkie’s partners.

### Phylogenetic analysis of Hippo pathway genes

**Figure 5:**
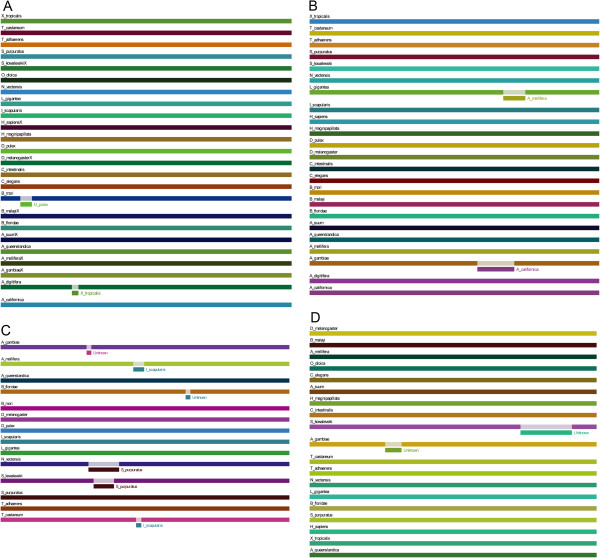
**Detected recombination breakpoints in *scalloped *(A), *warts *(B), *dachs *(C) and *homeothorax *(D).** Recombination breakpoints were detected by > =4 programs in RDP3 (see Methods). Much more recombination breakpoints were detected by > =3 programs (Additional file 1: Figure S5).

Since our analysis suggests that *dachsous* could undergo a reshuffle from *A. queenslandica* to eumetazoans, we examined whether it and other genes contain recombination breakpoints. In all of the 16 genes, no or few recombination breakpoints were detected by > = 4 programs in the RDP package (Figure 5) [35], but considerable ones were detected by > = 3 programs (Additional file 1: Figure S5), leaving the number and sites of recombination breakpoints not reliably determined. We chose seven genes (*crumbs*, *dachsous*, *fat*, *hippo*, *merlin*, *mats*, *yorkie*) that have fewer recombination breakpoints than others for phylogenetic analysis. According to ProtTest [36], the most appropriate substitution models for the *merlin* and *mats* datasets is LG + G, for the *crumbs* dataset is Blosum62 + G + I, and for the *dachsous*, *fat*, *hippo*, and *yorkie*’*s* datasets is VT + G + I, based on the Bayesian information criterion. Since the 24 species cover multiple distinct phyla, the sequences are quite divergent. To make phylogenetic analysis robust, we used two Bayesian packages - PhyloBayes and MrBayes, and two maximum likelihood packages - RAxML and PhyML, to build trees. PhyloBayes produced trees for all the seven genes based on the LG + G model, PhyML produced trees for all the seven genes based on their specific most appropriate substitution models, and MrBayes and RAxML produced trees for *crumbs*, *dachsous*, *fat*, *hippo*, and *yorkie* based on their specific most appropriate substitution models [37-40].

**Figure 6:**
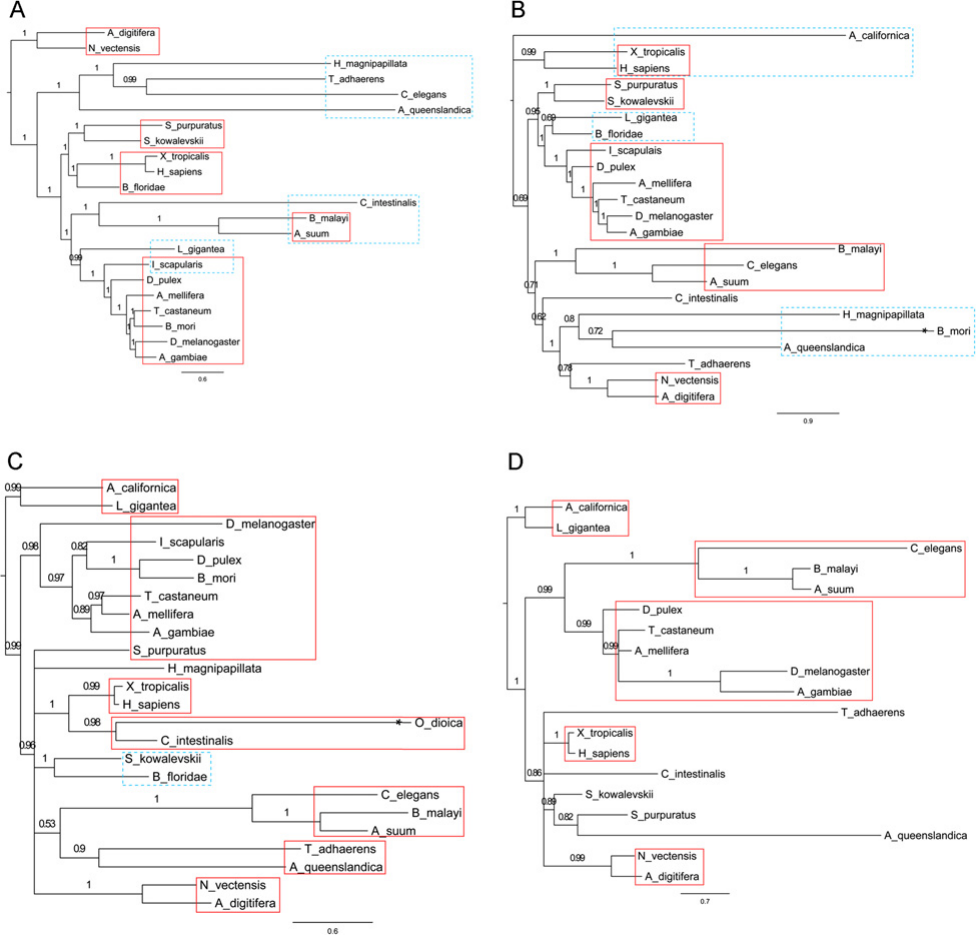
**The phylogenetic trees of *fat *(A), *dachsous *(B), *hippo *(C), and *yorkie *(D).** Columns with < =2 amino acids were removed in all datasets. Trees were produced by PhyloBayes (after 2000 generations, maxdiff = 0.01 for *fat*, 0.036 for *dachsous*, 0.05 for *hippo*, and 0.04 for *yorkie*) based on the substitution model LG + G (gamma category = 4). Trees are shown in scale, except branches with an asterisk. Numbers indicate posterior probability. Red frames mark species correctly grouped; blue frames mark species grouped as in the RAxML trees (Additional file 1: Figure S6).

PhyloBayes, RAxML, and PhyML produced trees without or with mild polytomies (Figure 6; Additional file 1: Figure S6; Additional file 1: Figure S7), but MrBayes produced the trees of *crumbs*, *dachsous*, and *fat* with a serious polytomy at the root, indicating that basic phyla cannot be resolved. To examine if the serious polytomy could be explained by divergence of sequences across phyla, we used MEGA to compute the overall mean distance (p-distance) of the seven genes [41] and found that the sequences of *crumbs*, *dachsous*, and *fat,* which are upstream regulators, have high overall mean p-distances (0.669, 0.676, 0.672) compared with the sequences of *hippo*, *merlin*, and *mats* (0.186, 0.369, 0.166), which are in the core of the Hippo pathway. We also examined the impact of gaps on tree building by removing the shortest *O. dioica* and columns with < =6 amino acids in the sequences of *crumbs*, and found that MrBayes produced a much better tree of *crumbs*. Thus, divergent species, potential recombination breakpoints, and gaps probably together influenced MrBayes’ Bayesian inference.

The phylogenetic trees of these genes show certain features. First, trees produced by PhyloBayes and by RAxML are highly consistent (Figure 6; Additional file 1: Figure S6), indicating robustness of tree building. Second, in some trees nodes at or near the root have lower posterior probability or bootstrap values compared with nodes near the leaves, indicating relatively unconvinced phyla and subphyla determination. This is in line with the serious polytomy at the root in the MrBayes trees of *crumbs*, *fat*, and *dachsous*. Third, in many trees the Porifera *A. queenslandica*, the Placozoa *T. adhaerens*, and the Hydrozoa *H. magnipapillata*, are misplaced, indicating drastic evolution of the Hippo pathway in early metazoans. The above two features may be due to too few species covering too many phyla in our datasets, or indicate significant phylum- or class-specific evolution of Hippo genes. Fourth, in most trees species within many classes, subphyla, or phyla (such as Anthozoa, Gastropoda, and Secernentea) are correctly determined. Finally, the polytomy in the Bayesian tree of *mats* (Additional file 1: Figure S7) may be caused by very short (226 aa) and highly conserved (the overall mean p-distance = 0.166) sequences. These features unveil some important aspects of the evolution of the Hippo pathway in metazoans.

### Evolution and Coevolution of genes in the Hippo pathway

**Figure 7:**
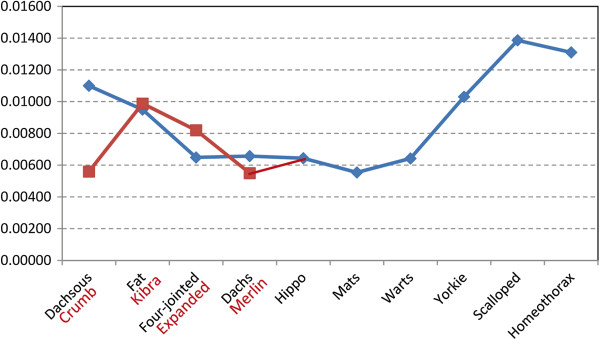
**dN/dS values of Hippo pathway genes across metazoan phyla.** The core Hippo pathway genes *hippo, mats*, and *warts* are evolutionarily more conserved than their upstream regulators and downstream partners. The Y axis indicates the dN/dS values.

As Hippo genes contain multiple functionally conserved domains, we examined their evolutionary conservation. When the random-site model in the PAML package was used to detect sites under positive and purifying selection [42], few or no sites in these genes were detected under positive selection at the 95% level (Additional file 1: Table S4) [43], but considerable sites in multiple genes were detected under positive selection at the 60-70% levels (the M2 model with naïve empirical Bayes analysis). We used the “Tests for alignment-wide evidence of selection” in the Datamonkey webserver to cross-check the data and obtained the same results. It appears that these results do not indicate significant false positively selected sites caused by recombination. We next examined whether genes at different positions in the Hippo pathway (upstream regulators, core Hippo complexes, the effector *yorkie*, and *yorkie*’s downstream partners) have been subjected to different selective forces. The values of ω = dN/dS indicate that *hippo*, *mats*, and *warts*, the core Hippo components at the center of the pathway, are most conserved (Figure 7). “Tests for alignment-wide evidence of selection” also confirmed that *dachs*, *merlin*, *hippo*, *mats*, and *warts* are the most conserved. In comparison, PCP-related upstream regulators and the transcriptional activators Scalloped and Homeothorax are more divergent (Figure 7). These results consist with the finding that sequences of Merlin, Mats, and Hippo have small, but sequences of Dachsous, Fat, and Yorkie have rather high, overall mean p-distances. The upstream regulators Dachsous and Fat and the downstream effector Yorkie, and its transcriptional partners, must have evolved to integrate multiple inputs and to activate different targets.

**Figure 8:**
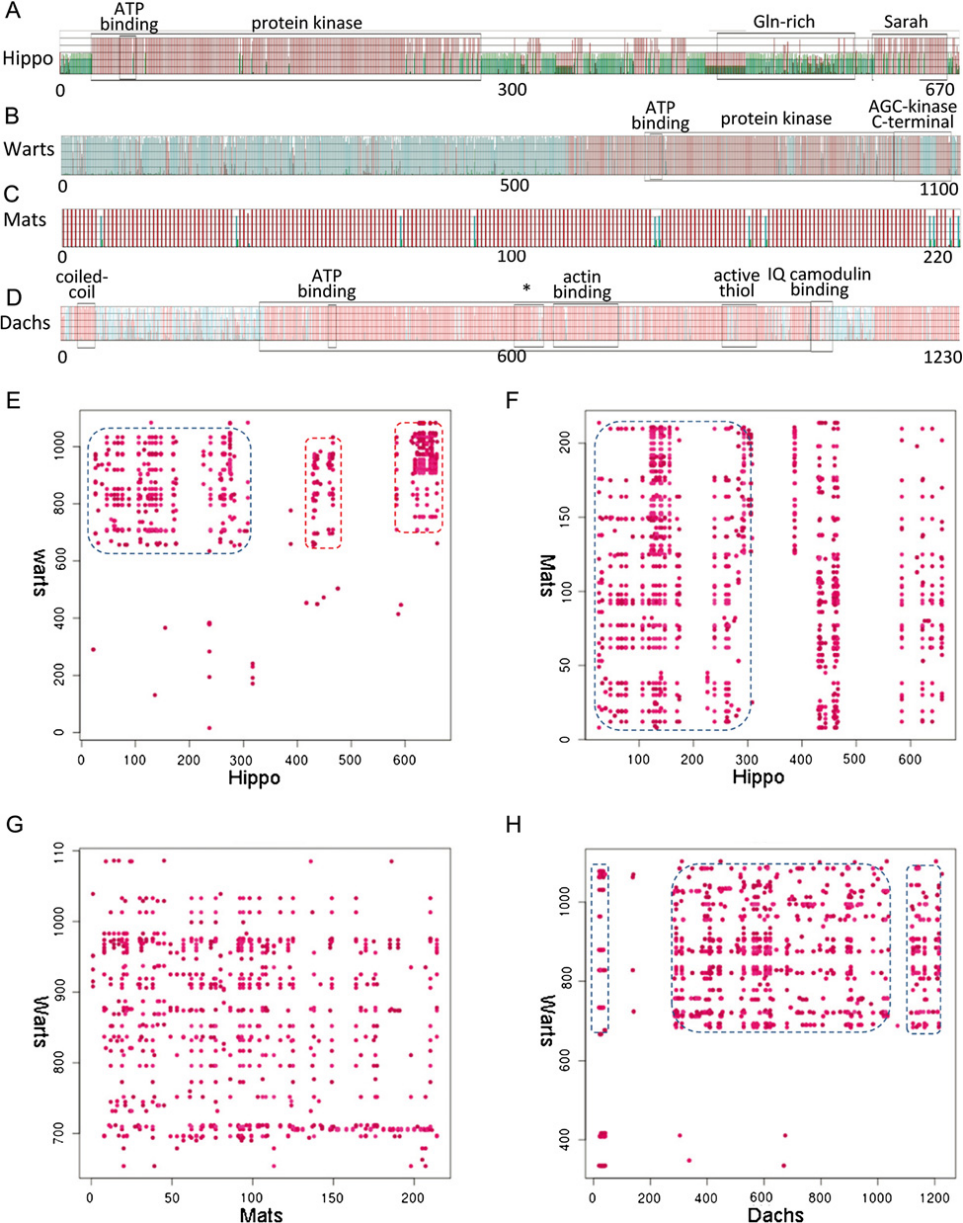
**Most functional domains in Hippo genes are under purifying selection and have coevolved with other functional domains.** (**ABCD**) are Drosophila Hippo, Warts, Mats and Dachs, with the number of amino acids marked below. Red, blue and green bars indicate sites under purifying selection, neutral evolution and positive selection, and black frames indicate identified functional domains. In (**D**) the symbol * indicates the “unique insert in myosin head domain” and the big frame from 274 aa to 1025 aa indicates “myosin head domain”. In **(EFGH)** red dots indicate coevolved sites in two genes. (**E**) Hippo’s 20–300 aa coevolve with Warts’s 600–900 aa (marked by the dashed black box); these two regions correspond to Hippo’s protein kinase domain and Warts’s protein kinase domain. Hippo’s 440–470 aa coevolve with Warts’s 600–900 aa; the former does not correspond to any identified domain in Hippo but the latter corresponds to the protein kinase domain. Also, Hippo’s 580–650 aa coevolve with Warts’s 640–1100 aa; these two regions largely correspond to Hippo’s Sarah domain and Warts’s AGC-kinase C-terminal domain. (**F**) Hippo’s 20–300 aa coevolve with the whole Mats (marked by the dashed black box), indicating that the whole Mats may be under purifying selection. (**G**) While coevolution signals occur evenly in the whole Mats, they exist only in Warts’s 700–1000 aa. This region in Warts corresponds to its protein kinase domain, which is under purifying selection. (**H**) Sites under purifying selection in Dachs (the coiled-coil domain, the myosin head domain, and the tail domain) coevolve with sites under purifying selection in Warts (the protein kinase domain). Notably, Dachs’s tail domain is under strong purifying selection and coevolves with Warts’s protein kinase domain (marked by the black boxes).

Physical interaction and functional association make proteins coevolved [44]. Typically, ligands and receptors should coevolve to maintain their physically interacting domains to fit for each other. One way to determine whether two genes or proteins have coevolved is to compute the pairwise distances between them across distant phyla and then the correlation between pairwise distances [45]. Using this distance matrix-based method, *yorkie* and *scalloped* were found to have coevolved [20]. A drawback of this method, however, is that it cannot detect which regions in the two proteins physically interact. To reveal coevolution of functional domains in Hippo pathway genes, we used the software CAPS to identify correlated pairwise amino acid variability for every pair of the 16 Hippo components [46]. Relatively high correlation coefficients (>0.6), if densely occurred at considerable sites, indicate potential coevolution of physically interacting or functionally associated regions in two proteins.

Upon identified functional domains, we first drew sites under positive selection (as mentioned above, at the 60-70% levels), neutral evolution and purifying selection. In the three core components Hippo, Warts and Mats, it is clear that all functional domains are under purifying selection and most regions under purifying selection are functional domains (Figure 8ABCD). This correspondence is in anticipation. Then, coevolutionary analysis reveals that many functional domains have coevolved with other functional domains (Figure 8EFGH), which allows one to infer whether a protein interacts with other proteins and how the interaction takes place. As an example, experimental studies indicate that Dachs coprecipitates and interacts with Warts [47], and our analysis reveals that Dachs may use its myosin head domain to bind to Warts’s protein kinase domain, because they share strong coevolution signals (Figure 8H). The combined use of the three analyses significantly helps identifying functional and interacting domains in Hippo genes.

Coevolution analysis also helps unveil why some domains have been lost in some species. For example, Dachs in some metazoans lost the coiled-coil domain and in some metazoans has a very short, or lost, IQ camodulin-binding domain. By evolutionary analysis we found that only half of the IQ camodulin-binding domain is under purifying selection, and by coevolutionary analysis we found that the coiled-coil domain poorly coevolves with any domain in Warts. These two findings provide a sensible explanation for the shortened or lost IQ camodulin-binding domain and the lost coiled-coil domain of Dachs in some species. Generally, applying evolutionary and coevolutionary analysis to identified functional domains revealed that most functional domains in Hippo genes have not only been under purifying selection but also coevolved with interacting functional domains in other genes. These results provide valuable clues for further examination of Hippo signaling.

## Discussion

Unlike other developmental pathways [5], the Hippo pathway seems to have evolved exclusively for the control of tissue, organ, and body size by regulating cell proliferation and apoptosis [9-11]. Due to its important roles in embryonic development, cancer, and tissue repair, the origin and evolution of the Hippo pathway has recently aroused immense interest. Previous investigations examined a few Hippo genes in limited metazoan phyla (especially, neglected Protostome groups such as mollusks), did not examine recombination breakpoints, and did not explore coevolution of functional domains [20,21]. In this study, our analysis of 16 Hippo genes in 24 metazoan phyla produced unprecedented details about functional domains and the evolution of the Hippo genes. First, the core Hippo components Mats/Hippo/Warts, their upstream regulators Fat/Dachsous/Crumbs, and the Yorkie partner Scalloped, originated first, indicating that these may comprise the kernel of the pathway, whereas other upstream regulators (Four-jointed), mediators of Hippo signaling (Salvador), and other Yorkie partners (Homeothorax) emerged later. Second, while most of these components are present in the most basal metazoan *A. queenslandica*, some seem to have been lost during Porifera and Placozoa to Cnidarian evolution. Third, in eumetazoans Hippo genes, especially *mats*, *hippo* and *warts*, are highly conserved, which is evidenced by the facts that genes have variable numbers of exons but maintain conserved numbers and organization of domains and genes have low overall mean p-distance. Fourth, there may be recombination breakpoints in some genes. Fifth, the two genes *fat* and *dachsous* important for both growth control and PCP may have inherited from the unicellular ancestor *M. Brevicollis* and have evolved significantly indicated by high overall mean p-distance. Finally, combined domain analysis, evolutionary analysis and coevolutionary analysis reveal that most functional domains in Hippo pathway genes have not only been under purifying selection but also coevolved with interacting functional domains in other genes.

Our analysis also shed light on how Hippo signaling and growth control were originated. Regulation of cell-cell contact and adhesion is important for epithelial patterning and required for the initiation of multicellularity. Although planar cell polarity (or aligned cell polarity) is thought to be a eumetazoan innovation that exists only in true epithelial cell layers [4] (but see also [48]), our analysis shows that the sponge *A. queenslandica* contains essential genes involved in both apicobasal cell polarity and planar cell polarity. Moreover, as cadherins mediating cell-cell contact and adhesion, Fat and Dachsous could originate quite early, probably in or before *M. brevicollis*. If these genes were inherited from unicellular ancestors, they should initially control cell contact and adhesion in unicellular organisms. An accidental event may have caused the genome containing these genes to produce permanently connected cells. For example, in sponges cadherins may allow cells to adhere to form tissue-like layers. Since transcriptionally active Yorkie and Scalloped were identified in the filasterean *C. owczarzaki*[21], the control of cell proliferation should be associated with cell-cell interactions and cellular patterning early in evolution. From unicellular to multicellular organisms, Fat and Dachsous, together with Yorkie and Scalloped, may have evolved to regulate both PCP and body size in Porifera and Placozoa. Later, the acquisition of more functional domains, upstream regulators and downstream targets makes the Hippo pathway to evolve to become one of the most conserved and essential developmental toolkits for tissue and organ size control in Bilateria.

Cell patterning and growth is intrinsically associated and the Fat-Dachsous interaction produces inputs for both the Hippo and PCP signaling [26]. In Drosophila, Fat and Dachsous regulate the atypical myosin Dachs to control polarized cell division [13]. Because the PCP mechanism is conserved in mammals [22,30], we expected to find orthologs of *dachs* in mammals. However, despite the existence of certain myosins such as PAR, which regulates cell polarization and movement in vertebrates [49], we failed to identify convincing ortholog of *dachs* in Chordates (Figure 2; Additional file 1: Figure S2). Whether other myosins replace Dachs’s role controlling polarized cell division in Chordates is an important issue awaiting further investigations.

Two ways allow genes encoding transcriptional factors to control tissue-specific gene expression. First, transcription factors can form various complexes to function in a tissue-specific manner in different contexts. Second, variable number of exons allow genes to produce, via alternative splicing, multiple proteins that function in different tissues and organs. Both seem to have been adopted by genes in the Hippo pathway. In basal metazoans that consist of just a few cell types, theoretically a large number of tissue-specific Yorkie transcriptional partners are not necessary. Nevertheless, we found that *hemeothorax*, a *yorkie* partner controlling cell division and apoptosis in the Drosophila eye, is present together with *scalloped* in both *A. queenslandica* and *T. adhaerens* (Figure 2). Since this gene contains just one exon in *A. queenslandica*, four exons in *T. adhaerens*, but many and highly varied numbers of exons (like *fat*) in other species (Additional file 1: Figure S3), we conclude that alternative splicing may be the most essential feature of Hippo pathway genes to produce multiple proteins for tissue-specific growth control.

## Conclusions

Some cadherins in the Hippo pathway may have originated in the unicellular organism *Monosiga brevicollis*. Compared with genes in other pathways, Hippo genes encode conserved and coevovled functional domains with varied numbers of exons in different species, and more exons in advanced organisms indicate significant alternative splicing to produce more tissue-specific transcripts. Phylogenetic analysis reveals that the upstream regulators and downstream targets have more significantly evolved than the core components, indicating that the Hippo pathway has integrated multiple developmental signals during evolution. After a few upstream regulators and core components forming the kernel of the Hippo pathway, some regulators have joined, but some others have been lost, in evolution. Annotated gene and domain sequences provide valuable clues for further examination of Hippo signaling.

## Methods

### Genes and species

The following 16 Hippo pathway genes were examined: *fat*, *dachsous*, *four-jointed*, *lowfat*, *dachs*, *hippo*, *salvador*, *warts*, *mats*, *yorkie*, *scalloped*, *kibra*, *expanded*, *merlin*, *homeothorax*, and *crumbs*. The examined metazoans included: *Drosophila melanogaster*, *Anopheles gambiae*, *Tribolium castaneum*, *Apis mellifera*, *Bombyx mori*, *Caenorhabditis elegans*, *Daphnia pulex*, *Saccoglossus kowalevskii*, *Lottia gigantea*, *Ascaris suum*, *Homo sapiens*, *Ciona intestinalis*, *Nematostella vectensis*, *Xenopus tropicalis*, *Acropora digitifera*, *Trichoplax adhaerens*, *Aplysia californica*, *Brugia malayi*, *Strongylocentrotus purpuratus*, *Amphimedon queenslandica*, *Branchiostoma floridae*, *Hydra magnipapillata*, *Ixodes scapularis*, and *Oikopleura dioica*.

### Identification of Hippo pathway orthologs

We obtained Hippo pathway gene and protein sequences for the above species from NCBI, Ensembl, OrthoDB, WormBase, and other databases (Additional file 1: Table S1). If a gene or protein for a given species was unavailable in these databases, we used BLASTP to search its available protein sequences in NCBI and used tBLASTN [50] to search through the exon sequences within the entire genome. Using exons and functional domains of annotated Hippo genes and proteins in *H. sapiens* and *D. melanogaster* as queries, we made at least four rounds of BLAST searches (in the third and fourth rounds of search, results produced by both of the two previous searches were used as new queries) for each unannotated gene. All BLAST hits were filtered, and only sequences with BLAST scores > 150 and E-values < 1E-5 were examined further. We also aligned BLAST hits to multiple query sequences and chose those with relative identity > 30% and relative similarity > 40% for further analysis.

If above searches failed to identify the orthologs of a gene (especially in genomes consisting of scaffolds), we used GeneWise [51] to examine the scaffolds that produced tBLASTN hits. For poorly conserved genes, we extended the 5’ and 3’ ends of each GeneWise output by 10,000 bp and used GenScan [52] to re-examine the outputs. To verify the obtained putative orthologous sequences, we used them as queries to search the NCBI database and determine whether they could successfully identify the homologous gene in *H. sapiens* and *D. melanogaster*. A putative ortholog was abandoned if an annotated gene was not identified by the reciprocal search. Sequences of putative orthologs predicted by GeneWise and GenScan are given in the (Additional file 2).

### Analysis of functional domains

We curated published articles (such as [53]) to determine the functional domains of Hippo pathway proteins. By searching the Conserved Domains Database (CDD) within NCBI, we determined the positions of functional domains in many genes. For poorly conserved or un-annotated domains that are not present within the NCBI CDD, we used annotated domains in some species as queries to search the sequences of genes/proteins of interest. The Jpred 3 Secondary Structure Prediction Server (http://www.compbio.dundee.ac.uk/www-jpred/) was used to analyze functional domains in different species.

### Recombination detection

We used six programs (RDP, GENECONV, MaxChi, BootScan, Chimaera, and SiScan) in RDP v3.44 to detect recombination in the 16 genes [35]. Default parameter values were adopted, except that the window side was 90 variable nucleotide positions (vnps) for the RPD, 400 vnps for the Bootscan, 140 nvps for the MaxChi, 120 vnps for the Chimaera, 200 vnps for the SiScan. Recombination events identified with a P-value < 0.01 and supported by at least three programs were reported. The protein sequences of seven genes – *merlin*, *mats*, *crumbs*, *fat*, *dachsous*, *hippo*, and *yorkie*, which have fewest recombination breakpoints, were chosen for phylogenetic analysis.

### Phylogenetic analysis

Codon sequences were aligned using ClustalW (codon) (with default parameters) and then translated into amino acid sequences in MEGA v5.1 [41]. Columns with < =2 amino acids were manually removed. The most appropriate substitution models for amino acid sequences were identified by ProtTest v3.4 (with default parameters) upon the Bayesian information criterion (in most cases the Akaike information criterion gave the same most appropriate model) [36]. The most appropriate substitution models for the *merlin* and *mats* datasets is LG + G, for the *crumbs* dataset is Blosum62 + G + I, and for *dachsous*, *fat*, *hippo*, and *yorkie* datasets is VT + G + I. PhyloBayes v3.3 (supports the LG model) [37], MrBayes v3.2 [39], PhyML v3.0 (using the T-REX webserver [54]) (supports the LG model) [38] and RAxML v7.2.8 [40] were used to build phylogenetic trees. Default parameters were adopted unless specifically mentioned. In running MrBayes, the average standard deviation < 0.01 was reached before the running was terminated. More details are given in Figure Legends.

### Evolution and coevolution analysis

We used MEGA v5.1 to compute the overall mean p-distances of each gene’s protein sequences [41]. We used the random-site model in the PAML package to perform likelihood-ratio tests of positive selection and to determine sites evolved under neutral, purifying and positive selections [42,43]. We also used “Tests for alignment-wide evidence of selection” in the Datamonkey webserver (http://www.datamonkey.org) to cross-check the results. We chose the ESD method (Genetic code = Universal code) to examine aligned gene sequences under the default parameter setting (Method = SLAC, nucleotide substitution bias model = REV, Global dN/dS value = Estimated, Handling ambiguities = Averaged, Significance Level = 0.1) [55]. Because CAPS can identify interacting regions in two proteins by computing the correlation in the pairwise amino acid variability [46], we applied CAPS to every pair of proteins encoded by the 16 genes to examine coevolution between amino acid sites.

## Competing interests

The authors declare that they have no competing interests.

## Authors’ contributions

Hao Zhu designed the research. Henan Zhu, ZZ, DW, WL and Hao Zhu performed the research. Hao Zhu wrote the manuscript with inputs from all co-authors. All authors read and approved the final manuscript.

## Supplementary Material

Additional file 1 Supplementary tables and figures. Click here for file

Additional file 2 Predicted gene sequences. Click here for file
