# Supplementary material for: Hippo pathway genes developed varied exon numbers and coevolved functional domains in metazoans for species specific growth control
Source: BMC Evol Biol. 2013 Apr 1;13:76. doi: 10.1186/1471-2148-13-76 (PMC3620549; doi:10.1186/1471-2148-13-76)
Supplement: Additional file 1 — Supplementary tables and figures. [file 1471-2148-13-76-S1.doc]

**Hippo pathway genes in metazoans developed varied exon numbers and**

**coevolved functional domains for species specific growth control**

Henan Zhu ^1^ Ziwei Zhou ^1^ Daxi Wang ^1^ Wenyin Liu ^1^ Hao Zhu ^1^*

Bioinformatics Section, School of Basic Medical Sciences

Southern Medical University, Guangzhou 510515, China

**Supplementary Table 1 The 16 Hippo pathway genes in the 24 species**

| **organism** | **yorkie** | **dachsous** | dachs | fat | four-jointed |
| --- | --- | --- | --- | --- | --- |
| Acropora  digitifera | predicted | predicted | none | predicted | none |
| Amphimedon queenslandica | predicted* | XP_003386184.1 | Ensembl:Transcript: PAC:15724244 | predicted | none |
| Anopheles gambiae | XP_556728.3 | XP_317578.4 | NCBI:XP_319057.4 | XP_317558.4 | XP_001237917.2 |
| Apis mellifera | XP_391844.3 | XP_392300.4 | NCBI:XP_624098.2 | XP_393497.4 | none |
| Aplysia californica | predicted | none | none | none | predicted |
| Ascaris suum | ADY44166.1 | ADY39852.1 | none | ADY39769.1 | none |
| Bombyx mori | NP_001116819 | NP_001037682.1 | Ensembl：BGIBMGA004656 | predicted | predicted |
| Branchiostoma floridae | XP_002595229 | XP_002611275.1 | JGI:e_gw.245.166.1 | XP_002587807.1 | XP_002609526.1 |
| Brugia malayi | XP_001893598.1 | XP_001900195.1 | none | XP_001900194.1 | none |
| Caenorhabditis elegans | NP_509789.2 | NP_497641.2 | none | NP_001021650.1 | none |
| Ciona intestinalis | XP_002130260 | XP_002120840.1 | none | XP_002124308.1 | ENSCINT00000021940 |
| Daphnia pulex | EFX70433.1 | EOG5JSXM9 | JGI：DappuT40739 | EFX69368.1 | EFX69885.1 |
| Drosophila melanogaster | NP_001036568.2 | NP_523446.2 | NCBI：AAK97502.3 | NP_477497.1 | NP_477483.1 |
| Homo sapiens | NP_001123617.1 | EAX04944.1 | none | NP_078858.4 | NP_055159.2 |
| Hydra magnipapillata | none | XP_002162352.1 | none | XP_002162352.1 | none |
| Ixodes scapularis | none | XP_002402241.1 | NCBI:XP_002404255.1 | XP_002405854.1 | XP_002433914.1 |
| Lottia gigantea | predicted | EOG5PG8Q8 | jgi|Lotgi1|215365| | jgi|105163|e_gw1.3.537.1 | jgi|pg.C_sca_1450012 |
| Nematostella vectensis | XP_001627495 | XP_001632533.1 | NCBI:XP_001634718.1 | predicted | none |
| Oikopleura dioica | none | none | none | none | none |
| Saccoglossus kowalevskii | XP_002734168 | XP_002735121.1 | predicted | XP_002731529.1 | XP_002736936.1 |
| Strongylocentrotus purpuratus | XP_789542.2 | XP_001177873.1 | Ensembl:SPU_013384gn | XP_785601.2 | XP_001175933.1 |
| Tribolium castaneum | XP_970492 | EFA02810.1 | NCBI:XP_969433.1 | XP_971084.2 | XP_973231.1 |
| Trichoplax adhaerens | XP_002108065.1 | XP_002112951.1 | NCBI：XP_002110197.1 | predicted | none |
| Xenopus tropicalis | NP_001182697.1 | XP_002933536.1 | none | XP_002932104.1 | XP_002937334.1 |

**Supplementary Table 1 The 16 Hippo pathway genes in the 24 species (continue)**

| **organism** | salvador | mats | hippo | lowfat | warts |
| --- | --- | --- | --- | --- | --- |
| Acropora digitifera | none | predicted | adi_v1.07695 | none | predicted |
| Amphimedon queenslandica | none | XP_003383130.1 | XP_003385366.1 | none | XP_003386265.1 |
| Anopheles gambiae | AGAP004628 | XP_320981.3 | AGAP006044 | XP_319761.3 | XP_313377 |
| Apis mellifera | GB13186 EOG56T1QF | XP_393046.2 | XP_393691.4 | XP_623659.3 | XP_395146.3 |
| Aplysia californica | none | predicted | predicted | none | predicted |
| Ascaris suum | ADY43420.1 | ADY48371.1 | ADY41880.1 | none | ADY41262.1 |
| Bombyx mori | BGIBMGA004937 | BGIBMGA013463-TA | BGIBMGA013478 | NP_001040253.1 | predicted |
| Branchiostoma floridae | estExt_fgenesh2_pg.C_80055 EOG54J589 | XP_002596178.1 | estExt_fgenesh2_pg.C_1840053 | XP_002605586.1 | XP_002587173.1 |
| Brugia malayi | XP_001897911.1 | none | XP_001897874.1 | none | XP_001897357.1 |
| Caenorhabditis elegans | predicted | NP_502248.2 | NP_508743.4 | none | NP_492699.1 |
| Ciona intestinalis | ENSCINP00000012865 EOG54J589 | ENSCING00000003276 | XP_002129172.1 | XP_002128590.1 | XP_002124396.1 |
| Daphnia pulex | scaffold_162:hxAUG25s162g243t1 | estExt_fgenesh1_pg.C_170329 | EFX80950.1 | EFX86835.1 | EFX78506.1 |
| Drosophila melanogaster | NP_788721.1 | NP_651041.3 | AAF57543.2 | NP_001188775.1 | NP_733403 |
| Homo sapiens | ENSP00000324729 EOG54J589 | ENSG00000114978 | NP_006272.2 | NP_714924.1 | NP_004681.1 |
| Hydra magnipapillata | none | XP_002157018.1 | XP_002166523.1 | none | XP_002154659.1 |
| Ixodes scapularis | none | ISCW013173-PA | ISCW021388 | XP_002406027.1 | XP_002405511.1 |
| Lottia gigantea | jgi|Lotgi1|189497| | jgi:estExt_fgenesh2_pg.C_sca_1820006 | gw1.70.119.1 | jgi|Lotgi1|180384 | predicted |
| Nematostella vectensis | NEMVEDRAFT_v1g238618-PA EOG54J589 | XP_001629887.1 | A7S0A9 | XP_001631685.1 | XP_001628097.1 |
| Oikopleura dioica | none | CBY24275.1 | CBY22488.1 | none | predicted |
| Saccoglossus kowalevskii | none | XP_002741420.1 | XP_002736706.1 | XP_002736441.1 | XP_002738496.1 |
| Strongylocentrotus purpuratus | SPU_024199tr EOG54J589 | XP_788775.1 | XP_781787.2 | XP_797503.1 | XP_795100.2 |
| Tribolium castaneum | TC009195 EOG56T1QF | XP_971775.1 | XP_975713.2 | XP_972203.1 | XP_973217.2 |
| Trichoplax adhaerens | TriadP57132 EOG54J589 | XP_002115400.1 | XP_002117150.1 | none | XP_002107701.1 |
| Xenopus tropicalis | ENSXETT00000057723 | fgenesh1_pg.C_scaffold_392000029 | ENSXETG00000017844 | XP_002944372.1 | XP_002943505.1 |

**Supplementary Table 1 The 16 Hippo pathway genes in the 24 species (continue)**

| organism | expanded | kibra | merlin | crumbs | scalloped |
| --- | --- | --- | --- | --- | --- |
| Acropora digictfera | none | predicted | predicted | none | predicted |
| Amphimedon queenslandica | none | none | predicted | XP_003387202.1 | XP_003388211.1 |
| Anopheles gambiae | XP_319884.4 | XP_311158.5 | XP_311595.3 | XP_310433.5 | XP_310580.4 |
| Apis mellifera | XP_393389.3 | XP_396884.3 | XP_003249526.1 | XP_001121416.2 | XP_392157.3 |
| Aplysia californica | predicted | predicted | predicted | none | predicted |
| Ascaris suum | ADY41185.1 | none | ADY47290.1 | ADY39807.1 | ADY45720.1 |
| Bombyx mori | none | predicted | predicted | BGIBMGA007609 | BGIBMGA001129 |
| Branchiostoma floridae | XP_002590857.1 | XP_002598149.1 | XP_002591890.1 | XP_002609244.1 | XP_002595807.1 |
| Brugia malayi | XP_001901669.1 | none | XP_001895150.1 | none | XP_001893493.1 |
| Caenorhabditis elegans | NP_506085.2 | none | NP_001022571.1 | NP_510822.1 | NP_871906.2 |
| Ciona intestinalis | none | XP_002129513.1 | XP_002125990.1 | XP_002124076.1 | NP_001071810.1 |
| Daphnia pulex | EFX80952.1 | EFX86200.1 | EFX76345.1 | EFX83458.1 | EFX89756.1 |
| Drosophila melanogaster | NP_476840.2 | NP_001034055 | NP_523413.1 | AAA28428.1 | AAA28881.1 |
| Homo sapiens | NP_001035946.1 | NP_001155133.1 | NP_000259.1 | AAF01361.1 | NP_068780.2 |
| Hydra magnipapillata | none | XP_002163573.1 | XP_002160704.1 | none | XP_002160172.1 |
| Ixodes scapularis | none | XP_002408258.1 | XP_002406151.1 | none | XP_002409069.1 |
| Lottia gigantea | predicted | predicted | predicted | jgi|Lotgi1|238278 | predicted |
| Nematostella vectensis | none | XP_001629271.1 | XP_001636182.1 | none | XP_001628874.1 |
| Oikopleura dioica | none | none | CBY09777.1 | XP_001913205.1 | CBY14221.1 |
| Saccoglossus kowalevskii | XP_002731310.1 | XP_002738693.1 | NP_001164711.1 | XP_002735391.1 | NP_001161650.1 |
| Strongylocentrotus purpuratus | XP_797238.2 | XP_780710.2 | XP_781142.2 | XP_001179074.1 | XP_785374.3 |
| Tribolium castaneum | XP_970685.1 | XP_974791.2 | XP_972226.2 | XP_970640.1 | ACN43339.1 |
| Trichoplax adhaerens | none | none | none | XP_002113807.1 | XP_002110587.1 |
| Xenopus tropicalis | NP_001016027.1 | NP_001096212.1 | NP_001072362.1 | XP_002937280.1 | XP_002943057.1 |

**Supplementary Table 1 The 16 Hippo pathway genes in the 24 species (continue)**

| organism | homeothorax |
| --- | --- |
| Acropora digictfera | predict |
| Amphimedon queenslandica | PAC:15726440 |
| Anopheles gambiae | AGAP002178-RA |
| Apis mellifera | GB13662-RA |
| Aplysia californica | predict |
| Ascaris suum | ADY43680.1 |
| Bombyx mori | none |
| Branchiostoma floridae | estExt_fgenesh2_pm.C_1200015 |
| Brugia malayi | EDP28805.1 |
| Caenorhabditis elegans | NP_001024173.1 |
| Ciona intestinalis | ENSCINT00000011017 |
| Daphnia pulex | none |
| Drosophila melanogaster | FBgn0001235 |
| Homo sapiens | ENST00000272369 |
| Hydra magnipapillata | LOC100215603 |
| Ixodes scapularis | none |
| Lottia gigantea | e_gw1.37.126.1 |
| Nematostella vectensis | NEMVEDRAFT_v1g119733-RA |
| Oikopleura dioica | predict |
| Saccoglossus kowalevskii | predict |
| Strongylocentrotus purpuratus | SPU_011202tr |
| Tribolium castaneum | TCOGS2:TC008629-RA |
| Trichoplax adhaerens | TriadT59844 |
| Xenopus tropicalis | ENSXETT00000025054 |

**Supplementary Table 2 Comparison of yorkie orthologs with yorkie in Drosophila**

| **yorkie orthologs in species** |  |  | **Compared with yorkie in Drosophila melanogaster** | | |
| --- | --- | --- | --- | --- | --- |
|  | **Sources** | **ID** | **Score** | **relative identity** | **relative similarity** |
| *Drosophila melanogaster* | NCBI | NP_001036568.2 | - | - | - |
| *Anopheles gambiae* | NCBI | XP_556728.3 | 530 | 0.29 | 0.411 |
| *Tribolium castaneum* | NCBI | XP_970492 | 553 | 0.287 | 0.386 |
| *Apis mellifera* | NCBI | XP_391844.3 | 570 | 0.268 | 0.363 |
| *Bombyx mori* | NCBI | NP_001116819 | 546 | 0.273 | 0.347 |
| *Caenorhabditis elegans* | NCBI | NP_509789.2 | 149.5 | 0.181 | 0.321 |
| *Daphnia pulex* | NCBI | EFX70433.1 | 472 | 0.24 | 0.305 |
| *Saccoglossus kowalevskii* | NCBI | XP_002734168 | 425 | 0.215 | 0.283 |
| *Lottia gigantea* | predicted |  | 344 | 0.184 | 0.276 |
| *Ascaris suum* | NCBI | ADY44166.1 | 253.5 | 0.17 | 0.269 |
| *Homo sapiens* | NCBI | NP_001123617.1 | 390.5 | 0.178 | 0.265 |
| *Ciona intestinalis* | NCBI | XP_002130260 | 390 | 0.188 | 0.263 |
| *Nematostella vectensis* | NCBI | XP_001627495 | 317.5 | 0.19 | 0.263 |
| *Xenopus tropicalis* | NCBI | NP_001182697.1 | 411 | 0.193 | 0.261 |
| *Acropora digitifera* | predicted |  | 357 | 0.18 | 0.257 |
| *Trichoplax adhaerens* | NCBI | XP_002108065.1 | 216.5 | 0.167 | 0.256 |
| *Aplysia californica* | predicted |  | 348 | 0.186 | 0.246 |
| *Brugia malayi* | NCBI | XP_001893598.1 | 185 | 0.132 | 0.241 |
| *Strongylocentrotus purpuratus* | NCBI | XP_789542.2 | 407.5 | 0.182 | 0.24 |
| *Amphimedon queenslandica* | Ensembl | Aqu1.229695 | 174.5 | 0.152 | 0.229 |
| *Branchiostoma floridae* | NCBI | XP_002595229 | 236.5 | 0.118 | 0.144 |
| *Hydra magnipapillata* | none |  |  |  |  |
| *Ixodes scapularis* | none |  |  |  |  |
| *Oikopleura dioica* | none |  |  |  |  |

**Supplementary Table 3 Comparison of yorkie orthologs with Yap in human**

| **yorkie orthologs in species** |  |  | **Compared with Yap in Homo sapiens** | | |
| --- | --- | --- | --- | --- | --- |
|  | **Sources** | **ID** | **Score** | **relative identity** | **relative similarity** |
| Homo sapiens | NCBI | NP_001123617.1 | - | - | - |
| Xenopus tropicalis | NCBI | NP_001182697.1 | 1999 | 0.773 | 0.83 |
| Saccoglossus kowalevskii | NCBI | XP_002734168 | 986.5 | 0.424 | 0.545 |
| Strongylocentrotus purpuratus | NCBI | XP_789542.2 | 780 | 0.33 | 0.475 |
| Nematostella vectensis | NCBI | XP_001627495 | 645 | 0.333 | 0.47 |
| Lottia gigantea | **predicted** |  | 693 | 0.305 | 0.455 |
| Acropora digitifera | **predicted** |  | 628.5 | 0.326 | 0.455 |
| Apis mellifera | NCBI | XP_391844.3 | 718 | 0.336 | 0.445 |
| Bombyx mori | NCBI | NP_001116819 | 667 | 0.323 | 0.445 |
| Aplysia californica | **predicted** |  | 706 | 0.319 | 0.443 |
| Tribolium castaneum | NCBI | XP_970492 | 684 | 0.323 | 0.438 |
| Daphnia pulex | NCBI | EFX70433.1 | 710 | 0.32 | 0.43 |
| Ciona intestinalis | NCBI | XP_002130260 | 653.5 | 0.279 | 0.415 |
| Trichoplax adhaerens | NCBI | XP_002108065.1 | 301 | 0.23 | 0.347 |
| Anopheles gambiae | NCBI | XP_556728.3 | 479.5 | 0.253 | 0.344 |
| Brugia malayi | NCBI | XP_001893598.1 | 244.5 | 0.204 | 0.32 |
| Ascaris suum | NCBI | ADY44166.1 | 303.5 | 0.209 | 0.313 |
| Amphimedon queenslandica | Ensembl | Aqu1.229695 | 197.5 | 0.2 | 0.294 |
| Caenorhabditis elegans | NCBI | NP_509789.2 | 223.5 | 0.184 | 0.275 |
| Drosophila melanogaster | NCBI | NP_001036568.2 | 390.5 | 0.178 | 0.265 |
| Branchiostoma floridae | NCBI | XP_002595229 | 278 | 0.105 | 0.131 |
| Hydra magnipapillata | none |  |  |  |  |
| Ixodes scapularis | none |  |  |  |  |
| Oikopleura dioica | none |  |  |  |  |

Supplementary Table 4 Conservation of some Hippo genes

| gene | model | Estimates of parameters | lnL | LRT | Positively selected sites # |
| --- | --- | --- | --- | --- | --- |
| dachs | M1 | kappa (ts/tv) = 1.49344 | -9788.38721 |  | none |
|  |  | p: 0.75963 0.24037 |  |  |  |
|  |  | w: 0.01075 1.00000 |  |  |  |
|  | M2 | kappa (ts/tv) = 1.49343 | -46139.11568 | Accepted(99%) | none |
|  |  | p: 0.75963 0.09006 0.15030 |  |  |  |
|  |  | w: 0.01075 1.00000 1.00000 |  |  |  |
|  | M7 | kappa (ts/tv) = 1.44682 | -45555.84778 |  | none |
|  |  | p=0.85594 q=38.00828 |  |  |  |
|  | M8 | kappa (ts/tv) = 1.47669 |  |  | none |
|  |  | p0=0.86202 p=1.10067 q=55.68462 | -45512.09507 | Rejected |  |
|  |  | (p1=0.13798) w=1.00000 |  |  |  |
| yorkie | M1 | kappa (ts/tv) = 1.27545 | -29816.9664 |  | none |
|  |  | p: 0.64342 0.35658 |  |  |  |
|  |  | w: 0.07735 1.00000 |  |  |  |
|  | M2 | kappa (ts/tv) = 1.27545 | -29816.9664 | Accepted(99%) | none |
|  |  | p: 0.64342 0.17368 0.18290 |  |  |  |
|  |  | w: 0.07735 1.00000 1.00000 |  |  |  |
|  | M7 | kappa (ts/tv) = 1.13321 | -29445.99077 |  | none |
|  |  | p=1.18137 q=21.42461 |  |  |  |
|  | M8 | kappa (ts/tv) = 1.13514 | -29445.57052 | Accepted(99%) | none |
|  |  | p0=0.98757 p=1.20790 q=22.39630 |  |  |  |
|  |  | (p1=0.01243) w=1.00000 |  |  |  |
| mats | M1 | kappa (ts/tv) = 1.39406 | -9788.38721 |  | none |
|  |  | p: 0.85901 0.14099 |  |  |  |
|  |  | w: 0.01107 1.00000 |  |  |  |
|  | M2 | kappa (ts/tv) = 1.39406 | -9788.38721 | Accepted(99%) | none |
|  |  | p: 0.85901 0.11483 0.02616 |  |  |  |
|  |  | w: 0.01107 1.00000 1.00000 |  |  |  |
|  | M7 | kappa (ts/tv) = 1.14983 | -9465.680217 |  | none |
|  |  | p=0.40474 q=15.99178 |  |  |  |
|  | M8 | kappa (ts/tv) = 1.14984 | -9465.680458 | Accepted(99%) | none |
|  |  | p0=0.99999 p=0.40475 q=15.99243 |  |  |  |
|  |  | (p1=0.00001) w=1.00000 |  |  |  |
| hippo | M1 | kappa (ts/tv) = 1.30869 | -28628.56306 |  | none |
|  |  | p: 0.60128 0.39872 |  |  |  |
|  |  | w: 0.04495 1.00000 |  |  |  |
|  | M2 | kappa (ts/tv) = 1.30869 | -28628.56306 | Accepted(99%) | none |
|  |  | p: 0.60127 0.16941 0.22932 |  |  |  |
|  |  | w: 0.04495 1.00000 1.00000 |  |  |  |
|  | M7 | kappa (ts/tv) = 1.10677 | -27755.77033 |  | none |
|  |  | p=0.41128 q=10.05119 |  |  |  |
|  | M8 | kappa (ts/tv) = 1.10677 | -27755.7709 | Accepted(99%) | none |
|  |  | p0=0.99999 p=0.41129 q= 10.05169 |  |  |  |
|  |  | (p1=0.00001) w=1.00000 |  |  |  |
| hth | M1 | kappa (ts/tv) = 1.46889 | -25071.0293 |  | none |
|  |  | p: 0.46379 0.53621 |  |  |  |
|  |  | w: 0.07561 1.00000 |  |  |  |
|  | M2 | kappa (ts/tv) = 1.46889 | -25071.0293 | Accepted(99%) | none |
|  |  | p: 0.46379 0.33534 0.20087 |  |  |  |
|  |  | w: 0.07561 1.00000 1.00000 |  |  |  |
|  | M7 | kappa (ts/tv) = 1.15766 | -24590.89854 |  | none |
|  |  | p=0.89367 q=6.32614 |  |  |  |
|  | M8 | kappa (ts/tv) = 1.15967 | -24589.30157 | Rejected | none |
|  |  | p0=0.98529 p=0.91991 q=7.13381 |  |  |  |
|  |  | (p1=0.01471) w=2.29072 |  |  |  |

# Upon BEB (Bayes Empirical Bayes analysis) in PAML. Kappa is estimated.

-
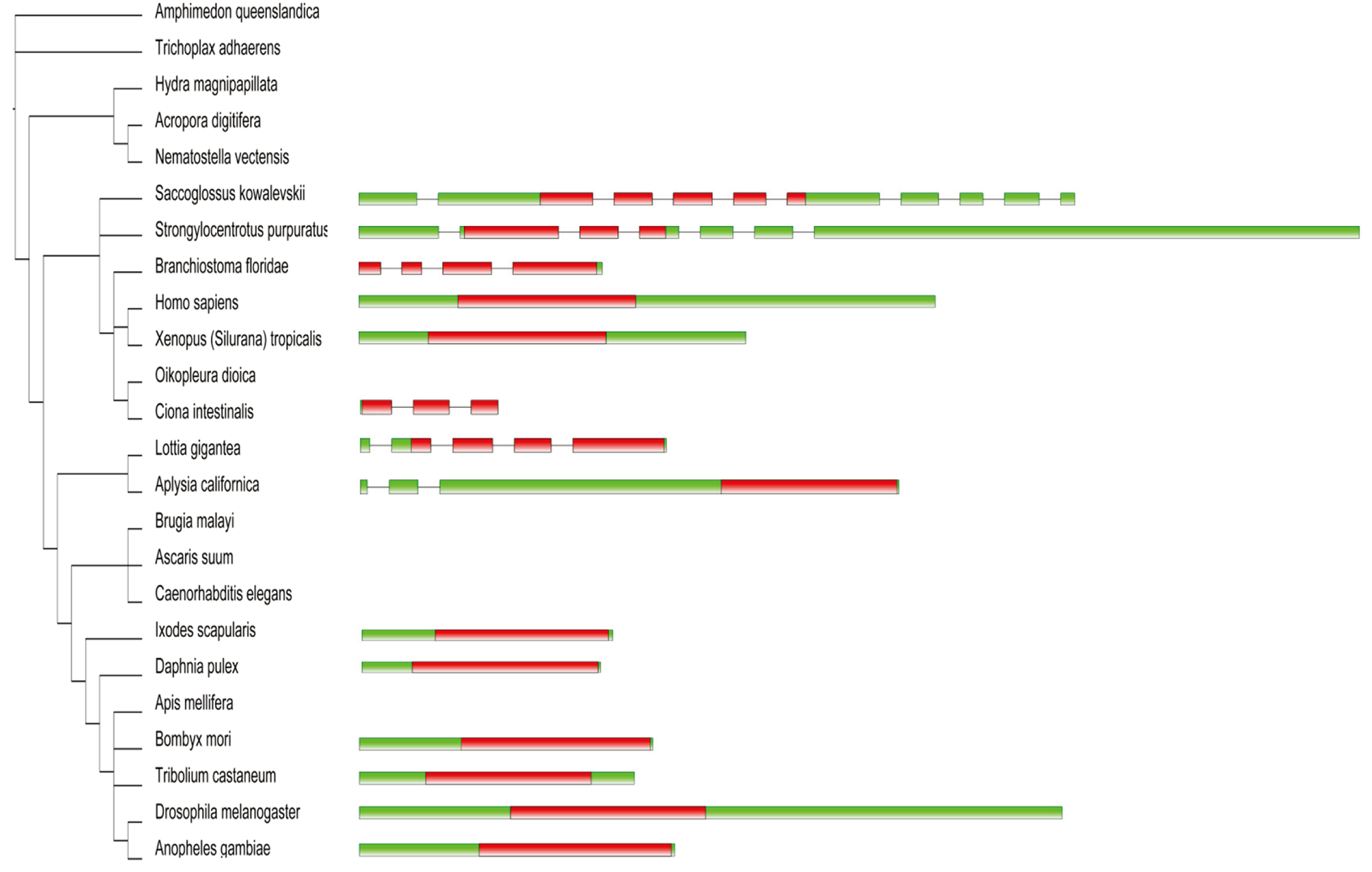


**Supplementary Figure 1 Exons and functional domains of *four-jointed* in metazoans.** Exons are indicated as boxes and shown in green, and red indicates the FAM20_C_like domain.


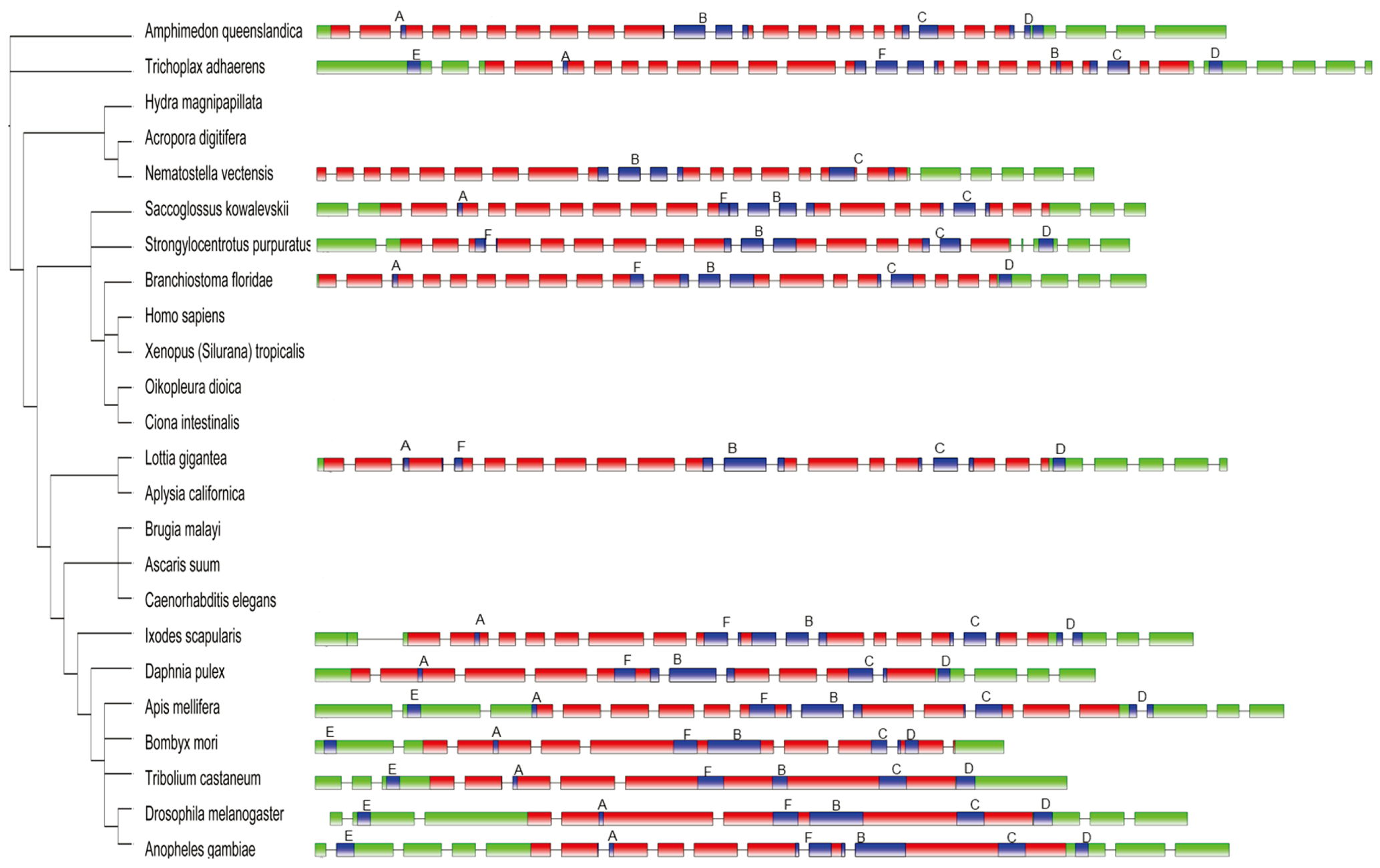


**Supplementary Figure 2 Exons and functional domains of *dachs* in metrazoans.** Exons are indicated as boxes and shown in green, and functional domains are indicated by colors and letters (note that S. purpuratus has a very short exon before the IQ camodulin-binding domain). Red indicates the HEAD domain and blue indicates domains within. Letters indicate: A─the ATP-binding domain, B─the actin-binding domain; C─the active thiol domain, D─the IQ camodulin-binding domain, E─the coiled-coil domain, F─a unique insert.


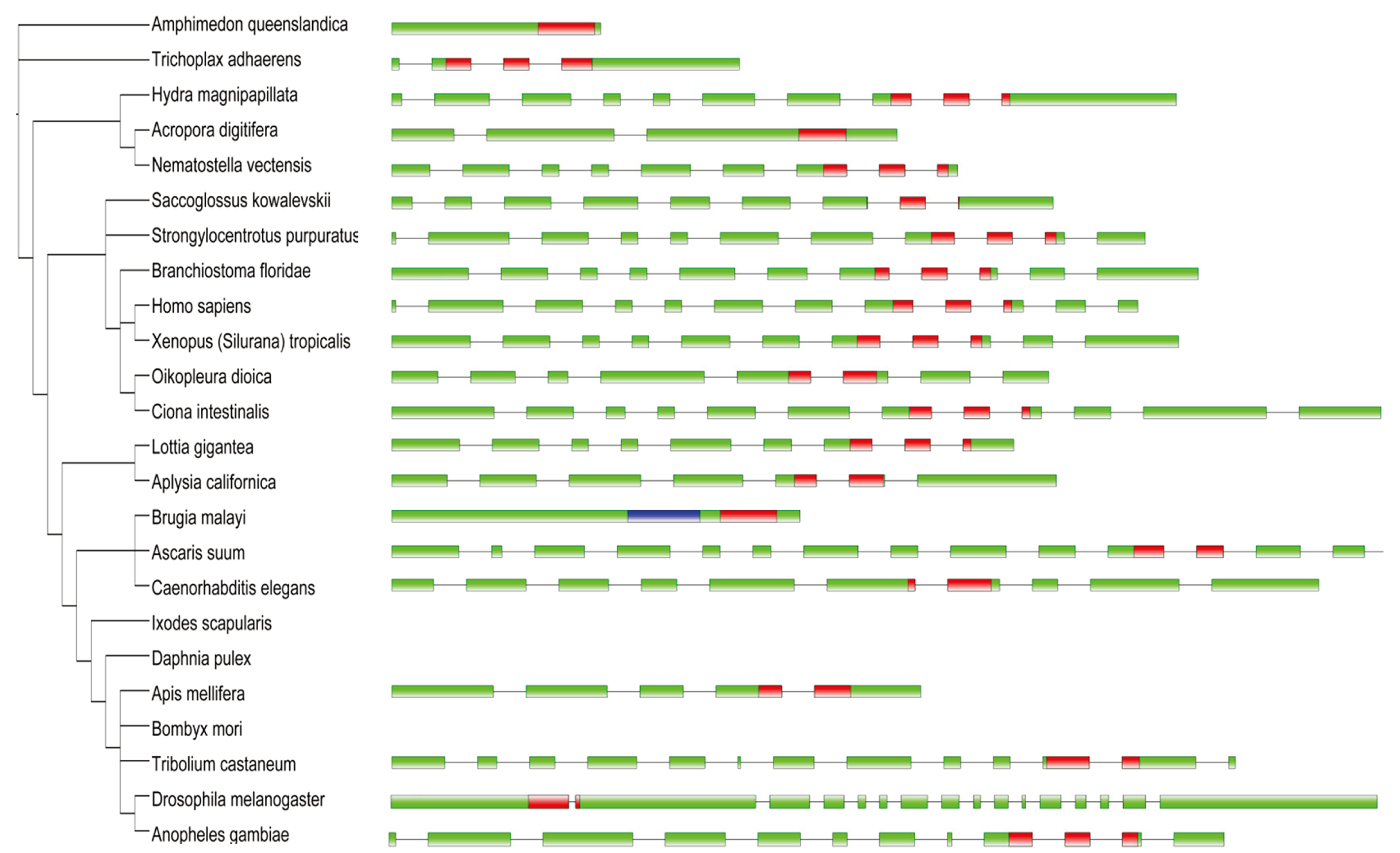


**Supplementary Figure 3 Exons and functional domains of *homeothorax* (hth) in metazoans.** Exons are indicated as boxes and shown in green, and functional domains are shown in other colors. Red indicates the homeobox domain and green indicates the POU domain.

H.sapiensTEAD2 --EEEEEE--EEEE-----------EEEEEE--------------HHHHHHHHH-------HHHHHH------EEEEEEEEE--------------------EEEEEEEE-----EEEEEEEEHHH---HHHHHEEEE-------EEEEEE-----HHHHHHHHHHHH----HHHHHHHHH--EEEEEEE------EEEEEEEEEEE--------EEEEEE---

A.queenslandica --------------EEEEEEEE-------------EEEEE--------------HHHHHHHH-------HHHHHH------EEEEEEEE------------EEEEEE-------EEEEEEEEHHH-----EEEE----------EEEEEE----HHHHHHHHHHHHH---HHHHHHHHH---EEEEEEE------EEEEEEEEEEE--------EEEEEEE---

T.adhaerens -------HHHH---EEEEEEEEE------------EEEE------------EEEHHHHH------HHHHHHHH------EEEEEEE------------EEEEEEE------EEEEEEEEHHH-----EEEE----------EEEEEE----HHHHHHHHHHHHH---HHHHHHHHH---EEEEEEE------EEEEEEEEEEE--------EEEEEE-------

H.magnipapillata -----EEEE-------------------EEEEEE-------------HHHHHHHHHH------HHHHHH------EEEEEEEE------------EEEEEEE------EEEEEEEEEHHH-HHHHHHHHH---------EEEEE-----HHHHHHHHHHHH---HHHHHHHHHH--EEEEEEE------EEHHHHHHEEH--------EEEEEE----------

A.digitifera ---------------EEEE----EEE-EEEEE--------------------------HHHHHHHHHHHHHHHHHHH-----HHHHEEEE--------------EEEE---EEEEEEEEEE-------EEEEEE-----HHHHHHHHHHHH----HHHHHHH----HHHHHHHHHHHHH---------------------------------------------

N.vectensis --------------EEEE------------------EEEEE---------------HHHHHH-------HH-----------EEEEE-----------------------------EEEEEEEEE---HHHHHHH----------EEEEEE----HHHHHHHHHHHHH----HHHHHHHHH--EEEEEEE------EEEEEEEEEEE--------EEEEEE---

S.kowalevskii -------------EEEEEEEEEEEE----------EEEEEE-------------HHHHHHHHHH------HHHHHH------EEEEEEEE------------EEEEEE-------EEEEEEEEHH-----EEEEEE---------EEEEEE----HHHHHHHHHHHHH----HHHHHHHH---EEEEEEE------EEEEEEEEEEE--------EEEEEE---

S.purpuratus -------------EEEEEEEEEEEE-----------EEEEE-------------HHHHHHHHHH------HHHHHH------EEEEEEEE------------EEEEEEE------EEEEEEEEHHH-----EEEE----------EEEEEE----HHHHHHHHHHHHH---HHHHHHHHH---EEEEEEE------EEEEEEEEEEE--------EEEEEEE--

B.floridae ------------EEEEEEEEEE-------------EEEEE--------------HHHHHHHHHH------HHHHHH------EEEEEEEE------------EEEEE--------EEEEEEEEHHH-----EEEE----------EEEEEE----HHHHHHHHHHHHH---HHHHHHHHH---EEEEEEE------EEEEEEEEEEE--------EEEEEEE--

H.sapiens ------------EEEEEEEEEEEE-----------EEEEE--------------HHHHHHHHHH------HHHHHH------EEEEEEEE------------EEEEEEE------EEEEEEEEHHH-----EEEE----------EEEEEE----HHHHHHHHHHHHH---HHHHHHHHH---EEEEEEE------EEEEEEEEEEE--------EEEEEEE--

X.tropicalis ------------EEEEEEEEEE-------------EEEEE--------------HHHHHHHHHH------HHHHHH------EEEEEEEE------------EEEEEEE------EEEEEEE--HH---HHHHHHHH--------EEEEEE-----HHHHHHHHHHHH---HHHHHHHHH---EEEEEEE------HEEEEEEEEEE--------EEEEEE---

O.dioica ---EEEEE----------------------HHHEEE---------------HHHHHHHHH-------HHHHHHH-------EEEEEEEE------------EEEEEEEE------EEEEEEEEHHHHHHHHHHH----------EEEEEE-HHHHHHHHHHHHHHH-----HHHHHHHH---EEEEEEE-----EEHHHHEEEEEEE-------EEEEEE----

C.intestinalis -----------EEEEEEEEEEEE-----------EEEEEE-------------HHHHHHHHHHH------HHHHHH------EEEEEE--------------EEEEEEEE-----EEEEEEEEHHH----HHHHHHH--------EEEEEE----HHHHHHHHHHHHH----HHHHHHH----EEEEEEE------EEEEEEEEEEE--------EEEEEE---

L.gigantea ---------------EEEEEEEEE-----------EEEEEE-------------HHHHHHHHH-----HHHHHHHH------EEEEEEE-------------EEEEE--------EEEEEEEEHHHH-HHHHHHH----------EEEEEE----HHHHHHHHHHHHH---HHHHHHHHH---EEEEEEE------EEEEEEEEEEEE-------EEEEEE---

A.californica -------------EEEEE--EEEE-----------EEEEEE--------------HHHHHHHHH------HHHHHH------EEEEEEEE-----E------EEEEEEE------EEEEEEEEHHH-----EEEE----------EEEEEE----HHHHHHHHHHHHH---HHHHHHHHH---EEEEEEE------EEEEEEEEEEE--------EEEEEE---

B.malayi ----------EEEEEEEEEEE---------EEEEEE-----------HHHHHHHHHHHHHHHHHHH------EEEEEEE---EEEE------EEEEEE-------EEEEEEEE-------HEEE----------------------------------------------------------------------------------------------------

A.suum ---------------EEEEEEEEEE---------EEEEEE------------HHHHHHHHHHHHHHHHHH-------EEEEEEE-EEEEE-------EEEE--------EEEEEEEEHHH-----EEEEEE---EE---EEEEEE----HHHHHHHHHHHHH----HHHHHHHH---EEEEEEE------EEEEEEEEEEEE------EEEEEE----------

C.elegans -------------------------------------EEEEEE---------HHHHHHHHHHHHHHHHHHH-------EEEEEEE-------------EEEEEE------EEEEEEEHHHHH-HHHHHHHH---------EEEEEE----HHHHHHHHHHHHH---HHHHHHHHH----EEEEEE------EEEEEEEEEEE-------EEEEEEE--------

I.scapularis -----EEEEEE--EEEEE----------EEEEEE-------------HHHHHHHHHH------HHHHHH------EEEEEEEE------------EEEEEEE------EEEEEEEEHHH----HHHHHH---------EEEEEE----HHHHHHHHHHHHH----HHHHHHHHHHHEEEE-----EEEE------EEEEEEEEEEE--------EEEEEE---

D.pulex --------------EEEE------------------EEEE--------------HHHHHHHHHH------HHHHHH------EEEEEEEEE-----------EEEEEEEE-----EEEEEEEHHHH----HHHEE----------EEEEEE----HHHHHHHHHHHHH---HHHHHHHHH---EEEEEEE------EEEEEEEEEEE--------EEEEEE---

A.mellifera -------------EEEEEEEEEE------------EEEEEE-------------HHHHHHHHHH------HHHHHH------EEEEEEEE-EEEE-------EEEEEEEE-----EEEEEEEEHHH-----EEEE----------EEEEEE----HHHHHHHHHHHHH---HHHHHHHHH---EEEEEEE------EEEEEEEEEEE--------EEEEEE---

B.mori -----EE----EEEEEEEEE----------HHEEEEE--------------HHHHHHHHHHHHH-----HHHHHHHHH---HHHHHHHHHHHHH------------------EEEEEE-----EEEEEEEEHH----------EEEEEE----------EEE-------EEEEEHH-------EEEEE-------HHHHHHH----HHHHHHHHHHHHHHHHHH

T.castaneum -----------EEEEE--EEE-----------EEEEEE-------------------HHH--------HHHHH-------EEEEEE---------------EEEE--------EEEEEEEEEE---HHHHHHH----------EEEEEE----HHHHHHHHHHHHH----------------------------------------------------------

D.melanogaster -------HHHH-EEEE---EEE------------EEEEE---------------HHHHHH-------HHHHHHH-------EEEEEEE--------------EEEEE--------EEEEEEEEHH------EEEE----------EEEEEE----HHHHHHHHHHHHH---HHHHHHHHH---EEEEEEE------EEEEEEEEEEE--------EEEEEE---

A.gambiae ------------------EEEEEEEE----------EEEEEE--------------HHHHHHH-------HHHHHH------EEEEEEEE------------EEEEE--------EEEEEEEEHHH----HHHHH----------EEEEEE----HHHHHHHHHHHHH----HHHHHHHHH--EEEEEEE------EEEEEEEEEEE--------EEEEEE---

**Supplementary Figure 4 Secondary structures of Hippo proteins. E indicates beta sheet and H indicates helix.**


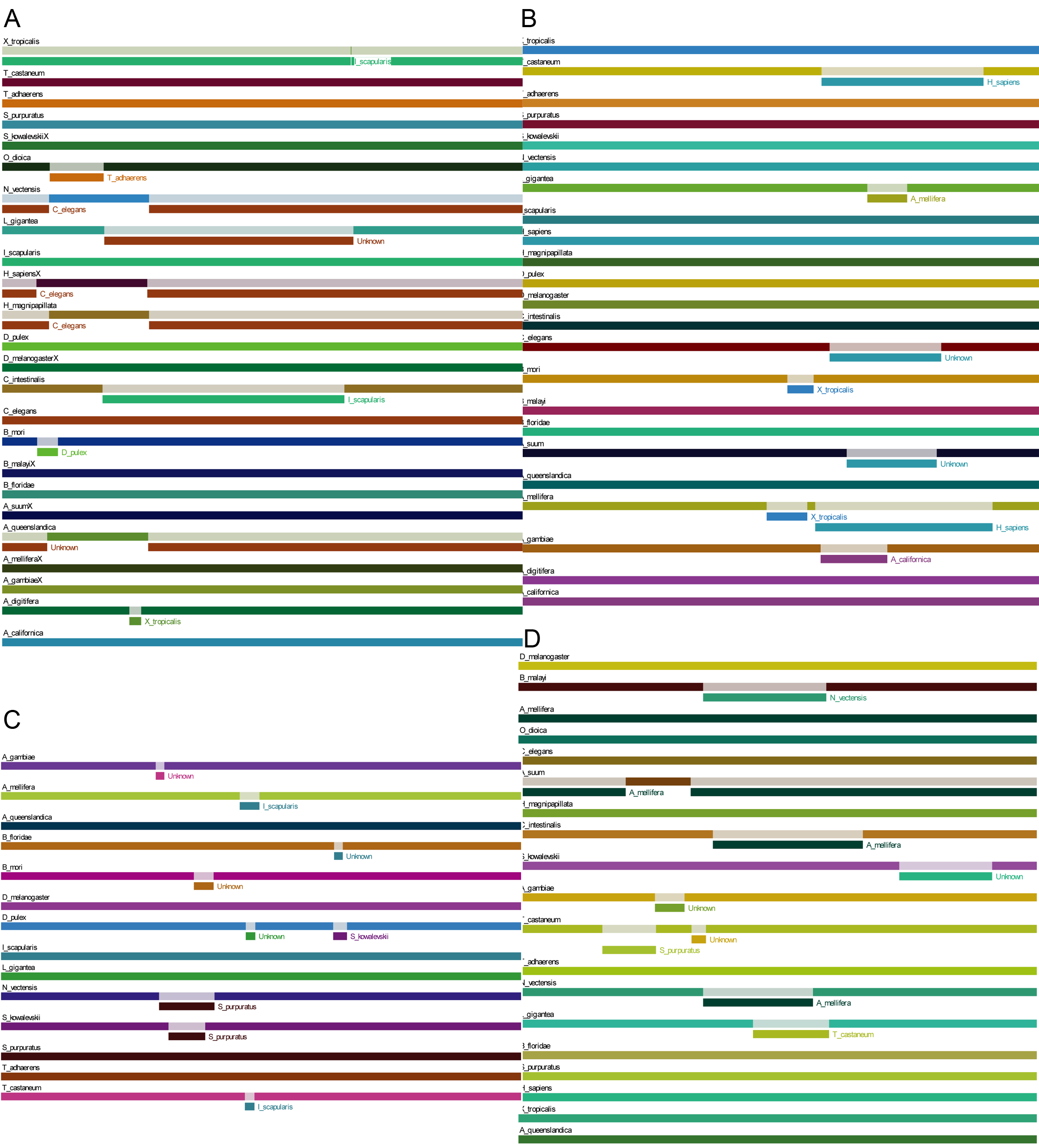


**Supplementary Figure 5 Detected recombination breakpoints in *scalloped* (A), *warts* (B), *dachs* (C) and *homeothorax* (D).** Recombination breakpoints were detected by >=3 programs in RDP v3.44 (see Methods).

**
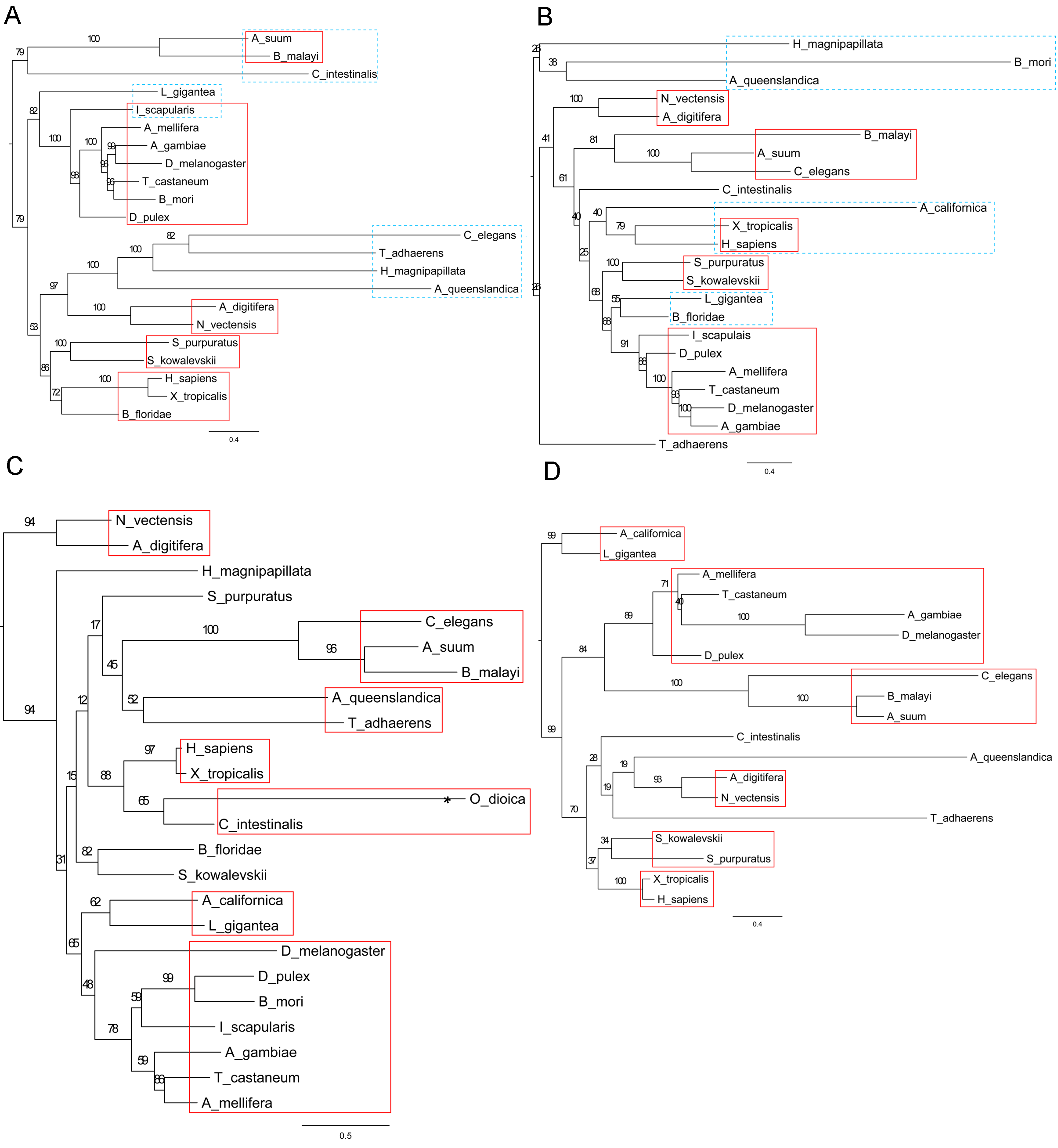
**

**Supplementary Figure 6 The phylogenetic trees of *fat* (A), *dachsous* (B), *hippo* (C), and *yorkie* (D).** Columns with <=2 amino acids were removed in all datasets. Trees were produced by RAxML (bootstrap replicates=100) based on the substitution model VT+G+I (gamma category=4). Trees are shown in scale, except branches with an asterisk. Numbers indicate bootstrap values. Red frames mark species correctly grouped; blue frames mark species grouped in the same way as in the PhyloBayes trees.

**
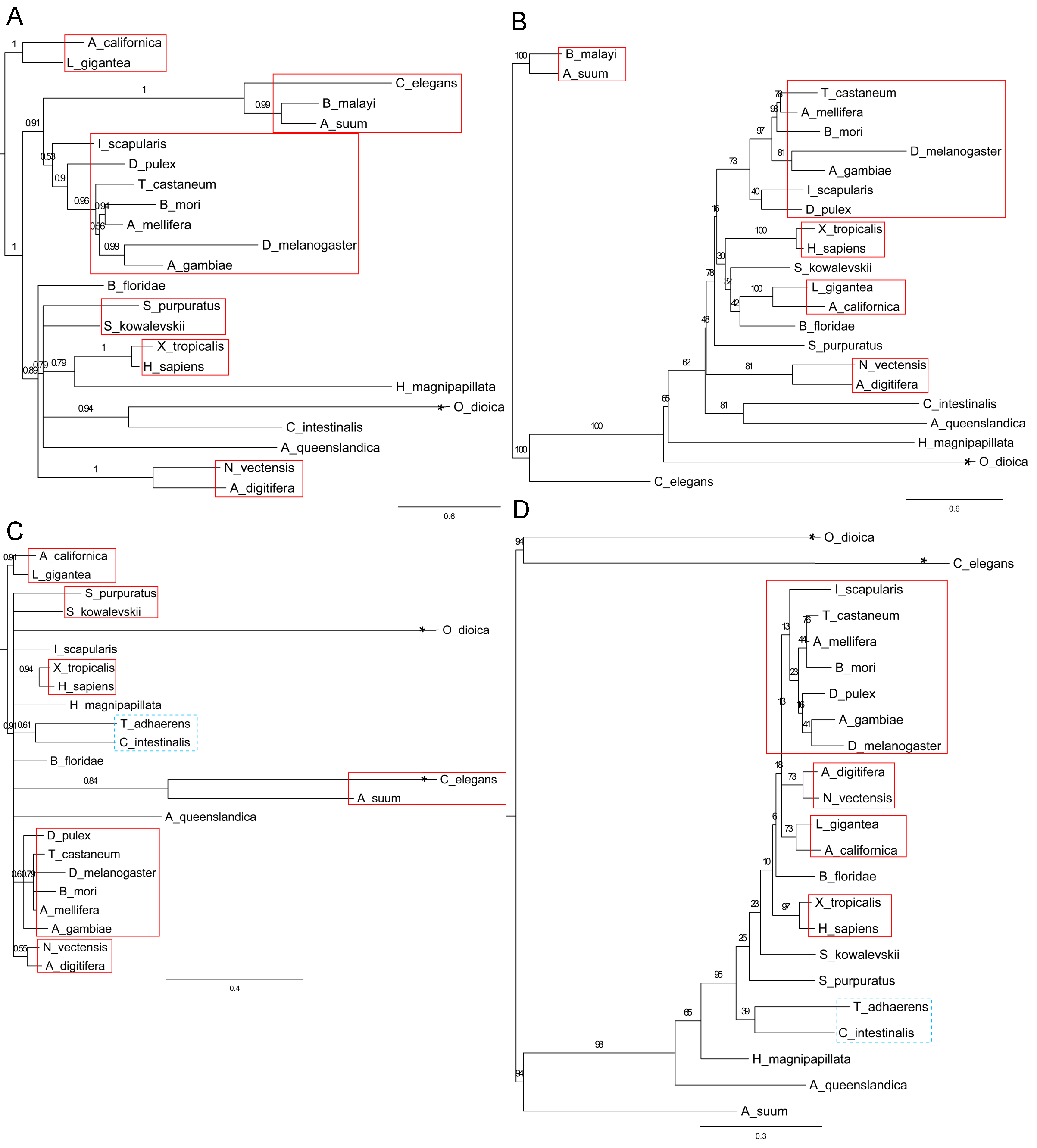
**

**Supplementary Figure 7 The phylogenetic trees of *merlin* (AB) and *mats* (CD).** Columns with <=2 amino acids were removed in all datasets. (AC) and (BD) were produced by PhyloBayes (after 2000 generations, maxdiff=0.083 for *merlin* and 0.0758 for *mats*) and PhyML (bootstrap replicates = 100) respectively, based on the substitution model LG+G (gamma category=4). Trees are shown in scale, except branches with an asterisk. Numbers indicate posterior probability or bootstrap values. Red frames mark species correctly grouped; blue frames mark species grouped in the same way in trees produced by PhyloBayes and PhyML.
