# Supplementary material for: Hippo pathway genes developed varied exon numbers and coevolved functional domains in metazoans for species specific growth control
Source: BMC Evol Biol. 2013 Apr 1;13:76. doi: 10.1186/1471-2148-13-76 (PMC3620549; doi:10.1186/1471-2148-13-76)
Supplement: Additional file 2 — Predicted gene sequences. [file 1471-2148-13-76-S2.doc]

**Hippo pathway genes in metazoans developed varied exon numbers and**

**coevolved functional domains for species specific growth control**

Henan Zhu ^1^ Ziwei Zhou ^1^ Daxi Wang ^1^ Wenyin Liu ^1^ Hao Zhu ^1^*

Bioinformatics Section, School of Basic Medical Sciences

Southern Medical University, Guangzhou 510515, China

**Predicted Yorkie sequences**

>Amphimedon_queenslandica

MTDIINTNSPSSESGKNVILKVSENSDAQLEDLFKVLNAGDSSNQSARKRGLPPSIYEEP

NKDEQQKNTLGPSIQLPQNPAYGPGRNNAHSRVLSLPADLNRYPRGDEYNYHNIPLPPGW

EIGKTPDSHTYFINHNERRTQWEDPRLQMMGLSRGDHMAGGMVAPHNMAQPPYNQQQTTP

TGSLTHMAGPQPNFPHYPQHRHVNNQSLDHTQMGGMGGIAEGGELRSHLHPLMSHQYSQS

YGGPTPIHRPMSSMPAPMGMHPGMHVPLSSKRSYDLTMMEHKPPVTLGGDPYLSEHARQA

SHDSGLGYPYQGDQGMMEFEDTFDGGLHSLPPHPGLPQEQGGPPNHGLMDPLQGPPGGEV

EPHPQMDFGGDMLGDFSNNQGMFGSNTWV

>Acropora_digitifera

MERTNNNCVVHVRQDSDTDLEALFHVVNPTTVPNSHPDTTPANSLPMRLRKLPPSFFKQP

PIDGGLSPETDHPSGLQISHSRAHSSPASITVPSSLKGPPNHSLSSVVHQRSTSFDNTAL

LEEPAQMPPGWEIRSTPNGQHYFMNHFDQITTWQDPRKTQSTSNLNSAQTSASLPDGWEQ

AITPEGDIYYINHIERTTSWVDPRIALQCRSQENVRSSSIMPEMYRHRTIQLHRLQRERE

QLLKRQQELLKQEIKLKRDILEEGGTKSSLLGNLTREGLLPPHQDSTPVTNGGGHIRDQS

FDSGLGMGGGNYHDIDMNESQPMFDANYNSKDSSYRTDASRRLPEILDSLPATNVDLGVM

EGNDSSSNMDTDDLGVGLEFNSEMLNDVENFMSPGNKMSDNFLTWL

>Aplysia_californica

MSQDRTGPIVHVRENSDTELEALFNIAMNPQLASETNKSVPLRMRNLPASFFRPPEPPKQ

NMQQQMGVNASKDGHSPADAPVFHGAVNSSVNIAHMRAHSSPASLQQNLSAAPPPPPTSH

VRQHSYDLLDEQPLPAGWDMAKTPQGQRYYLNHVLQITTWNDPRKTHTPGTTAPSGSSSS

NNNNNNNNTSNLNSSPQTGSPAPSSQPAPNSTPVNVDKVPLPPGWERAFTADCEVYFINH

IDRTTSWFHPSIPVHLQRPGMKFQQQQQQQQQQQQQQQLSSGPISPQQEQLKHLKIQQLQ

MEQELLKKRQDEIARQEMALRAQVNENMGTSGGDVTAISQSSEMTSVADPFFGQTTTSDH

HRQESGDSGLGGMGTSYSLPRTPDDFLGNMEDMDTGDGGPKLTGQADFSSMDLAGVNDVG

DHLNMDSEDLVPSLQPGTQSHLGEVRKIRVKCLSQGHNVGPLMGFEPTTLRLRVRETDH

>Lottia_gigantea

MSQDRSERKGSQIVHVRENSDSDMDALFSYGLNPTPDSNKSIPLRMRNLPASFFQPPEPH

QAGKEGSTDSTGYSSSNPTSTPNFSVNHTRVRSSPASLSQGQTLSTAPPPPCSQHVRQHS

YDLAEEPLPPGWSIGVTHVGQRYFLNHETQTTTWQDPRKTQSTTNLHNPQQQQQAPPAVH

PLQQQQQQQQQQPATQQQPAVSSSPNQPTQIVNVDKVPLPPGWERAYTPEGEVYYIHHVE

RTTSWFHPSVPAHLQRPGMRLQQQAQQQQQQQMQVNSPGSSPNMAQCQQGNSTDRHRQVQ

IGLQQLKIERERLKKREEEIARQELMLRTGNIQHPASTENITDITISQASEMTSVTDPFL

SQSGTSDHTRQESSDSGLGPKLQSQTDFGTMDISNVGSVDGGESNMESEDLVPSLNEAIS

NDLLKDVENVLQSNKMDNLLTWL

**Predicted Dachsous sequences**

>Acropora_digitifera

MSVMKQVSLLAAGCQPWNSDVCAASCNGDRFAYCATLAVYVYEVSRQDTQAGILRIWNVS

KSTPMTSFKLKMTGFHALIVVEAFEAMASNNNSQTSVALSSTSAASEPPSSPNNITYALP

PANILCTFLDGGVGLYDLGKRKWSFLRDMGHVETIFDCKFKPESPDLLATASFDGTIKVW

DVNTMTPHGKHTVYNVSWNHKDSRRIASCSSDGSWDGTCLETVLDHGADVYELAMSTGGA

PLLNGRISRELKATASAKGNQSHLAAVKLLSDFFTNTKLAYCEVRSQVVELKNKTVLSLQ

PPCGSRNLWELVLVLCEGDEHLLPVTYRKGIMHTKHLVKHKASEAQELETVRASSSRASG

LMNKKEDRIKEAASLHVKLGQLQRYCELMVELGEWDKALAVAPGVSMSYWRELMERRSNQ

LMREDDDAAVPYCASLGDAGKLVSFFTSRGQLHDALLVAQVACEGGLNLPSTKKVEKKPL

DLKKNDILPSLINHVTDENKGLLQSTSTALADWYFYSGQPVLAACCHLAVGDAKAGLTSV

EECLQNAETSLGNNKFLDAMKYYLLSSSPELALDIGLNLVRDAMRKPYWALDDVFGVVQL

LSCIRTDRLQQSRISKQRQELLVLSAYLGALFAIRRKYYPIVHPLFKHARQLYRSDKVDL

TVTTSEIETESEAWLLFNDPFNHSDVQLSCLTGERIKGPAYFLNDGRSVMSLNNALMWAK

VNAFSLVGGVWRTNMTTVSLIWKSNIAVARSCRDVNEAIRDGAKLKFEMAFFAVVVVLAL

KIRFQGYVGCITGNVVRKTRSLPNIAKEYVKAMLQFKGLKSPGSALGLPHILQAKRLEVI

EKCSQKGEVNAFKITSSFLISLNVCGLKTYRRLWYAVLLWTTLVFDFHPHCTNASLVSSF

FISEGLPVGSLVGNLPHITFDNVLEVTPTEGANDFQVDKSTRIISTNIILDRETIANYHI

TLLKNVPFALYSVYINVTDLNDNIPTFPQAVYHWTLFEGSPVQRTLQAKDGDFGANATVG

YSILSGNVGNMFMLREFRDRKGHLSAELYLAAGKELDRETRDSYLLNISATDGGNPAQVG

STLLNITVGDINDHKPVFSNSSYSVNTLENSPRGTTILQVFATDKDIGSNAVIEYHVEHG

SHSDPLFAFSADPKTGVIINNHILDFEDQRVYNIYVRAENPNSGMFTLARVAIHVVDQND

NKPTISVLFELSGRYQVFENAPVGTVVARIYVSDKDSGKNGEVDVTLEGGNGYFSARSDP

ANNVDIIAVAKNLDRETQERFILRIVAKDRGQPQQTSYDSFIANVGDINDNYPVFDQAVY

GAVLSENVTIGTQVVRAEATDRDLATNAQLVYNISHSLPTDYYKTWFQINSQTGQIKTAA

LLDREKIEQVVLNLTVSDLGVPPLSANCIVLINITDANDNNPSFSRAVYFATIAENKPIG

TSVEQVSATDIDIGQNGEVRYSLDNQQGNVPFDVDSSTGIVSTAGLIDHELRSSYNLKIV

ATDGGGRKAKCDVNITVQDVNDNFPVIDPLAYDVKVYENLTIGDAITTIIAKDDDSGLFS

RLTFSISSGNADSIFHVNPSSGLVTLLKALDREADDFHQFNVSAEDGGGLKSKNTALVQV

TVLDVNDEPPIFEASSYNFTIVENSPVNSILGSVFASSKDLGTNADIFYSVKSGDTDNIF

AINSSGTIFTQRNIDHEKAALLVLNIQAKDGGSPPLYGFANVTVTVIDLNDNSPSFLSND

IAINILEDTKVGEVFYNVSASDRDSGLFGQVQYTLLSNPNNTFQLDQYTGALSLTVPIDF

EGPRHYTIQVLAQDGGSPPLNDTASFKFTVIDVNDHAPKFSNSTYSVHVMEGSVTGNILN

VVATDADSGDNARISYSFQPGVDRTVFGLKPDGWIFIKHTLDRERQAVYRFGVTASDQGN

PPKSSKANVVVHVDDANDNDPKFSQNQFFFPVAENKSNRTKVGQVSATDSDAGLNAKIVY

SLVSQSPQFVIDPDTGIIRTNQVLDREVQASYDLMVEARDQGASPRTDKSKVTVVVKDVN

DNSPTFRNRSYSQAVPEDIPLGSFVIRSKNSSTCVFTGTKICFRETGVVQTVGPIDRESV

DEYTLTIRATDQAFTESERLSSTLTVLIIVLDINDNKPDFVSPDRTFVLEDEPFGYPIIT

ITSIDRDQGSNAEVRYSIVQGNSGIFSLDPNSGLLKLHGTLDYERKPTYILEISAPDLGT

PPQTSYQNLTIDLVDVNDNAPQFNQSLFLGNVYENEPVGTPVMNVTAHDPDSGSNGALSY

NIPRGEVMPRFVIDPKSGLISTNSTLDREEKDVYILTVSASDNAFPFRVATCTVKITVLD

KNDHSPVFNPTRLNLTVMENRAPFMFYVMSAQDPDVGRNGRLRYSLRSGNEDNKFTISEF

TGELSTTAELDREKTPQYDLHVVASDITPPFYNSSAHVSIFVGDANDNRPSFLQASYDVN

IRELTPINTAIFNVSAVDRDVGLNGEVVYSLSNKTFGIFRVDSRTGVIYTQRQFDFSVKQ

AYTFNCYATDRGVVPRRGSTEIRILIIDENNHAPVFQRLPYSQQIQSSFPQGMRVLTVSA

TDQDSPSITQMSYRLIGNSSYFLLTSSGELQVKHGVYSIPNGTYVLNILADDGGGLSGRG

VAEITVGPIMDNPPVFVNSTPANVSIPENSPNDHEVARVIATRSGSSSGIVYSIINGNDG

SPFKINRQSGVVTVANTALLDFERLRYFRIQVIASLESISSPNAYLTLNVYLLDVNDNRP

LFNPADISVQLAEDDALHSSGFNARTVVMVTATDLDSGSNQQITYELDSGNVNQRFAIDS

KTGVITTTKLIDREVLPYYRLVVKATDHGNPPLSSTSDVSVNIVDVNDNIPSFSGPYTAE

VVEDVKVGYVVKRVAASDRDASPNLVYSFSNGMSNKDLFHIDRSAGVVSVSDSLDYESTK

SFVLNINVTDGTYQSQTTLTVNVKDVNDNAPRFLNSSYRATLSEDSAAGAFILRVSAVDK

DSGTNGEITYAFVTDVPQFTIHADTGSIHTAQKIEVGAQESLFFVVVSATDRGSNSLKGF

ASVAIDVTDLNDNIPLFHIMNPLSIAVEENSEVGTQFYQVLAVDTDSGINRELTYSITGG

SGENLFIIDHKSGYLRTNATFDYETKSFYFLLIKAVDSGSPPLSNEINITVYIKSVDEYE

PAFLNSTYKFEVPGNAKIGDFVGQVSAVDKDGGEDGVVRYSFESSNAKVFGINATSGVIF

VNKSLGEVESKRKRRSIERYEQGVGSRRVRRNTDLEIVSLKIRAHSGKSGSKFSTVNAEV

AVDFACPGCIPGRVDTQSGNALSPIIVLVITVSGIIFIVIVVVIAAIAIYKRNKRKRGRP

SSRSGFDGSFDPISVYPSLNGNVNHVESSLDNVSTPRSASQERSPLNVPSDSPTRRTNST

DSPNSASSGRGSSDGVDFEVDHPATLAKGDLGSLHSENYVRTLIAPDSGIHQDSDQASQL

TISDTSSVLQGEGLGVDKRQDKIDKILARLGSQESLHVFGEEGGGEADGGVEVGNLLYAR

LSEVDADEDESIIDGIRPFVDEGHDHPSYGGSLSSIVGSREELTGSYNWDYLLDWGPQFQ

PLADVFLEIGKMKDDSPPKKAMSLSLQSSRDLRNLPAGVVTTDMLSSISSLPRSPISPPS

TRYTSPAFSPNFTPAITPLVTRSPSVSPLDTGTSTPAFTPASATPKSGSRPASMHVLQLR

RESHDGSNSDLTHSPSISDNESNLEVDV

**Predicted Dachs sequences**

>Saccoglossus_kowalevskii

XASSSEVVLLREQLTALSSELVSLRTSIAVKTANGTTNGCHDGLQDAEDLGDFIVGSEQQ

SGLSAVNGKAENTASIVKRKENKIDLEDLIHLYGPLTEDSILKTLQHRWNKGCCYTKIGP

ILLSLNPYKGTPIENALHHGGENHPYLLNVVKDAVRLQTETGCSQAIVLSGESGSGKTYS

SLLLLRQLFDVAGGGPGTDAFKHVSAAVTVLRSLGTAKTVHNSESSRMGYYTEVHVTEGV

IFRTKIHCYFVDETRVIQTSPHEKGYHIFYHMLSGLSQDERVKLHLQGYSSHNLKYLNHL

LNVDVDEDCQKFQVWKSSLQILGIPFSDVMRILAAIMLLGNIEFVESDGLELDIKGNNEI

KAVAALLGVSGVSLYRGLTTRTRNMRGQIFKSPCDAPTANTTRDNLARALYCRTVSAITR

KANSLRRPGSNSGHSSSSDDVHHSGENQNFKHPSPATSKLSVNQSSSASTHCGGLGDGII

SIVDMFGFENTQNNQFEQLCSNLCSETLQHYYNIHVFKSTQECCRDEGIPCEVDLDYQDN

TPVIELISAPLTGIFHYLDKESANMESKSTCASFVQNIGAQHSTHDRFFIPEERSTYNFG

IVHYVSSVVYSSSNMVERNRDIVGDDIINIFQRQNCNFGFATHLFTSELKEQNKNGGIPK

GNYHRILPSPSSYSDTVSNGEGRRTFSQDFQSRLDNLLKTLMHAKPHFVLCIRPNYQEAP

SIFDLNVVRKQIRALQILETVHLMAGYPHKMRFRSFNHRYKFLSRHKRLAILDSFLKALD

DSKMPYTSTNWAMGKRHIFLEGARQSLEHLRYQKREEAATVIQSHVRKLACLKNWPSLKR

TLEQKLRIRTQNHEQLMSRRSPMYQEIERSRKRVIENESCDIKTIQQTCYLYGLDMDSPP

PLPSSRSYTVTGNMKMGFPQCRVMKHSFPX

**Predicted Fat sequences**

>A.queenslandica|from NW_003546491.1|GENSCAN_predicted_peptide_2|4718_aa

MKRDFLSELVVEVNFLIFFSLAVLPSSSQQLHVPVQSTELVSINTFIYNISSKFYLLTKR

TDLHYKLIPSTQCSVFNVTSSTGLLFTQESINITDADSSTDPLTCISTDTDQYPVLSSYN

CFIVGSTDSYSYGLVVNVHVLPTVHHSLPLSVGGEAVGGLENAPLELDGFPLLVDYKTES

NELANGYLLLNANDSFSINITTASCYSVLQLVTTRPLSIGTSYNLTLYHMPTQSGTQILV

NVVGGNRYSPVFMDVPSSVTISELSPVGSLVTMVTAFDNDTNGYITYTLYPLSSSFIIHH

TTGLVYLSQPLDYESSTFTNITVIASDGGYPRRSTEISISIFITDANEVRPAIDIQQPLT

GHHEIINDTIPVPESTTTGSILARFVLTDEDSSHISINMDCRTCHNCFTLSLVNVTSNTS

NTGNTGLTAVYNLLLTSRLDYEGYTDTHVILINATDNGAPPLTILRTINIQLTDTNDPPL

FPSPFYEGRVSSGSPIGTNVLFVTATDQDAGDNGTLVYAMVNGSGSFTIHPSTGLVSVHS

SSLVPGLVFINVSVRDTGPSPLYDYTSLTVLVTESDTHPPVFSNPYPSTSVSEYWNQSLP

LYHFNASDPNTGCSGSVHYSLLYSEPPLFYIDQVSGLLYLNDSLDYELSSTARVSVSARS

LGNSPDLVSTATLTITIQDEDDSSSSFTPSCPCSPVFLTPLYAGVVREDAGTGVEVLRAR

AVDYDQGDVISYSTSGSADVIDIDSSTGQITLASTLDRDIDYISFNVIATDQTSSSSSVP

VHLKVLHVTNSNRPSFVSPSNGTIINVTESTNTNQVLITISATDHDIGVNAELRYWIRFG

NEHGLFYLDSVTGKVWLTRPLDYETDPHSYTLVFDVTDLSSSPLDTGGQLLTVTLNLLDI

NDHFPVFTQPRYDCYIVEDSSDVRNDNGSSGCRIEAIDSDSINNDTTYSINGSSSSYFNI

GPTTGLIMAKDSIVIDYERNHLFVLIVTAADGGTPPHYSTCPVYVRVINVNDNPLQFIPS

LFSVYLPLQLPANATLFNAHAFDRDETNITYSLLGDGTSYTVSTDGLVSIDRLGPVDPIN

ETVKIISLGPDSNGRTNDYNLYYLPGINFNSLPPQFIASNPPLVSIPRRATNGTHLVTMV

TWDPDRGLDGVPRYYIIGGTGVGYFAIHKTNGSIVTRRELSALTTPTLSLVVMATDSGPH

PQFSLFNLTVAITDDHKVFIDSPVYNYTISDKYSFTGHTFGYVHVPVHDGRVINYVIPPA

QEILPFAINRSTGGLSVNGTLDRQWYSFRTVASDSTESTVSVINVRVLVTVNNFRPEFPP

HFSTILLPASFPLNTSFIRLFVTDGDNNNNADSSYSILAPPNSPIRIVPTSGELFLSTTP

SFNSTVITIRANNVDLFSDFNINITLFHADANTDFDLISNNNDSILVPESVGIGSVIYTL

PGSSDGRPLFYYLTHYDDDDDKFTVHSGVVYVTGSMDYEVQQHYTLVFTINDGSNNNNNN

NKYFTLNLSLTDVNDNPSYFSEDNFVFSLPEDALLNYVIGSINITDSDSVTASNVIYSII

DWLHPLSPSLFNITSSGVLVLTGPLDRETISSHTLTVSVQDGQFEQLTRVTINVEDTDDN

PPSFTGLPVTLVVPEDRSLGDIVTIVTAFDLDDPLNSSSIVYSLSGSHQILNLPFQLESS

TGVISINGSLNYESVTSYNLYITAASSTNPPSYSTHSLSILVSNIIDTFPVLYSMEVSVY

ENQSSSNYVTNIGASPPPPHPVTYSIVNGNDLGHFYIEGYTGTIRTLVPLDRETVDYYNL

TVRGSYSHQYYTDISVLISVLDINDNNPSVSVSNLTFLVSEDANVSNQIIFDLNPIDLDQ

GNNGSIRSILILDSESDTIFDIDTSGHCTLKRQLDREEKETHQFNVLLIDDGHTHPLYST

YLITILVTDVNDNDPIFSNYQSTLVVSGPILSNEVLYSFTATDTDGGANGTTQGYFISGG

SGEGVFNIDPLTGDLRGLELFEMESNYSLMIGAVDGGGRRSIINISLIISYCRFNRLSFT

PPSYSLSVAEDTPPSSLLLSPSITDFNSPGVFRYSLSVHDDQFMINTTTGSLSLLSSLDR

ELTPVHYLVLIVQDISANMLRVAKTTVTVTISDVNDNAPTFIGLPYAVYLSDTQEIGSHV

FTVLASDADTDNNSDISYYIISDPISALGINSSTGVLYYNQTIDPAITDIHITVVIGAAD

NGHPQLSSNTTLRLTLINSNAPNFSAPLYTATLPEDTAAGTTVVRLLAEPRTDGALLFYS

ITDTGDIRFPFSINPESGVITVNDRGLDYETTPSYSFTVQAEEIRTGLYSQATVQVSITD

INDQVPSFTQSSYIINIPETISKGSTILTVEAADGDTPPNAQVTYSLSPSTSFTIDPSTG

DITTNSTLDYEVRTSYQLTLVARDSGDTPLEGSASLRIIVTNVNDNPPVFVPLSPISVSE

GADPGTVVTFIRAMDADNNEIIYSIIPEQDGGDNNFQLLTNGLLRLNPQNVSLTDIQYTL

NVSASDGVHVIFTSVTIDIDDINDHSPTFNQSEYSASVVENSPSGVGLIRVNATDSDRDI

NAEITYSMIGSLFTIDQKTGLISTSSSNIDRESTPTHSLIIIARDGGGLTDTATVVISVV

DVNDNSPSFTQSLYGALLAEGDDYNNHQVLTVLARDPDEEINGSITYNIITSHFNLFHIN

PSTGVVSVTGSLNYETDSSYSFNITATDGGGRVSEVAIVTINITNVADTNPYYSQPVYYL

LVPEDTPTGPFYQPNVTFAEGCTPSGYSIFGKGNGPFSYNSNGYITLNGLLDREDTDSYT

FFVTVQCVQVNLLTNPPTFQTRYGGSTINITITDVNEWPVITSALYISSIISEGSSINST

VTIVQAVDTDLGLNGTVQYRLGSSDTPFSVDTVSGALLVSGTLDREQQQLYTLNVIAYDL

GTPSLSSSATVIIAIQDINDNPPSFICTNDTNGVLIDSQCHYSISIPEDVELNQLILTLG

TNDSDITGSTSFLLNSTVFNITSTSEGNGVIRTGAGLDRETNDHYELRVIANDGVFTVEA

FLSVIITDINDNRPFFSTGEYVVDIIENYSTLTVFIRLNATDEDFGNNSIITYTLLTNPS

SANVSLNSSTGEVSFLISPDHETASRLEYYIRASDIGGLEDLATLTINIIDVNDNTPLFT

GSNYTASIYENTTANVEVLLVSATDSDSGSNALIRYDIDLESSQYFTIDTVTGLIRARAH

FIDRERNELFNILVTARDSGEELSLHSSTNVTVFITDINDNPPLFADSSFTVFVYENASV

DTVIHSFTVTDDDEGVNAQLSYDIAGTGKESFLLQATPTGLLLKVNGELNSESVELYSLV

LTATDGGIPALKTSVLINIHVLDQNEHLPTFNRPLYTFTKPEDEPSGSVVGTITASDLDP

ADSNLTYRFKGSVSDFSVNERTGKIRIGGRGLDFERVRNYTLILLAIEPRPLSPQTAQTV

IEITVTDVNDNPPAFLCSHDHTHNCMNHSYSVRENIDNVILGRMRVIDADTVTDPNQIRF

SIASGGVDINNRTLFSIDSITGNLSLLSPLDREERDSYVVMVTASDGGTPNLIGSTQVLI

RVTDVNDNGPRGGVQYIIINLLEGDLGSLLNEAVFTNDSDIVNNYTLTVTGGVSDEITIN

DGLITSTSVKLTPGQYSLPVGITDVLYNGTIVHTRTLISIIINDISSHVQDNSFTLLLSE

ITPLTFLSDFLIDFNTTLTSLLTQELNQLIQVSYIDIKPAVGPVIPAGTLLTISVCYNDS

SCIEPDLVQHFIHSNKERINNINIVSVDVNDCSKEPCANNGLCSGTVEFSLSKSFVTSPH

LSYLGLVTKGRSTCTCFSGFTGDSCSEPCSDCNEASLCDRVACPTDYRCVINDRGKPECV

DDCNLSPCLNGGRCIPQDPGHHCSCPLGHDGPNCEQTTATFNGQSHVLFPSLPLVKSGSI

SFEIITNESEGVLLYGGHFDVGFNDSLLIYVTNNSLMTSVSFGGYEYQLIVNEFPINVKE

WITVTFEYDINRVWNRIVNLQILIVTGPLVTTVEPVSESGRGLHVHVRMNILEYTVMKII

DGNLQARVSLGGGRETRLLINKTCINDDRGHMITFTRKEGAMTLILDDTYTAKNYLPTTN

NISNDVLLDVSHLSVGGANTSNGRGYTGCIRKMRLDGYDIPVRGENEAYKANSDTSIEQC

PDIEEPTPPPISTSPISSLYVVTGLALLGLLLISVSALFVCKSTHYWYNKKKGTVSISER

RFIDYPSTLPSSTVSSVVNSRRTFGLQSTTSFEPLDVTGRYGYEAYRPLSPRPPSPLLFP

PSELKPSVTEAPPISRPTIAPPTVRNDTRSPSSASDSVFEVFNEYELGPASPPPRDNNKL

ATPPSPPSLPPPSPPPPPPVSFPPSVVSKSSEALDPMTGMPVSHDASQESLTAHTEDDGR

STLVDGEDIGRYVKKRLMCADILVNDINYDASTSFDTEGPYSPLGSIGSLYDILQDNDTN

YKYNNNVLQDESAPRSIKTPPTQISHLLSVPTSPTSPKRPSPLLQSRQWRDNNGVPLPPP

VRPKPLRKNQTTSTTDIDVSIGVSKVAVTDSGGATGGAVPSSKDLSSSAPPPVPVKKQAT

KPSPLSPPLFQAVRLQPLSVTSSLHQSSRDRPLPHGPSVAPSTDNKRVLVPVKFSSPKRS

SGDTVKTTRGGGSSIQNRATQRMNNFTGEFNTSQESDV

>B.mori|BABH01041846.1|genewise

LQVTIQLKDVNDMAPEFITPHVTSISENIPLNTVVMTIKAIDKDEGRNGYVEYFMMPNPE

INGYFSLGNVDGIIRATGKLDRELKSNYIITVTAKDRGDPPNVTKMDIKISIIDENDNSP

VFDPKQYSASVPENASIGASVLQVSATDVDEDANGRVRYSIASGDENRDFSISEDTGIVR

VAKNLNFERKSRYLLSIKAEDCAKDDVRFDTAEIAISIQDINDNPPTFLDSPYLAYVMEN

VIPPNGGFIISILAYDADSPPFNNQVRYFIKEGDADVFKINASSGQISLLRTLDREVQDE

YTLALVAMDTGSPPLTGSGTVKIIVQDVNDNSPEFERQSYKTSVKENLPSETMIFHAKAT

DKDTGNNAKIRYSVLGDKSERFKIDPNTGMLYTNETLDREEWDVYYLILMAQDSSTTDPR

TATANLTIVVEDENDNTPTFPHPVYETQISERTMKGNFVFGVKATDNDIGLSKKIEYDLE

GEHKHLFTINKETGVIKAKENLITYKDKSISSFNLVITAKDSGTPPKESSAELILILKSV

RNFPKFSVTNKLSFTFSEDTPEGVLVTRLSATSPKSGPAGLLQYGIAGGNIGDALRVDQM

SGEVFLTGKGLDYETMPLYEVWLEAKDSDNPPLKSFIEVEIKVTDANDNAPIIENSLYNA

TVLEEESPPQLVAVIHALDYDSNENGRISYKLVNDYDETFSIDSETGEIYTNMALDRESI

PYYEIIVEAEDHGLPQLSGTSTVIITVLDKNDNPPRFTRLFSVNVTENAEIGSFVIRVTS

SDLDTGSNANATYSFVENIGNKFIIDPISGNVTVAGSLDREIQDEYILKVAAIDGAWRSE

TALTITIQDQNDNAPEFEYSYYSFNFPEMQFKNSFVGQVIATDRDKQGPNSIISYSLQQP

SDLFSIDPATGEILSKFTMNYKRTSTNSSPENTYSVVVVATDNGKPPMSSECLVIINVVD

ANNNAPKFRDHEKLIPIPKEATIGEKIIKMKAEDNMDFGINAEVEYYVSGGNGSSYLTID

PNSGWIIVNKQFYYLGQYYELKIKATDRGVPPQSDETSIVFVVTGENMYSPKFTALSYQV

IVPENEPVGSSILTIQASDEDNGPNGIIRYSISSGNEGNEFQIHSISGAISIMRPLDFDT

IQEYRLNITANDLGFKSKKATATLTIILTDINDNAPFFNQTTYVAFLPEISPLNTFIYRV

TAVDIDSPKNAIIKYYLNSNMLSLFYIDENTGEIYSKEVFDFEEKNLYDLTVRAENPDSS

MKNTTNIIIHITGVNEYYPKFKQPVFHFDVSESAEVGTNVGVIQATDQDSGDDGIIYYLF

VGSSNDKGFGINSQTGVIRVARYLDRETQNRVVLTVLAKNSGGIRGNDTDEAQVIISIQD

GNDPPEFVRLNYEATVSEGAYIGHEVIQVQAVDKDVRPQNNQFSFSIIGGNIEQSFKIDP

QNGKIEVARKLDREKLPAYSLIVGAIDTGSPPQTGTATVKIMLTDINDNGPIFDINSFEG

SVYENEPPNTSITTLSATDPDLPPNGAPFTYSIIGGKHESFINVNKHTGVLSTARKIDRE

ATPILEISIQIEDSGTPVMRSKYDILIKVLDRNDNPPTPRSVHVVVYAFNNKVPNGKIAD

VKPNDPDIIGDYKCKIIRDSTSDRTLSLLNIRSGCDLYTNTIKPGQGYSFSVLGNDGIHK

DVISSVSVEYFSFDNTTVEESVTIRVMNMTATFFLTHFYRSLLEILNAKLDKNKETIFLY

GVNEVPNYLDLTVALKGNNSISKKESTEHYLKNKEFEITTLLKHEITIGYYPCSSHRCQN

GGLCTNSIKILEDTKIIDSSAIILSSPFVKHDYECHCAEGFMGKNCEKRQDPCSPNPCRY

GGHCRKQGHDFLCTCPVRRDGKTCELEKNDVCSSSPCKNGGSCKESADGSSFFCLCRPGY

RGNHCEALVDSCRPNPCLYGGICVSLKPGYKCSCSDGRYGTHCESTTFGFDELSYMQFPA

LDASTNDITIIFATTKPDALLLYNFGAQTGGRSDFIAIELLNGKXFFSFGGARTSITSVA

ISNPNKNLADGNWHKLTATRNGRVISLSTTSCTDHGDICMECEPGDDSCYNDDTGQAGTL

NFNNEPLLIGGIHKADPLLVRPGQVHCDDFVGCMHSITINGRLLNLSNPCKXRGVEANCD

RSEKGTCYKKAMCGLGECIDRWKSNLCKCDGTFIAPDCSAALQSISVGETGLISFSISEK

HRRMQLLDTFYSGNTMWEKHANKVLSKINTRTNSPAKMLSFLFRTHRKDGILFYAATEKY

FTLIELHEGKVSYTSKQNSVVNMTQNEQNDVSDGTWHNITLLSFGRSIRLLVDNNDVGEE

LDAAGVHDFLDPYLTVMSLGGVKAELLMTSSQNKFEGCLANFSINNEIQPFAGNGSIFKE

TILKGKILNGCHSAFGIGAAQNPDPLRIGITLVIVFFVILLVAISVSIIFYRLRKQKKEK

VGGSTGKMNAVHSKQNGGTSMSNAPNLIAGTNESLMNRGLHNNDTSLNSYISESVDITRN

VGHIVGPELLSKKYKDREIMNIDPLRPQRPDIIEREVVGKSPALREDHHPPPPLSTNTSH

HHDHPSGMDLNSEIPEHYDLENASSIAPSDIDIVYHYKGFREAANVRKYKATPPPLTGYH

HKHQTPQHRHSPHHPGGYPSRVLQQASQPPPQPRQHQTTPLARLSPSSELSQQPRILTLH

DISGKPLQSALLATTSSSGGVGKDALHSNSERSLNSPVMSQLSGQSSSAGRKTPSATPQV

PQVVSVGSGAVGLTAEEIERMNARQRTSSLVSTLDAVSSSSEAQRGTGVGHHMSHRHHSP

QDDNRSSTGSDDESGNDSFTCSEIEYDNNSINAEKLDDVRRPNVSNANNTKKSILPPPYE

SFDSSFRGSLSTLVASDDDLVPHVSSALYRQANGSPASAALGWDHLFNWRPNFESMIGVF

KDIAELPD

>N.vectensis|from ABAV01018189.1|genscan|5175_aa

MVSQIRTGIPLVRIRSLWCKASLVSWISLWMFAASVSAGSSYTFHVQEEQPPGTYVGKVD

GASAFEGSPKQFGFNSNTGVISTTARIDRETTTQISLQLRTLNPSNQPISVQVQVDDIND

NEPRFPTLYYTLRIRESMDVNTTNPINTATDPDAGNNGTVDYTIASGDNTATFSLGTTAC

PTGSTLCVITKKPLDRETTDFYQLNITATDRGTPALMGYCLINITILDENDHAPVFTQNS

YLGSIAENTKPGVSILRVSAADRDSGSNGDIKYAIDWDAIQSNSIFDLDSSTGVIRTKQN

LDFEVKNSYTFNVIASDGGVPSKNDLATVTINVLDTNDNSPQLKITYLPPHNGDKAIRSE

NSLINTNIAYILVTDKDSNTNSQTELKITNGDDEGCFKVEFLGFYFIQLQKPLDRELVPF

YNLTLNVTDKGVPSRSTLGFVLIDVTDVNDNSPAFTSNAYTANISELAQNGSYVLQLNAT

DPDEGDNGTVSYSITGGNDLGWFQVDFQSGLVTTAKPLDREQVSSVTITITAQDHGSAPK

TGSTTVTVTIIDGNDNKPRFMKSSYKGTISEGASIGATVLQVSATDVDSGDNGRITYEIS

SSDAQVNGLFELNTQTGVLWTKSQLDRETKSLYSFTVLAKDHGDPQLMSSVKVELTISDI

NDNAPVFYPVEYFASIMENEPPGKLLQVTATDADEGTNAVISYDIIGGNNNGNFSIDSST

GVISTLASLDHEVEEFFSLIISAKDGGNKYAAKTAVVNVTVLNKADNLPVFLYEMYNFTV

YENVPKGTLVGRVYATTKDKNGSISYAIVSGDPTNLFLVDSTGGIIKINGDMDREEKAQY

LLAVIARVGTVKPLSAQATVNIEVLDRNDNAPQFSTSSVIVDIDGTWLVGQVVYNAAAVD

LDAGKNGLVRYQLTSNAEGLLWINSTSGVIKLARKLTDGDETQYTVQVLASDSGSPPQHS

TMTIHITLSINHPPKFTLSSYVTHVLASQPLSYQFFYVVAVDQDSGKNGQLFYSIPQGNE

EGLFGIFPGGKLYIRKSLLQALKGSYDLIVRATDGGVPSFSASVPVTIYIQDSNDHRSLF

VNNTFRFAVAENSSPGTLVGTLVAHALDHSRTKDIVYSLISSESHFAVDVRSGQITTRVR

LDREELIKNTGSNVYIMQAEAMYNDTTIKRDKAIVIVTVTDLNDNAPVFSRAVYAITVSE

LSLPGSPVFRIKAMDADEGDNAKFFYSLVSGTGMDKFFINSSTGELHLSKTLDRETTSSY

NLVVKATDASNANLYSTAKIEITVGDSNDQRPVFPESDYTVNITEDALIGSLVINLNASD

KDIGQNGLIKYSISNGNLAAMFDVESATGRLIVAKSLDFETTQLYELTITAQDHGLIPLS

STVNVTVNVLDVNDNAPLFPTQPVTLTVRENVNTGFPVGRCTASDRDTGTNAHIAFSMYS

QTPSKTFSVNSATCEITTIAAIDLETMPAGRDSFQLVVMATDQGSPKLSTRKSITVQVTD

ENDNSPGFVSPPAAAAGSSLNVMTITAEDPDRNSNGKVTYSIISGNNGYFNLHPSTGQLQ

MIHQIPDNPTIYQLTIQASDGGQPSRQTQTVVSVFKEGPIGGGPTFSSADYQGSVTENKV

AGTSVVTVLAAYPQSSLNGNIEYYLTSDSSKGGFEVVKSTGVVKTTRGIDREGMGASFNL

VVYAVDKQGPSARTSSATVTITVQDVNDNAPVFTSDEFTVSIAENVDDGTTVITVTATDP

DAGDNAKVSYATYGGSHGNKFAIDASSGRIYTTGTFDRETKDDYSINVTASDHGNPSKSS

TCVVLVTVADNNDNSPKFPALHYVFDIAEDAAVGASIGTVSASDADIGENARLTYSIVSP

EKATFSMNPVTGEITVGQRLDREGVEHYLLNVTATDNGRLNTNDASVDVYINVLDVNDNA

PQFEAAVYHGEISESADKLTPIVTVKAIDKDSGSNGIVKYSLISGNDGDAFTILGNGTIV

NAEVLDREKKSTYNLEVRAVDQATSGGLEGVTRVVINVTDVNDQPPEFTSASVAHVSENS

RVGDVVLTVIAVDADEGSNGDVVYSLGLDIGAPFSLGSKDGVLRVSGSMDREKKANYSIR

VTATDLGTPPLSASMEITVIVDDYNDHRPVFLSHIGTVFIDENASPGTEIITLQAVDGDS

GVNAEIRYAFVTGNSNNSFTINSITGRLRTARNLDRETTPRFTLEVLAYDLGVPRKDSVT

NVTVVLRDVNDNQPVFTMSEYAAEVSEGVVESNIVTVVAEDRDIGSDGSVTYCITQGREG

GLFVINENTGTIGITAKLDRETKSKYTLRVQATDGGSPALSSEADVIVNVLDQNDHAPVF

TPATLTVKVQEDTGSGTTVIKVSAVDGDVGSNADVTYSLKYSFSRFSIDSKTGVITTVRP

LDREIAGQHKLEVLASDAGKPPLQGKLDLDIIVGDKNDHDPEFQQSQFQATISQSAPVGT

IVFVLSARDEDTGENAESDYSITSGNDASVFSLNARSGVISVSRTVPSSPAQYSLKIKAA

NRNAPGRTDSATLQITVSSAVFPRFLHPDQSISISELTRVGMEILTVNATGHTTFDIAGG

NHGDAFDVDAVQGVVKVKTPLDYERYPNYTLIIGAYDGSVPPHVGYARVVITLQDENDNV

PVFSQRVYTAHVPEGKSAGVSVLTVLASDEDSGVNAEVEYHLVGSGPFQVSRTTGLVTTS

DVLDRELVGSYTLKVRAENPGNRSMASEAIVHVTVTDINDTPPTLQQPLTASVSEQAQAG

DLVLQLHGDDKDEPVNSPLHYGFLPNGNPGGVFAIGALSGNITLVKNLDRETKSLYSLEV

FVNDSLHVTQATVKVTVTDINDNPPKFLLLPYVTQLPEDSAVGVTVMNVTAVDADEGTNA

ELNYEFLLSPYGSFFAVDARTGMITLNRAIAFKPFSALPNPNIYNLTVRARNIYPPHYKA

DAAIQIEVLDTNDHAPVFIKSVYLLFAVQDAGSGVSIGRVEAVDKFDYGPNALVRYEAIS

GNGTGRFRVAPETGEVLIASSLRDDVNKTYYIRVRASDMGQPKMSDTAGVYVEVTERNLH

PPVFSKAVYQTSENEDIAVGSALLTVTATDRDFGRNGEISYDIIAGNSQEYFGIGLTNGS

LYVSKPLDYEHQENYSLNVSATDGGKEPKTSYATVRITLRDVNDNNPVFSPIEYRCELAE

NTTPRSVVVCSVRATDRDERGQQSVRYSILGGSGKDKFVIDERTGEIRATVEFDFEVTMS

SFLMIAAKDEGLSPQRTADPPASVHILITGVNEFLPVFVQSVFTATVAENAPIGHSVLRV

SATDKDKGPDGVVLYHLLGSDNQQGFDLDSNTGTLSVSGDLDNERAGIVTLRVIAKNLFQ

NSVTPSSVAHATIVVTVTDANDPPRFLKSVYSAHVTEGSRIGHVVTNVTAVDDDSDIRSI

RYGISAGNTGNAFVMDAVTGFVRTAAELDREAIAEYRLTVTATDNGTPPMTGNATLVVTL

DDINDNPPHLTPLPCIGRVTENKPQGTQVMTLNAEDPDLNPNRGPFKYSISGSDYGSFRL

NVSSGVVSTVKSLDREKQDAYNISIKVTDARGLSAVNFCMIHVEDQNDNRPKATSRDVYV

HTFEGKFLGGVVADVRPDDPDIDDLMTCALLNPPQNNIFSFPRGNCELSSLAYSQDARFD

LSVNGSDGLGAVTYLITLRFLGYTETTLQQGVVVRLRNTNTRDFLANSYHKFTSAVNATL

PSGYSSQIMSVDSSGEGLVDVLVSVRQGGTLMIRDRVSGHLKDKLVSSSALDIAAVDYNP

CTTGNQCLNGGECSSAIQASRRGHVVSSTPVVMVTAGYTWKITCSCKPGFTGSQCEESVN

DCNPNPCRNEGTCTDNNGSFKCSCIPGYTGRLCESDIDECLTNPCKHASACDNTPGGYTC

TCKPGYTGQNCDVDINYCQSSPCMYNSTCKDLPTSFECVCDFGGRGERCEISSFGFEPAS

YMLFPTLQGVNQKTYNNITMEFSTSSPNGLLFYNTDGTPAAAADFIALEIVERYLWFSYN

LGYTGIPEVIKSDRAVTDGKWHHVTVIRDKKKGELLVDGSNKKTGEVDGGFTQLELNQSP

LLVGGIKDFDAYLSDSRKHVRTHDFVGCIRDLYVDGAHLQMHEAREKEGVMNRCPRKSWC

TVSPCDNGGTCVDTWLDYYCECPDGFSGRNCDKAMEAFRFSSTSYLVLTFKESYRRSEKL

NKRQPVSARKRRAVDSVLTDYVRLRHASRRQVSVGRERRAVSSDREEFSIRFRTRQNGLL

LLAQDSSADYTIVQALTSYYTLRAFIEKTIAIASGKVTYSFRAGEASQTLTVTTLDVTNG

NWQNVTLERDARTIRLATGTEFTQREFAVTPHNFLGANIRQWSLGGATQVPSGVTLEKFK

GCMEDLKINGVNTPMNGPNRFLSAEARGGAVTEGCVTSGQCQSSPCANNKEKTACYEDWD

SYTCVSAAPCQPDPCKNNATCKPQKDETFLCVCVGNYTGALCSIPLACQLNPCGEGYDCL

GDGAGGRVCVTAGRRASADNSTNVGIIAAGVFLAVVIIALLVGVVAVRRQRRLKKQSDYD

ANHCEDICPPTDLQDASQRTSPSHSSDDSGVVIRNPSQKSMHLHSSTDESKNIVIHNVGA

PEDYQIKLTKSDERIDHGFSESDVGEFIIRNDFASKHSSRDRINHASREHINHGSRDRLN

QHSNHGSRDRINRGSRDHINHGSRDHINHGSRDHINHGSRDQINHGSSEQLHQVIKQSTP

IEQNYRAPAPRRPRTRTPRNAPGSLARQVLDRRQFGPHRSDPRNRGPSHLHPHRKWRTNS

MDTTSSEEPQDFSDTWPRSDIDNNSERLDYYDIEVASIGQSEASYQYEPSNFKDSMLRRE

MPGLSPAEIERIRQQSRNPPSGSFLDAVSTSSEDAPTMDDKLSSLMERADSSSESSDDTF

TCSEFDFDEKPTSKAEEVEQHGTPHGSMVFNKLTNPPHVGTPQHGSHNTLNLSEDELVIA

NRFTQRPVNGTSDAFNWDDVLNWGLRYHNLRGVYKDIAELPDEEGEEPENEEEYHKYSLA

FYDIGVTMVIYLDFIQTVNSAFVVSLDDLNKMMATRANPLNIAISRQVDDVESGANIEQV

DMSDLKRVVYELFWHVIRGDLRQEDALLVMQEVACIIPVVLLKERLDMETLDAVGLIQSQ

KAFNQKYVKTKTKLL

>T.adhaerens|NW_002050946.1|GENSCAN_predicted_peptide_1|4358_aa

LIRFCNAQFDVYVCKSRTGYGMKQLLVNIIQVTLSSSMPVCVIININTIYFFNSIGDQQL

YELEKVPKCSININRSITKNFDGYPICSLRIICYLMILTHSSYGITVRVPEDANVGYSIY

TMPLPSNGELYSIYQEPSAVEALKLFELSAAGQLRVKKNLNRQQQFNQRQFNLVVLLRRI

SQTSGGTATPLTIVITDSNDHKPVFSQNQYEGYITENQPISTMVKGLINAYATDNDTSID

NYKIIQGDDQNHFAARVNRIHGIAFLTLTTRAVLNREEQSTYQLTIRAQDTGKPPRSATA

IVHLVVLDINDMRPTFTSQNYTFHVDENIPVMSSIGSVTAYDNDDDYNGQIYYYPELPLN

DFMLDYRTGAITLLTPLNYQRRRRYQFRINAVDRGQPSFKAIPTQVIIHINDVTNYPSTL

TSSGNNTIDESKPPQFTQSTYYSNLDVDLPPHSVILVARAFDSYAGSSNDRLIYSMIAAG

SQSNIWTIDRNTGAIRLVGELQVRRYTFQVQARDRLLPRRASIADVNIQVYHQNLNKNQP

QFTQPIYSAVIKEDTALDTSVLKVTARDQDNNEDGQITYSILSATPYFGYFTIESNTGIV

RTTRILDYEFTTQYWLTIQARDGGTPSRSTICRTIVQIVDVDDNTPQFEQVVYNTSLPES

LPPNSFVTVVRAIDIDSPSNAIVTYSLDPSTITNPPTFSISANDGIVRTLRPLDRELRSY

YELRVITSAQTSTLLTIRVVDINDNMPEFTSSNYFIEVPENMGYNPNLLCIHAVDHDEGD

NARIIYSITGGNSDGLFSIHSQTGLLSAQNLNYEQRSSYLLIVRAVDHGTPRRSTSVQVD

IQVTDKSDPPYFDQSYYQANLPDDAGISQVVITIPARSEQSSVLRYSLVDPDPSTDATLQ

IDPSTAQISVKNNDQLDYRRRQLYEFRIRALDIATALSATVPVIVRIEDANNHYPIFSQS

PYWITLNARSSPQQTPLYTVMQLRATDEDQGSHGQITYSFPDPSKPPYPGFQVNATTGEV

TSRYGLNHSKTPLSFSVLARDHGSTPKTSSVTIFLTIIPLSQKLIRFNRTLYQIAVGEDM

VPKNNFFNDIQALNPSGNSNGITYSIVGGNFGNTFRINTQTASLSLVKELDFEKHHQYNL

VIRAKYLPDPSQTSYIMTANVNFRVDVLDRNDAVPQFIGQIPHEVRIEQNWPSGTFVSQV

QAYDRDMGTNGIIRYSSRDHLDKFHVNATTGIITTAKILSGINVPADGYFLNVIASDSGV

PPRSTVILVKIQINTETTLSFSRPSYTATISENSPIGYLIKEFDVISKQKTPQFSFLPSA

YQSYFCIDQRMRVTLQRRLDLEQIAQDQLVITMQGRLGYTTARASVMVTLRDVNDHPPEF

SQTSYYTKIGEDAPVGTTLIQMNATDPDHGNNGQITYSIDSFGSPQSNSYFAIDPKTAVI

RTRRSLDYEQQRHHSITIRAVDGGTPSRQSLARVQVAVDDRNDNKPQFVADTPKFRFIFV

TVPVGKVIARLTATDPDDGANGRVTYSLVEDASKEGRFDVDSITGDVKVVKPLNRIDFQY

GLTIRATDGGTTPKYDEVTLFIGINFPPVTPPYFVQANFLGYVRENMLANTEVVQTRALS

NDRIRYQIVSGDTSDAFAINSGNGWITTRKPLDYEKENTFDLIVRATDISGRDSDIKVTI

SVTDENEYTPLFNHLQYGELPSEVDIGIPIGSTIMQLQAIDYDNSSENTINYQIDTNSAK

EHFAINSRTGVITNLKLMRGVLSPYRFQVSAIDNGKPSKRSQATACVIIINLPPSNQAQQ

VAVRENSPIDTTVTKLTTTLTNPYFIIAFPNITQFKINPRTGLITVGATIDREANMKYKL

ITQVSPITSGIDFTTRIVEVTVIDFNDNKPIFTMPAPYEATVNENAVVGTSVYRLRASDA

DEGSNSLVEYQLDPSSSGPFSVNPVTGVITTTGAKLQLGQRIQLVVRAVDKGNPPQTSDD

AVILVRVENRPPQFTQSRYRATVAESQLRNLPILTVRAISFSGRGIIYSILNGNVNSRFT

INADTGLITAPFGLDYENDQHDYTLTIRATESEANPLSSQVQATITITDSNDCTPQFTKT

EYEPSQAIPETASVGTQVVQVSATDCDSGTSGQLRYSIQSEYFKIDEVSGAVEISKQLNY

ENKQVYSFIVTVQDLGTPSRSSQARVTIPVIDTNDEAPKFSLENYRFSVDENAPGGETVA

TVIAFDLDSKVITYTIIGGNTEGNFVIDSVSGVIRLRSNPNPNFRQRSYSLRVAASDSRL

TGYTNVIIDINDINDHKPVFTQCNTYAPTIAEHQPSGSTVLTVTASDDDEGSFGLISYSL

VLDANQPKHFNIDAKTGAITTNREFDRERQSLYSITVRATDGGNNSSLSEVMSGFCTFNV

RVTDINDNSPSFEFKQYFGSVLLNVPINTRIITLVASDNDTGNSGAVTYSLDQSDDALYF

QIDPTNGILSTARSLDGNKREYRFLAVAIDNGQPPLQSVAPVVIQVQTNNAARFTQDRYQ

AQIVESANVGTIVATVNAYMAGDDSNAWIATYALEPGNLPKTNNPPKFSINIITGQIGVT

DSLDYEVTPQFELKVIGRLDNGNQQVTTFSTVIIQIIDVNDNPPRFTLALYSQYVRENTP

VGTPLEGNQIKAIDRDGNSSTLIIYSLVEDIPASRGKFLINARTGQIRTAVVFDRESSLR

YYTVIVKATDNGKPPQSSTSSVRVQILDVNDSPPRFAEQYYTATVAENVETGYVVTTVTA

TDEDSSENTKLSYFFIKGNHDSRFAVDNNGRITVEKYLNYEKETSYSLTLQAFDGRFYGT

TIVNISVTDVNDNRPQFTKFSYTARIKEDTPIGTTLFNIYATDPDKTVGNLLTYSLRGQG

ITDVDPPLFIVEQTGVVKTNARLDRETKDVYEFSCVATDNGEPQLSSFADCRVYLDDVND

ERPYFPDQPYIGYALENAPPPVNVRPVTALDNDIGNNSRFIYSLVDDADGTFTIDADNGM

IKTQRQLDREAVANYTVIVRATDRGQPPLSGQVNVLIQVTDSNDHSPHFKTSMFSANISE

HAPLGFPILRLEASDNDIGSNGALLYSIISGNTPVQFAVDAESGLLTVSENKLDYESKQF

YNLTVMVQDRGVPTLKDTAYIEIYIGDFNDIPPRFTNPTSQNITISEAARPGLFVTQVIA

SDPEQGRNGELFYSIVAGDPIQSFAIDPLTGVITVRLPLDRESKLIPDHYYTLTVRATDR

GQPPLYSEIPVYVYLDDVNDNGPVFIPLRYHGKIMENRKKTQNVTQLKAIDPDSANNGAP

FTFTLLGSDNDTRHFTLDSRTGVLQTIDRLFDREKKSQYRLTVRATDSGIPPMSAVTQVL

VTVMDENDNQHRTGIVNIDIVGYNDKFNGGNVGYVYSPDPDDDRNQKNYSIISGNEERWF

SINRTNGMIYCRPGLPMNKEFRLSVRVSDGKFADVTSSVVIKSESRSTDALINIASIQAA

GINPNDFVSYYYESFRKAVANILRTTTPIIVDIINVQAANPSQSTAQAIDIMFAAHGSPY

YAKDVIESRLMMQKASLEEQTGLYINQIGVDACQTNNCLSGCRTNVTTNNSINFVSPGSK

SFTGVDVVVKPVCYQCNFIPVLQNCQANHLPCLHNGKCQQSQTAGAICQCLPGYDGQYCQ

WRTRSFQGNSYFWLRSLPKCNQLTLEMEFTTTNSEGLLLYTGPVSGSNGNFLAIELIGGR

IEVIFSVSTSSNSAASGTLVVANGNLNDGRWHKVKFQYDGKILSVIVDDCLKAPLIDVNG

TLAQDRSKCSASRILPVAARYIAGNAPVQLGGLYIDAYIVQNKNLRVPSYQGCIRNFKVN

GELQDLDNPLRESRTSRYCSSTTECSSSCNNNGKCVRSWSRSICECNLGYSGDSCQIKTV

SARIPNNTRGYIRYDISQLWTVTYSKFEFKVITRDRSNSLLLFVDTGSSNSLEMIQMVNG

SIRYIVQLPSYPAQSTTIDVACDQGTWCLITAERIGNVASLKVNNKIKYFQLSDASQDQL

LIISGSERYQLYFGGVPERFNIRTNSSENFMGCVDDFRYNGRTILANQSVSGVTQDFVDS

IYLGCRSMGCIRKSVCPSNLYCDDLWDAHRCIDICRTRNPCQNNGTCNIANSQFQCSCHH

GHFGPECQFGGPAVQMQEILTYSGIGGGAILAFLCILIICLIIQSRNKRKIREKLEYAKK

GSANIATYRIEGGGEDDVDNYDLNILKNPLPDSISSLPVKIPLSPSNSSKRSYSYNLRQM

SNGSVVIKNGLPSSKSIDPDELMKRYVRDDSVKYYADEGKSTPAGSLSSLEVIEDDEVME

DDQASFSYLNVWGPRFKHLADIYNTPSVEDIPSKSENV

>A.digitifera|scaffold 876|genscan|4181_aa

MVYRKLSSMQRTLCMFYVCLSASILPVTSDTTYSFRLQEELPADTFVGTINVQQGQISGN

SDQFFSVSRSGDITTTLPVDRESLSSNPIVFDVRSSSSAVTVRIHVDDINDNIPEFPSPV

LPLRIFENDQVNSEYSIDLATDADAAENGTVDYAIISGNEDGKFRLGRNATECSTNGFSL

CIVTEGSLDRENVSFYRLNISASDRGKPSQRSFCLVNITIVDLNDNSPVFSKSIYSVSVN

ENNPVGVQILAVTATDKDQGVNGKIEYSFQNDPGSSKFQVNTSSGVISARTPLDYDVQGQ

KSYSFKVYARDRPGKPDFRQATADVVVNILDVNDNIPQLQVFYSQGKSPAEVSENAVIGT

VIATVLINDGDDSSGPNGQVYVEIANGNGTFELVYITMTPTGRFIYQLKTATSLDRERIA

FHNITITARDGGSPSLNSTVNVLLSVKDVNDEVPTFSKPRYTASVSEQAQNGSSVLRVVA

HDSDTGSNAQISYSIISGNELHWFGIDSASGLITTENSIDRESVPQGDEGKFDIDGSSGV

IRTLASLDSMVKGFYKLIIAAQDMGGKFAQQNASVEVTVQGQLDNPPQFEYAVYNFSVYE

NVAQRTYIGRVVATSKDNNASMRYTIVSGDPGQLFSVDSVEGIIMVHGKLDRETKDKYSL

NVIAQVGYVRPLSGTTTVFIEILDSNDNSPQFSPTSAAVTIDASWPAGKQIYVATAFDKD

AGLNGIVHYQLTDDGNGLCKVNTTSGAIFLARRVTSIDDSQLVLSMLASDLGSPPLYAVF

MLRVVIVSNNPPRFLASSFTVNISRDVPIGKQILPVTALDPDSGTNGMVAYAIAPTGNEE

GLFGISKKGILLVNKNLDLASVLHSLTVTATDKGETPLTSSTSVTVRIQDSISYQAIFKN

QTFVFSVMENQLPAGERVCVVTALDKDIGQNAEVEYSILTGNIDGAFQINISTGVITTLK

TLNRTQISNYTLTVSASDKGLPSRKSNCLVTITVLDSNNHLPQFSRPYYALSVVEGTAIG

TILGTVTATDDDHGENARITYTIAGDHPSDEFRINPFSGNLKVARKLDRETVEVYILNIS

ASDHGTPPLSSFVEVYVNVLDKNDNRPLFSQSVYTASISEAAALQSSIATVLATDKDFGT

NALITYTILSGNSDRTFSIYPNGTIYNLKTFDRERKSSYFLTVMARDKAEPVMRQLSSTT

VVQLTITDINDNSPYFISSNVTHVSEHAKTGDAVMKIMVADLDEGSYSLISFSLGNFGSA

TPFTLGSTDGVLRVSGSLDRELRSSYELKVTASDQGIPPKNAEMKLTVIVDDFNDHAPVF

QRRTNTVTIKEDILIGSEVARFYATDADQGRNAEVRYSIAAGNANGTFEMDPLAGVLSTI

KSLDRETIPDYTLLIRASDLGIPELHSEETLKIILSDVNDNAPTFPRASYSANVYENQVK

ANVITVVAADDDEGSDGLVSYQIIYGNEEGVFTMDSQSGQIGARVALDRETQAEYFLRIR

AKDGGTPPLVGETYVTIKVTDVNDNSPIFQPNVLKAAVKENLPAGAPVAQVGATDVDHGS

NARITYSLLVTLDLFTIEPNTGEITTTVSLDREKTPSYELVILAVDGGIPRREGNATLFI

AVEDANDFDPVFAFKQYSATIAQGAPPGTFIIMASAKDNDIGPNAESVYTVSGLPPVFQI

APRSGIITVAETVPMNRSSYSFTVKATNVNAAQRFDTTTVRITAAPGSYPVFQHFDLNLT

VSEAATLGTKLVTVNATGHTSYFIAAGNVGEVFEIDKVNGELKIVKHLDYESRSSYRVVI

GARDGSVQSLASFVVLHIAVIDVNDNAPVFNQSVYLVDIQEDLPFNSTVMWLYANDRDSG

PNAEMEFKMVSERSPASSAFHVSLTSGRVYTKIKLDRENTSSYSFKVRVEDVSDRSMASE

AVVIVNVQDINDNIPVFVPPLSGSLHENVSRGYQIAVLMATDADAQNDAQLRFGFAAGGN

PGDMFNLDARSGNLTLKGTLNREDKFQYVLRVTVDDSLHTTVSNFTVTVLDVNDNPPRFL

SNPITQKITEKLPVQTVVLNLTALDDDVGANAEILYFILPSPSSDFFKVDRQTGALRLEK

MLTYKKPRLSSNGNQYNITVKAWNPYAPFYETTADVIIEVTDANDHAPVFLSPRFDFFVL

VTARINESVGRAEAVDEQDDGLNAFVIYEKLVGNGSLLFNVEQDSGNVTVAGSLDTPGLF

YLQVRARDLGHPVMESKADVYVEVIEPNNHSPIFGLNQYRKTVLESLAVGREVIQVTASD

QDSGKNGQVFYHVERSDPPGYLGIGRQSGSIYVEKPLDYETARLIKLNVLATDGGRKPRS

SSVEVRIELMDVNDNHPVFTKREYDGYIAENTAQGTAVITVTAVDPDQFEGGRVEYGIIG

VDVLEFFEINKTSGEITSKVSFDYEVRQLYEVIISAKDHGNPQLYSQPNAKVLVHVTSVN

EYVPRFNQSLFTASVAENAPVGQLVTRIAASDKDKGPDGEMVFVLVGDSNNKGFSLDSSS

GVLSVSGRLDSERAGIVTLQVVVKNALQNIVTPDTSDVARIIITVTDANEAPYFIQKVYN

SRVKEDSLPGTFVQKVTAIDDDSAKHPVTSKITYGISTGNIGNAFAIDADTGVISTLAKL

DRESVAQYHLTITATDQGLPPLSGNGTVSVIIEDVNDNAPRLFANCSGYIRENQPRGTLV

MKLQPYDPDVDPNRGPFTFSITGTDFGKFLLDNGSGEITTTVRLDREAASSYNLSVRISD

GGSPPQSAVSFCSILVEDENDNRPRPAKRVVHVNTNGSFGAGFIADVKPEDPDADDNLVC

QIIKNSYDLFSFPPRSCLLTTKRRFNGSAKLDLRVNGSDGRWAVSYDVEVRFVTFNSKTI

DNSITVRLQNTSPTVFLLQSYQLFLNAVDRVLLQRNTYEAQLFSIKSSGSGLLDLLVAAR

NQRFDYMIREDLSAALSKNKSALERNSKVEIQTVDYTPCSASNPCQNGGECTSYIHTLGT

KSYEAQPVIFLSLDYEWRFSCLCKPDFAGEKCELSKQGCVSKPCKNGATCIEKGSSFVCQ

CPTGFRGPTCADDVDECLQSPCKNGGTCKNIVGDYQCNCLPGYLGKSCSSGFDFCRVASP

TKWAQPKCTCASSQACPCACVGFESAAFLQFPTLQNLQRGAFNNITIEFSTAKKNGLLLY

NNDGQYDKHSDFIAIQVINGRVRMSFYLGDTASAVIVEAPNFVADGQWHRVTAVRDKKVG

KVSVDSSSPVTNSSPGNLLKLNLGSSPLYVGGIKDFEQAVNHTGKHIQTDDFLGCMRNVF

INGKKLELSSSTDSAGIRDRCPRLGQCLAGQCKNGGTCLDYWFDYICQCAEGFSGRNCEQ

AVHELKIEFERSFQSKEYNLLRISVIIPSMLIIFQELMQPTLKDVICYLTERPHYQLYAS

ENTWKTRSKASCGELLDETLTRNRAMLNLRLNQNRLCTFKRLDRCELSIDHMYNADRRRL

QNDRVKPVKFGTNSFLVLQFTERYRREQQRKDQQGTPARKRRASSSSSEKFSIQFRTRRD

GLLLLAMDSAMISDSGYSALEIKSGNIEYSFRKGGNTASFTVSASVTDGEWHNVSLLHQR

KSVTVTLDGEATIKEFETAIHDILGKNIKSWHLGGNGKTMFVSATLNVDRTLAKTTPLVS

HRKTEVSNATALEISEEKHAQYQVFAGLIHAVTKTAVLVIQDRTGVLKLVNRPSGDGSLS

PGVIAAIIFFAVLLVLIVAAVIIARRVRRRRGTSDKAPLEYDANKAAEVASDLHDVSQKI

SPCHSSDDSGVVIRNPSQKSLTELRHATLQNGRDIVIHNVGAPEDYQIKLTKTSREKIDL

GFSESDREFIMLNDSVLTSDTPGIITDVARYSRTPNRRSTPLENEASRHTANKGARNRGY

QFPNPQFRHNLDKRQPLSRSVLDPRLRGPTKSSSSRRFRKGSLGSSSSDEAPDFSDTGHR

SDENNSERLEHYDIEVASLGYMKFPISTIRTLFRDHSLNLREPPGLSAAEIERLRRQTPS

GSLLDAVSSISGEGAPTIDKLSSVLEVPDTSSESSDDTFTCSEFEYDNDELSREENDRGS

MVFSKLAGGETMLSENNLSDRTELEGRASRLGSLSTLNFSDDEIIPTAFSNKPMNGSKGV

FNWDDVLNWGLRYHNLRGVYKDIAQLKDSSNPTLSNDGEYV

**Predicted Four-jointed sequences**

>A.californica|AASC02049795.1|GENSCAN_predicted_peptide_1|695_aa

RLNVKARQAVSEDDNDNDERKKEAARQSRRTPTFLPTTKNLVPTAGHRRVVCSPCRAVQS

SLTFALGGPSSVACGRPSSCCDAPRRRMKLVLVVMTGIGFTLGVVFGLLLQLPIDTSLPA

DPLLPGYPDSHFRFRSRRSLSDGDVVSPGVGGGSGKLSESLSRKGERFVKNAQNDVIVPR

NGETLDGATGVDGASANRNDLETRRVAQSAHGGVIPPGDVGETASSESIKGNGGSRLGTK

PKLLANSEPGDNPIIGWPVGGRRPNNGQIHVPREGLQRSAKENRGSAAATGPETAVRSDQ

AQTSRHPSKVLGTGGEESLNRGDATRQSAQAGDRGKNSALRMTINLRKDIVGSDTATNND

RRAEGRNNTLDTSKVAFQKNSEEDLAYLSTLVSGVTWRPQLESSCPRPFSLSEVEQWRRR

VDSLDVVKMEEGCGRMQNRLLTLRDASKACARYRLNTDQIQGEIFSYYLARLLNISNIPP

TLLVRVDALSPKWRTVHLDMSLAQWADNKLVVLTQFIPDLTPAHIPGEFREEDHRLEPTV

AHLGGKSKDELCELVQWSDLIVLDYLTANLDRVINNMFNRQWNDQMMNNPAHNLEKFSDG

RLVFLDNESGLFHGYRLLDKYFRYHKSLLHSLCVFRPGTVQAVKRLHASESIGSELHALF

SENEALHEHLAGVPDKNVKILKQRLDDVYQQIVTX

>B.mori|BAAB01080387.1|GENSCAN_predicted_peptide_1|493_aa

MENLQTNDRKHARLAAVEELAVKVLDDSDIKEKYRAYSIYSYCLLSVLISFVLGLSIGVL

ITRPDPKRQTDRITKARQGRRLDTANVSYTGVAFVNNQRSDFNRQIYPKHLNDDLRDILK

DNKGTNVNFPRTPNENTVLYDNVYWGPEIENSMPQGYGENSAELWERYVGSSEVVKMEIG

CGRMQNRLVTFRDGTRACVRYRQNTDQIQGEIFSFYVGRLLNLTNLAPSVVKIVDLKDKL

WRNVVNDIAAAKWSRNRAIVLTQYVPSLEPAHIPDVFKSSTRHLNKIDVYNMSNEENETS

DITKELLIEKMRIKDVRSKAKNIEKFSRIDMRLNDKTVKSFLELAQWSDLIVFDYLTANL

DRIVNNLFNYQWNENIMNGPAHNLAKKMDSGLLVFLDNESGLLHGYRLLKKYNVYHSLLL

DSLCVFRKSTIDALKRLHYKQDVGAKLSDMFHLRNSAVVRDVLPALPEKNSKVLHERIGR

VLSQVQKCEEALR

**Predicted Expanded sequences**

>L.gigantea|jgi|Lotgi1|166291|fgenesh2_pg.C_sca_57000067

MFKSNSSTQVTTSGKKRKVVQVILLTGEDTIVHVDIKSKFQEVFNQVAGNLSLREVEYFG

LAFKKENEYQFVALDEKIHKQVPKKWKNGQGEGYDSQNEAIVKVWFRVQFYVDQVVLLRE

KVTRHLFYLQLKENVLQYNNITSEEKCFQMVGNALQADFGSYTPDKHQDGYFDPRVYFPA

WIVAKRGMDYILQNAPKIHQENRNLTRPDAELKYIKEASISPSAHNLHFYRVRKKKTDKV

LNTWLGICPRGIEVYEEETNGFKNLISTFLWLDIGRLYFDKKKFEIRSEGCPDGRKFTYY

TDSDVTSKYLLTICRATHMFQLEIEPKLREIRHLDAEVNIQNHHEVFALEIYEIILSCQI

DHLVISQSSDGNKRRYRESYIYSDARDLVTNGNWIQHRPVKLSPSKSGSSGGGNQRYSVV

SDVSSNTTSGIVSDKMTISFDESDDHKEILIDTPPGSGSSTTPSTPRSKLSHIINSFHNY

KKSSGQSPASSPRSLELYKPRSPGITPTQPMSPVTSPGSYRSVPVYGSRNPGFSLHSPPL

IKSDRESKSRPVSRNVSRKESLKNQLASQNVENRTDATPASPSAESKALSLGISVLPNKP

TCPPFVSSAPTSFTQPFLPIATHKASDTTPSPSSSSGIGLTPGSEISLPMYSVGTPNSEA

SLNLYPIGTPVVDSSLQSYPIGAPGIDSSLPSYPIGYSSSNIPVLPGSETKAVYNIGNQN

TRQSHGLPDSVLPGYNLGLPKTISGKSPTEPIPINNIHGLPIATGGISPKDASPYAIGYQ

HPTGLTPQEAHAVYNLSFSAPSSGMASVDSTNGAYNPNYTNPIPPQSPPPYHLSFPTTST

NIPTVEATPIYHNVGFVTQNPTPTYINLPDPPPYSTAMSQLASEKMPSPGTNLEGFNLVP

KNPNPPKFSPGITPLSDSSYQPVKTDPQEIYSPPLAFADPGVVTEIPNNRIQTAQSVMVT

DGADGQREDGTKKERKKEEKDGTYIKKKQPSQHHHISKKNHVTEKLHPELLNIRSHAQSF

PLITALCNDTTLMHTNSSSTGSYDTSTMRSNNSRLSGSSVDLDSRRWSACDSGTMVTIPS

IRPKSWHSEHFDLDSQIQANHGYLFSNNFQNHQSVPQNLYGQSSPPPPTWTNAVTSSTDF

KGSTEKYPSDILTNQHMIPAKLNSGGHNKADDVNIKTLKETFGMA

>A.californica|AASC02014942.1|GENSCAN_predicted_peptide_2|1363_aa

MASKQAAASGKKRKIVNVHLLNGDERVIHVDVKSKFQEVFNQVAGQLCLRETEYFGLAFR

KDNEYHFIVLDEKIHKLAPKQWKSGSGEGTDSQGKALINLWFRVQFYVDQVILLREKVTR

HQYYLQLKENVLQYNHLYNEEKCFQLAAYALQADHGNYLAERHQGGYFNPALYFPQWMLD

RHGVDYLANNMPTIHKDLHNLTKNDAELRYIRNASMPPGGHNLHFYTVRKRKSDKVCDTW

LAICAKGVEVYEDDAGYKNHISTFLWKDIAKLYFDKKKFEIRSVQSAGGRRFMYYTDCDV

KSKYLLTICRSTHMFQMAIQPKLMEIHHLDNEGLSSSFAGFFQKVLATLNEKRVLEKVQR

LYEKTTYGGFRDKDPQSLKRKTWKTGQTSISSKRTLKNERMTPNGNQKRYRESYVYSEGR

EKCNLQHSPSKSMSVTGSSTNQRFSVISDASSNTTSGIVSDRMAVSFDDGDDHTREFMID

CPPRSAVGGTPSQIRSKFQPSFRIFTFSSHVVCTSSYWGLKELSPTSSTGSWRLRHHPGA

TPMRLMSLPSPTTPTQDTPGKGLRRSGGRRDSFRSLLLTSGERNTSGDSAVSLSGIFCGS

GQDKLAPLSLPQSELRGCLSGSPGPGGSVQHGGGGRGSGGSSTYSLTPPLNLAHLKKTGA

GPSPPTSLNFAGVSSGGMKDSSSPRGGPGAYSLGRRVQGERSPREQQLKSLGSPRELCQQ

QQPAYKLLPSPLDISDKLSLSSPRANIHEHYPKPVGSPRGEGREVTSHKTLNSPQDLGGK

AYNKPLPSPRDKISEQLKLSGPSTSSTHHAMLDFPHTLPASPSPRATESVYSFPIISALQ

QQQQQQQQQPVQTQSPQPQQLTNIQAPLPPPAEVGAGEFHQSRGVEQQPLEGSSSGVLTD

HTLLASLMQHQQQQQLTAAEKQSVHLQQQQQHQQPLFKSPAPAPSATSSSAVTDHYLLAL

RQGNAQLAEKSPSAQRAGMGGIKNILEKLPAPQAGLTMPLADHMRADQKQQHQQVAQPLD

QRLQQQQQVAQPLDQRLQQQHQNQQQYQLQDYIPYGVAVKDSSPQNKQIISSDAGAEMEE

NKENVYHQANDFNSGRDEQQEELMEEPVVAEEECERSSSTANISEAESRNSESTSHSGNK

GVLHPELKQILGQSHAISLPLITALCNDSSLVQASRSQSGSRSSYDTSTMRSTDSRLTRL

SHDTDPRRWSSCCQAAGGGVPEVMLSVQSSRPYSWHSEHFDLDSHSATHPRQLALLPAPS

EEPNLPTTSPPNPRAVLINNKQLALAGDNSEVGGGKNNVLLLPQKMIESDPGPSSPHTKS

GYSVNISCTCPTNIRAPVACSIFSITTTPTPTTTISININSSR

**Predicted Kibra sequences**

>A.digitifera|BACK01008813.1|GENSCAN_predicted_peptide_1|1006_aa

MPKKKTCEVPLPLGWEEAKDFDGRVYYIDHNTKRTSWVDPRDRLTKPMTFADCVGNGAQQ

LEDPRIQWKQRRETMLTDYLTVAQSDLKVQFWTSELEKMQRHQERQPKHIKQGSLSSSSS

SSTVNSITSKYDLNQLKVEISQAKSRLEGLKEEMAVVSGEVQAQEEGYNRLKKVDRKLSS

NGGCRREDIPNLVEEIKSMQRSLFRKEQEKKELVQALLRLKDNLSFPDSQEGRGGSDAVK

ASAGSQMDLGSDSAIGRSVEQFPRRNKQELKAEYKDALKTESKLRSEISQLDERISRVIT

EDNDSRPGLLLEKETLLDELRRLNSLVRTEGEKQRLEKLAADLDNARELSQRIVADKAQL

QNEKTKLTQQLAEQARLTNLLNIQLKCLSASTPSVSPCSSRGSLSLSGSRGSLSASSRGS

LNSLNYPDMNHSGLSEPFNLRELHQRVTDMLQGMSREAVTPCPPIRTTGAATSYTLTSGR

ASHASNSSQVSLSSRDSMSLSSHSPPVSPITSDLSPAHSPATARIISSEGESTSGLDVTE

LERNPLPLAEGIPVQQRLRLLNTDSRSNESINTVPFIPLSPITEGIAALPPTVTRPVTAA

PSDESVAADSGVFDASLENLKAVQSSADLRTRAEFEEDSAETSQVKIGLTYDAGREALVV

CIDQAKGLKALGNTSQCKTVYIKAALLPFASGENCILETKPRDYQESPEFGEQFHIPLAE

GKLTTKTLQVNIFAVNTYAVEELLGGAQISLADFNIQTPVRFRWYNLLSCAFFHSAAAKY

QRKNSVVAPSVSSVSPNGASESKRGNLRPNSWCEGSTLSLESAAGFERMPCRSVSQEALN

EPVKNLHVRKQTPGKPVQRAQSQGDSVISGGSLQDRDSDFERSPLGRTRPPVVKTKYSRK

FSDPQPLGLFPGHGAEDEVPMMPLSRMPYDINLNRSNSDCTTRQTDVSPFVRMSAERTSM

RRKKRPVSWQGLQQYQQLSPLFGDGNSKSATVAELDVHQEASRAKQ

>A.californica|gi|225540751|gb|AASC02001822.1|genewise.pep

TKPQTFADCVGDELPLGWEEIYDPNVGVYYINHINQINQLEDPRVQWRQEQEHMLKEYLV

TAQEDLEAKKEIYSIKEQRLLLAQDEYQHLNDTLSGWKSSRTSLNSNSSVGSTKYDPDLL

KQDVNLAKSRVARLKRELEQIGAEMSYQERGVETLSSVGQKLSSISGGYSLEQAQRILSE

IRQLQSRLSQGEKEKNDLMQVSLHL

>B.mori|gi|54095327|gb|AADK01014385.1|.pep|genewise

KKEMYDVKKQRLCLAQDEYKHLNNALSTLAASRTSLCSSTTSMTTTSTTSRHDPELLRSE

VTQARGRVAQLRKELRQARAEVASARRGVDTLAEVEQNLAHNXGCYNITEAQAIMTELKN

VQKSLTSGEKEKADLMQSLAKLKDDLTRLQLGDASPDLSSTLSFPQEKLSTASQTDLCAD

LVPIGTRLAEMARVRLQYDEARKRIQQIQQQLADLEDKVQPGQAESDKDRLLLFQEKEQL

LRELRSITPRTRSKQEMSDIQSECKRLEQDLKNAFEMSNKCIADRLRLHEEKQLLLQQLK

DALTSMTTLEGQLKTLSASTLSVSSSSSLGSLSTASSKGSLSSGISFTDIYGGPQIASSF

QTDKPIDMVDLHRRVERLLRGTSYNADSVTPGASSSPSQPSLSPRSSLSSASPPPPPPSY

H

>L.gigantea |jgi|Lotgi1|71556|gw1.42.139.1

TKPQSFADCVGDELPVGWEETYDPSIGVYYINHIQQTNQVEDPRLQWRQQQEVMLKEYLV

TAQDDLEAKKEIYNVKEQRLVLAQDEYSHLYNTLSGWKSSRTSLNSNSSVGSTKYDPDLL

KADVTHAKDRVARLKWELQQISAEMQQQERGVEKLTRVDEKLSGMNGGYSVTQAQGILTK

IRQIQMAMSSSEKEKMELMQTLARLKEEFLLSRYGGSSPDVSTLSLNQDRSTTASQTDLR

GEFGLSQSRYIVEKARLRLQCDEAYQKLSDLKKKLATVEDKMIPGQTESDKDRLLLLQEK

DQLVRELRSIDPKGRSEDEMTSIRQRIRQLEQDLQTGMELSNKQIAER

**Predicted Merlin sequences**

>A.digitifera|gi|342276823|dbj|BACK01020272.1|.pep|genewise

ILDLCVGNHELHIRRRKPDTMEIQQMRSLAREDKMRRQIERDKLMKEKQTREEIMKQRDE

LEKKLLEFQEEASRVKEALIKSEEMGELLAEKARVAEEEAILLGKKASDAEEEIKRLRLC

LSKQSGDERIAIERRAREAGEERFKRVLQEAEAREALILREDLMKSRQAERLAKQKLVEI

TSS

>A. queenslandica|gi|296312284|gb|ACUQ01000970.1|.pep

YSLQGKATGKQLFDLVTRTLGLREVWYFGLMFTDAKGFKTWLRYDKKIIDQHIPKDSVVG

FKFRPKFYPEDVSTLIMEVTIHLFYLHCKEDIIDERIYCSPEASLLLASFALQSEYGPYD

ESVHIPGFLNNQHLLPSNVITQFDLTPQQWEVQLIDLWTKHTNMLKSEAELEYLKIAQDL

EMYGINYFEITNKKGSQLWLGVDAYGLNIYNHDNKVQPKISFPWGEIRNVEYHGKKFCIM

PEDRNSPDFVFYVPKTKISDLIMQLCVGNHDLYLKRRKPATMEIQQMRAQAKEERSRKIM

ERDKLETEKAARMQLEREKKELEERLIALEKENQQAHQAKLAAETRAQIMEERMKIAEEQ

THSLTRKKIETEAEMQRVRASAIK

>A.californica|gi|225490095|gb|AASC02045771.1|.pep

TKASKGNAKQFPVRVTTMDAELEFTIEPKTTGKELFDMVGRTIGLRETWYFGLQYVDSKD

YVAWLKFDKKVLDQGISKESQIQFTFLAKFYPEEVSGELVQEITQHLFFLQVKQSILNMD

IYCPPEASVLLAAFAVQAKYGDYDESTYQPGMLASDDLLPQRVLDQYQMTPAMWEDKIKF

WYAERRGMTSDEAEMDYLKIAQDLEMYGVNYFQIKNKKDSDLWIGVDARGIGVYDRDNRL

LPKVAFPWSEIKNISFNDKRFTIKNVNKKEPHFVFYAPKPRINELILELCMGNHELYMRR

RRPDSMEIQQMKEKAMEEKARKQMDRSRLAREKQLREEAVREKEEMERRFLQMQDEVRQT

QEALARSEETAELLHEKMTVLDEEARLLTQKAAEAEAEVQRIKLTAIKTEEERMHMEQRA

HDAEILAAAILEDSENRAKEAEQLKDELMKAKLSEKAAKGKLLELTQTMSHTDYSTSIPS

YPVLASDLSPTLQYVESGAPLPDGDIFSGNLDQLSLEMEKERREYLSKSKTLQDQLKDLK

SEIQVLKVEDRSSTLDRLHDESMQRGDSKYATLRKVSLSAL

>B.mori|PROTEIN:BGIBMGA014502-PA

MPPFRRKKAAKSFPVKVCTLDAELEFNLEWRATGRDLFDLVCRTIGLRETWFFGLQFEDT

KHFISWLKLDKRVQDQCVSQMPGTPFMLLCKLYPEDVAEELIQEVTQHLLFLQVKQAILS

MDIYCPPEASVLLASYAVQAKYGDYDESAYKPGMLASEDLLPQRFIDQYQMTPEMWEDRI

KIWYADHKGMSRDEAEMEYLKIAQDLDMYGVNYFAIKNKKDTELYLGVTALGLNIYEKDN

KLTPKTTFPWSEIKHISFDDKKFIIKFVDRSVTNFIFFSPKGMNKLILDLCIGNHDLYMR

RRKPDTMEVQQMKAQAKEEKQRRQIERNKLSREKQLREVAERERAAMEQRLLQYQEEIRL

ANEALRRSEETAELLAEKGRVAEEEASLLAQKAADAERENARLRLSAIKTEEEKVHLERK

TREAEYLTARLVEESEKRAAEAERLKAELLTARVAEKQAKEKLLNFLSRTSTGSLPQQFP

ASPTVPCSLFPSTCSLPAELGGEGLQNGAAGAPEPELNSYQLVPDDSDPHIHRLSLEIEK

ERFVTIYVSEYELIL

>L.gigantea|jgi|Lotgi1|103995|e_gw1.2.844.1

MKTKKSGASSKSLPVRVITMDAELEFDIDNKTIGKELFELVCRTIGLRETWYFGLQFVDS

KGYIAWLKFEKKVLDQDVPKEAPIPFTFLAKFYPEDVSEELIQEITQHLFFLQVKQSILN

MDVYCPPEASVLLASYAVQAKFGDYDESTYRPGRLASEDLLPQRVIDQYQMTAEMWEDRI

KVWYADRRGMTRDEAEMEYLKIAQDLEMYAINYFQIKNKKGTELWFGVDCRGINVYERDN

RLSPKVTFPWSEIKNISFKDKKFTIKNVDKKAPDFMFYAPKSRINKLILELCVGNHDLFM

RRRKPDSMEIQQMKAQARDEKTRKQLDRSRLAREKQMREEAMREKEEMERKLASLQEDIR

GAQEALIRSEESADLLAEKVRILDEEAQLLTQKASEAEGEVNRMKLTTMKTEEERMIMEH

RAMEAEFIAVTLLEDSEKRAKEAEMLRNELVQARISEKAAKGTLLELTRDTPHYTVRLSS

DLTSELRLNSDLGSGDQDEMSCDLIPADMDHDQLSLEIEKERVEYMEKSQHLQEQLKGLK

SEIEVLKVEDKQTDFDRLHEESIERGENKYSTLSKITASTLRSRVTFFEDL

**Predicted Scalloped sequences**

>Acropora_digitifera

LSPKTAKISDHEDLEMANSVDEGNSAKSPVDMANDAEGVWSPDIEQSFQEALAIYPPCGR

RKIILSDEGKMYGRNELIARYIKLRTGKTRTRKQVSSHIQVLARKKAREIQGKLKDQAAR

DKALQTMASMSSAQIVSASVLHNKALAAQGMPPATYNNESQGYQQQYWQSGATAPPVPSN

YNNASQSMYGSPGVQATVPGNVVSTASVASPTGAVVSSPDDLRSASYTSGAVSSTPPTFA

AHGSSSWPPRTAVQEHQHLCLVSSRFVRQSVQSVMGKDKQRQSRALRCLEPYNNSPHGRP

KDRASGSSGVVHTVHDSSTEYGHYENNRFVYRIHRSPMCEYMINFVHKLKQLPEKYMMNS

GVQQGNTRNFVVSCLCFRGFHK

>Aplysia_californica

XRNELIARYIKLRTGKTRTRKQVSSHIQVLARRKAKEIQAQLKVTTNLQDQAARDKALQQ

MASMSSAQIVSASAIHNKALAGGLGGLPPSMASGPVRPPYPGAPSIWQPGLSQGDVKPFA

PAYGFDSKLPSSISGEMALPPWQGRSIAGPKLRLVEFSAFLEQPRDPDSYHKHLFVHIGS

TATYNDPLLEAVDIRQIYDKFPEKKGGLKELYDKGPASAFFLVKFWGDINTNLQDEAGAF

YGVTSLYENNENLTIEVSTKVCSFGKQVVEKVETEYARMENGRYIYRIHRSPMCEYMINF

IHKLKHLPEKYMMNSVLENFTILQVVTNRDTRETLLCIAFVFEVSTSEHGAQHHIYKLVK

D

>Lottia_gigantea

MEDLQDAEGVWSPDIEQSFQEALAIYPPCGRRKIILSDEGKMYGRNELIARYIKLRTGKT

RTRKQVSSHIQVLARRKSKEIQAQLKDQACRDKALQQMASMSSAQIVSASAMHNKNIASG

LSGLSGMSPVRSGFPGATPVSWHVLECVKPFGSPSYGLDAKIPPSIPGEMTPAWQGRSIA

GPKLRLGEFSAFLEQQRDPDSYHKHLFVHIQSNPTYNDPLLESVDIRQIYDKFPEKKDGL

KDLYDKGPQTAFFLVKFWADINTNVQDEAGAFYGVTSQYESSENMTIQVSTKVCSFGKQV

VEKVETEYGRFENGRFMYRIHRSPMCEYMINFIHKLKHLPEKYMMNSVLENFTILQVITN

RDTRETLICIAFVFEVSTSEHGAQHHTYKLVKD

**Predicted Salvador sequences**

>Caenorhabditis elegans|T10H10.3:T10H10.3 peptide: T10H10.3 pep:KNOWN_protein_coding

MLFSRRKPVTSVEGTQGKYVKRESPPTVPSLAATAVSRTRRSPIAASTLILSSVPPPPTS

TPPPEIQMYHSENPSVEEADDLDDIEHQEDFFDLNDLKEMEFERLHSRSTASENDVYTEP

SDEPSTSFGARNNYVLSSRSFNRGALRYVPRKSLTASHSIGNVEQGTRELSVIEKRLASP

QPPRFQSLQTLPSEHLMTIQQYRSSCDCSSDIPLPENWAVEFTTENQPYYVDHANRRTHW

VHPFVHESLKPGWKKFFDPEKGVFYYHEEMKRTQYEHPGISNPIFRTESVNVASRSQVDL

NANLHIIEEKELPPWLLMYAQSDSSLDHLLEWDLFNFEQLTEYEHLMMKLYKQEVFDIVK

KYEKKRNVLNREIHRRDVSRPPPARINEN

**Predicted Mats sequences**

>Acropora_digitifera

MSFLFGGRSTKTFKPKKNIPEGTHQYDLMKQAAATLGSGNLRLAVMLPEGEDLNEWVAVN

TVDFFNQINMLYGTITEFCTVESCAVMSAGPKYEYHWADGTTIKKPIKCSAPKYIDYLMT

WVQDQLDDETLFPSKIGKMESSKKKRLNKECKRNIDYGSNVSLNAFTSERGLRNCLLAK

>Aplysia_californica

SSQTFRPKKRFESGTLKYNLHKQANASLNSGIDLKEVVKLPPGEDVNDWVAVHVDFFNRI

NLLYGTIFEHCTEQTCPTMSGGPYEYHWCDGVNYKKPTALPAPQYITLLMEWVEAQINDE

NLFPVQVKPFPRNYLSIAKKILKRLFRVFVHVYIHHFDKLLAIGAEAHGNTSYKHFYYFV

SEYNLIDKKELEPL

**Predicted Warts sequences**

>A.californica|gi|225473600|gb|AASC02062266.1|.pep

PGFSSYGLSQRPPNFGPSSPAASETNSVRSDSPSHNPTSQRPFLTNGLQTPPPLPPRMPI

TTQANHTPRGQTPPPSIVGGGGAVDAAVKSGGNPVAQKGHVQQMIQRISPGQAYYFQQQK

QQQQHHQQHHQQQQQQQQQQHPHQQVNGGGGGGGAPPGQQQQQQDFHRSSHTAALDLSKL

SIAGGGDAQQIIQKKVMATSGVPATRRPMSSSQMPVIMSSVHSKEVQKPIAQTATAPLVP

PPHTVNPNGAAGSPQSYLSHHVQAHINNSNISNSNIISNSPMSALHQQHQQLKQHQQQQQ

QQQQQQHSHQQSPRAIPASRGEVPVQITHSYPQHPAHLHSPLPTNHIHQGVKLPPNSGVG

GNGGGGGGGGLAMYPPQYPSEMANPVSRSDSPVSRATNQSPQSVISTTSSPSTNSDIPDK

PPPPYPGRAGIAQEKHRCMSPLPERRSDARERDSLRRDTKVRMYSPQAFKFYMEQHVENV

LKSHSQRMNRRLQLEKEMAKVNLSEEAARQMRKMLHQKESNYIRLKRAKMESSMFEKITT

LGVGAFGEVALVRKKDVGSLYAMKTLKKANVIKRNQVAHVKAERDILAEADNEWVVKLYY

SFQDRDNLYFVMDYVPGGDLMGLLIRREILEEPLAQFYIAELVLAIESVHRMGFIHRDIK

PDNILIDSDGHIKLTDFGLCTGFRWTHNSKYYQK

>A.californica|gi|225473600|gb|AASC02062266.1|.pep

PGFSSYGLSQRPPNFGPSSPAASETNSVRSDSPSHNPTSQRPFLTNGLQTPPPLPPRMPI

TTQANHTPRGQTPPPSIVGGGGAVDAAVKSGGNPVAQKGHVQQMIQRISPGQAYYFQQQK

QQQQHHQQHHQQQQQQQQQQHPHQQVNGGGGGGGAPPGQQQQQQDFHRSSHTAALDLSKL

SIAGGGDAQQIIQKKVMATSGVPATRRPMSSSQMPVIMSSVHSKEVQKPIAQTATAPLVP

PPHTVNPNGAAGSPQSYLSHHVQAHINNSNISNSNIISNSPMSALHQQHQQLKQHQQQQQ

QQQQQQHSHQQSPRAIPASRGEVPVQITHSYPQHPAHLHSPLPTNHIHQGVKLPPNSGVG

GNGGGGGGGGLAMYPPQYPSEMANPVSRSDSPVSRATNQSPQSVISTTSSPSTNSDIPDK

PPPPYPGRAGIAQEKHRCMSPLPERRSDARERDSLRRDTKVRMYSPQAFKFYMEQHVENV

LKSHSQRMNRRLQLEKEMAKVNLSEEAARQMRKMLHQKESNYIRLKRAKMESSMFEKITT

LGVGAFGEVALVRKKDVGSLYAMKTLKKANVIKRNQVAHVKAERDILAEADNEWVVKLYY

SFQDRDNLYFVMDYVPGGDLMGLLIRREILEEPLAQFYIAELVLAIESVHRMGFIHRDIK

PDNILIDSDGHIKLTDFGLCTGFRWTHNSKYYQK

>B.mori|PROTEIN:BGIBMGA006330-PA

MNPPAPGKTATRSGYHQKALAEIRNSLLPFANIGNSEPPGSSAASTVSSGVSSGFSSSSG

NGLDKDLNVLPQSLNQLIALGYDEDPAVRALKYAGGRFDAALDYLSKQQEPLNGVLKSSN

LSALGTKLIRKPSLEREINLHRGSPALDSGAGSSRSDSPRQSEPPPLPHEKLSRQYSPSG

FSEPPPPPPPRCPSTPPVLPSVQQLLKRMSPAPPLPPARGTSPVAAAAPTPPARQPMIVQ

NGPQVQQQLTQQIQALSIYQTGGGGELPPPYPMSGAPPPPPYSVSIQNRQSPTQSQDYRK

SPSSGIYSGGTSAGSPSPITVTQSTGSAAGMTRPTPLQAWTARQAVQPPIIMQSVKSTQV

QKPVLQTAIAPVAPPPATSAGAPPPPPSYASSIQQKQAQTPPSYPSAPKPSSPGSTPTAI

PPAVPTTEPPSYAITMQVLAVQRGMHPVPPPPYGNQADNTTTVNSHHSPLHKKISNNCDG

KPESSQGPHEIKCPNQNCTNNLLKDNVTASGSDKGSNGSSDRRPKMLDKIRHQSPIPERR

NYSKEKEDERRDCKVRNYSPQAFKFFMEQHVENVLKSYKQRLFRRMQLEKEMSKIGLSAE

AQCQMRKMLSQKESNYIRLKRAKMDKSMFTKIKPIGVGAFGEVTLVKKIDTSHLYAMKTL

RKADVLKRNQVAHVKAERDILAEADNEWVVKLYYSFQDKDNLYFVMDYIPGGDLMSLLIK

LGIFEENLARFYIAELTCAVESVHKMGFIHRDIKPDNILIDRDGHIKLTDFGLCTGFRWT

HNSKYYQRNDHGRQDSMDPVDGEWGAMGECRCYQLKPLERRRRREHQRCLAHSLVGTPNY

IAPEVLQRTGYTQLCDWWSVGVILYEMLVGSPPFLAPTPAETQLKVINWESTLHVPDAAN

LSPESKDLILQLCSGQDTRLGKDANEVKNHPFLKGIDFDKGLRNQVAPYIPRIEYPTDTS

NFDPIDPDKLRNSGSSDSNKSDSELLDNGKTFHGFFEFTFRRFFDDGYTSKINLDDNDNQ

GPVYV

>L.gigantea|jgi|Lotgi1|177509|fgenesh2_pm.C_sca_6000054

MTRCRLDYHQRALAEIAESLRPFQTVNSDTSSTSSANSTVNGDSDTKQGLSNPGYDEWGL

GQYPKVIRKQSFESKYGPSSPAASDTNSVRSDSPGIGNVHQRHILIENLKPMANGHQTPP

PLPPRAPIVPPQTPPRGQTPPPNSQGEIIPVSYPGHVQHMIKVMSPIQVQRENAANQQAL

YRTHFIQQQQQQQQQQQSQPQSQMQLTNGANQIVSQGISPVTSLSRQVYGHNGHTSALMH

QIPRGTTLTHNGTTNTISQTSGLPPPMGATNPPIIMHSVKSKEVQKPVPQQATVPTLPPS

NNHVTTSNQNYISSVQAQVNGNNAQNIPVLQQQANNNNLRKLQIQLKGQNGLNMLTPEQL

QALASQQAQIKIHIQNPTSHNPQNHIQSVQVQNIQQNLPPNYDFYIPRSWPDTPGSTPRS

DSPVSRATNQSPLSVLSTTSSPSTNSDIPDKPPPPYPEQTHRCMSPMPERRPEARERDKL

RRDTKVRNYSPQAFKFYMEQHVENVLKSHDQRQHRRFQLENEMTKVGLSDEAATQMRRML

HQKESNYIRLKRAKMDKLMFDKIITLGVGAFGEVSLVRKRDPKTLYAMKTLRKSDVIKRN

QVAHVKAERDILAEADNEWVVKLYYSFQDRDHLYFVMDYVPGGDLMGLLIKMEILDETLA

RFYIAELVLAIESVHKMGFIHRDIKPDNILIDHDGHIKLTDFGLCTGFRWTHNSKYYQKD

GSHARQDSMEVSSCNLDEHCRCEILKPLERRRNRERQRCLAHSLVGTPNYIAPEVLLRQG

YSSVCDWWSVGVILYEMLLGHPPFYASTPIETQTKVIHWKQTLKIPSDTHLSHESKDLIL

RLLCSAEDRLGVSGAQEVKNHAFFSSIHLDGLRKQAAPFIPTIRYPTDTSNFDPVDPEKL

RNDSEETWKRPDSKLENGKHPEHAFYEFTFRRFFDDGGHPCPLPDPNSPVYV
